# A highly convergent synthesis of the C1–C31 polyol domain of amphidinol 3 featuring a TST-RCM reaction: confirmation of the revised relative stereochemistry†
†Electronic supplementary information (ESI) available: Experimental details, spectral data, correlation with the natural product and copies of spectra. See DOI: 10.1039/c5sc00814j


**DOI:** 10.1039/c5sc00814j

**Published:** 2015-08-06

**Authors:** Aleksandr Grisin, P. Andrew Evans

**Affiliations:** a Department of Chemistry , Queen's University , 90 Bader Lane , Kingston , ON K7L 3N6 , Canada . Email: Andrew.Evans@chem.queensu.ca ; Tel: +1 613 533 6286

## Abstract

The concise enantioselective synthesis of the revised C1–C31 fragment of the polyketide amphidinol 3 was accomplished in 16 steps and 12.8% overall yield. Salient features of the strategy include chemoselective Weinreb amide coupling and concomitant CBS reduction for the preparation of the C1–C15 *tris-syn*-1,5-diol motif and a temporary silicon-tethered ring-closing metathesis (TST-RCM) reaction in combination with a diastereoselective hydroboration for the construction of the C16–C31 polypropionate fragment. The union of the fragments was accomplished by a regioselective ring-opening of the terminal epoxide with a phenyl sulfone stabilized carbanion, which upon reduction and deprotection permits a comparison of the relative configuration with the natural product.

## Abstract 2

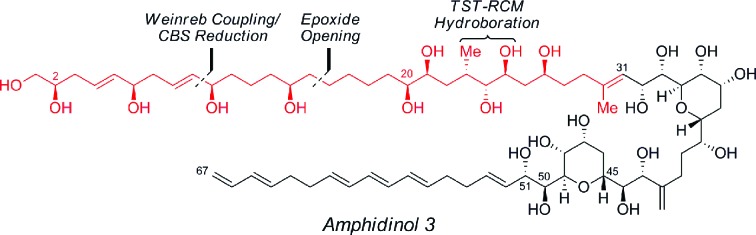
The concise enantioselective synthesis of the revised C1–C31 fragment of the polyketide amphidinol 3 was accomplished in 16 steps and 12.8% overall yield.

## Introduction

**Fig. 1:**
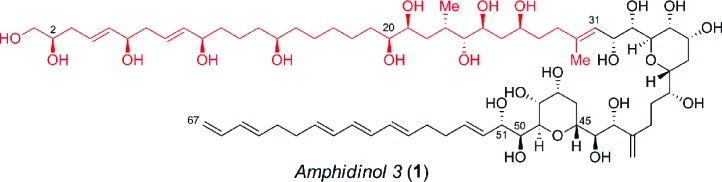
Structure of the polyene–polyhydroxy secondary metabolite, amphidinol 3 (**1**).

Amphidinols (AMs) and their congeners are structurally unique polyene–polyhydroxy secondary metabolites that belong to the linear polyether family isolated from the dinoflagellate *Amphidinium* species.^1^ In recent years there has been considerable interest in amphidinol 3 (**1**, Fig. 1), which was isolated in 1996 from *A. klebsii* in waters off the coast of Japan, due to its complex architecture and potent biological activity.1*c* For instance, the amphidinols exhibit antifungal, cytotoxic, hemolytic and anti-diatom activity, in which AM3 (**1**) exhibits the most potent antifungal activity (MEC = 4–9 μg per disk against *Aspergillus niger*), albeit with hemolytic action (EC_50_ = 0.009–0.4 μM against human erythrocyte cells). Interestingly, the mechanism of action for this agent has recently been attributed to its ability to form *barrel-stave* pores, similar to amphotericin B, which is induced by the stereospecific molecular recognition of membrane sterols.^2^,^3^ Specifically, the *bis*-tetrahydropyran core, which is highly conserved in this family, hydrogen bonds with the 3β-OH of ergosterol and cholesterol to permit the permeabilization of the membrane. The absolute and relative configuration of AM3 (**1**) was deduced using a combination of *J*-based configurational analysis (JBCA) for acyclic 1,2- and 1,3-dioxygenated systems, modified Mosher's method, NOE experiments and chiral HPLC analysis of degradation products.^4^ Nevertheless, the revision of the configuration at C2 and C51 has severely hampered progress towards the total synthesis of this agent.^5^ Hence, the unique molecular architecture and potent biological activity coupled with residual structural and mechanistic ambiguities have prompted several creative approaches^6^ to the C1–C31 polyol,^7^ C32–C51 *bis*-tetrahydropyran^8^ and the C52–C67 polyene,^9^ albeit many of which were accomplished prior to the stereochemical revisions outlined above. Herein, we now describe a novel and expeditious synthesis of the *revised* C1–C31 fragment of AM3 (**1**) using a highly convergent strategy, that confirms the relative configuration of this portion of the natural product.

## Retrosynthetic analysis

**Scheme 1:**
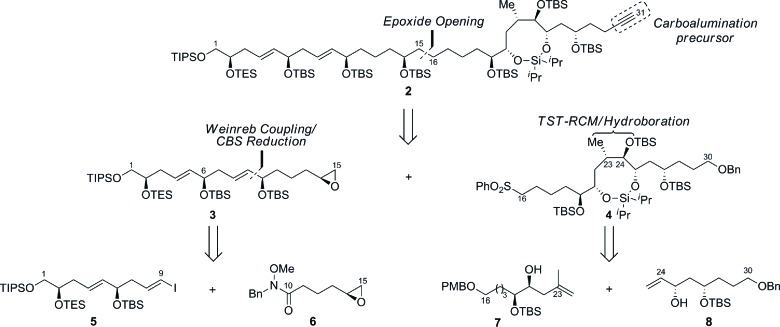
Retrosynthetic analysis of the C1–C31 polyol fragment of amphidinol 3. TIPS = triisopropylsilyl, TBS = *tert*-butyldimethylsilyl, TES = triethylsilyl, Bn = benzyl, PMB = *p*-methoxybenzyl.

We envisioned the C1–C31 fragment, which is challenging due to the complications posed by the installation of remote stereochemistry in the acyclic linear carbon backbone, would be derived using the strategy outlined in Scheme 1. For instance, this motif has three *syn*-1,5-diols, two of which are separated by *E*-configured double bonds, coupled to a highly functionalized polyacetate/polypropionate type domain that is terminated with a trisubstituted *E*-olefin.

Hence, the ability to develop a highly convergent route to **2** would provide an opportunity to facilitate a Negishi carboalumination/Cram addition^10^ to enable the union with the C32–C67 segment and elaboration to the natural product. The retrosynthetic analysis of **2** affords two fragments, **3** and **4**, of similar size and complexity, which we assumed could be coupled *via* the ring-opening of the terminal epoxide **3** with the lithiated sulfone derived from **4**. The masked *syn*-1,5-tetraol **3** would in turn be prepared by the alkylation of the Weinreb amide **6** with an organometallic reagent derived from the vinyl iodide **5** followed by an enantioselective reduction of the resulting ketone. The preparation of the cyclic silaketal **4**, which constitutes the aforementioned polyacetate/polypropionate type domain, relies on a *Z*-selective TST-RCM reaction for coupling **7** and **8** with concomitant diastereoselective hydroboration to facilitate the construction of the C23–C24 stereocenters using medium-ring stereocontrol.^11^,^12^

## Results and discussion

**Scheme 2:**
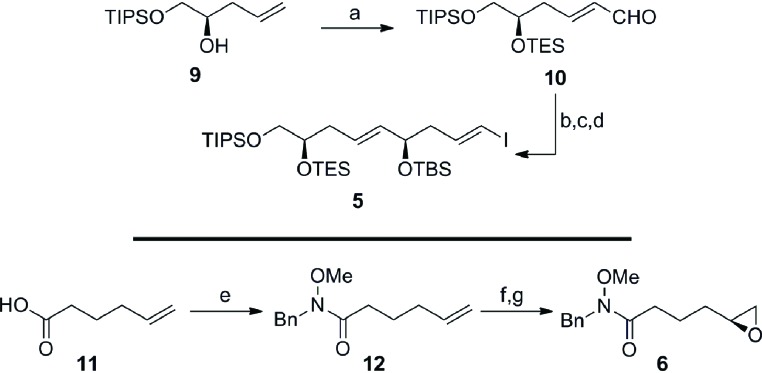
Preparation of the C1–C9 iodide **5** and the C10–C15 epoxide **6**. Conditions: (a) Acrolein, HG-II, CH_2_Cl_2_, 40 °C, then TESOTf, Et_3_N, CH_2_Cl_2_, –78 °C, 93%, *E*/*Z* ≥ 19 : 1; (b) AllenylSnBu_3_, (^*l*^Ipc)_2_BH, Et_2_O, –40 °C to –20 °C, then **10**, Et_2_O, –78 °C, 89%, *ds* ≥ 19 : 1; (c) TBSOTf, Et_3_N, CH_2_Cl_2_, 0 °C, 95%; (d) I_2_, Et_2_O, 0 °C, 99%; (e) CDI, then BnNH(OMe), CH_2_Cl_2_, 0 °C to RT, 92%; (f) Acetone, Oxone^®^, NaHCO_3_, EtOAc/H_2_O (1 : 1), RT, 98%; (g) (*S*,*S*)-Co-OAc, H_2_O, THF, RT, 60% (based on 50% conv.), ≥99% *ee*; HG-II = Hoveyda–Grubbs’ second-generation catalyst, Tf = trifluoromethanesulfonyl, Ipc = isopinocampheyl, CDI = 1,1′-carbonyldiimidazole, Oxone^®^ = potassium peroxymonosulfate, THF = tetrahydrofuran.

Guided by this strategy, we began our synthesis of the C1–C15 fragment **3** with the preparation of Weinreb coupling partners **5** and **6** (Scheme 2). Cross metathesis of the homoallylic alcohol **9**^13^ with excess acrolein using Hoveyda–Grubbs’ second-generation catalyst,^14^ followed by *in situ* protection of the secondary alcohol furnished enal **10** in 93% yield (*E*/*Z* ≥ 19 : 1 by NMR). Treatment of the α,β-unsaturated aldehyde **10** with the chiral tin boronate derived from the combination of the allenyl stannane with (^*l*^Ipc)_2_BH in diethyl ether at –78 °C, afforded the requisite vinyl stannane in 89% yield with excellent stereocontrol (*ds* ≥ 19 : 1 by NMR).^15^ Protection of the resulting secondary alcohol as the *tert*-butyldimethylsilyl ether and halogen–metal exchange of the vinyl stannane gave iodide **5** in 94% (over 2 steps), thereby completing the pronucleophile component. The preparation of the enantiomerically enriched Weinreb amide **6** originated with the conversion of 5-hexenoic acid **11** to the Weinreb amide **12** using carbonyldiimidazole and *N*-benzyl-*O*-methylhydroxylamine.^16^ Epoxidation of the terminal olefin in **12** with *in situ* generated DMDO provided the racemic epoxide,^17^ which was subjected to Jacobsen's hydrolytic kinetic resolution to furnish the enantiomerically enriched epoxide **6** (≥99% *ee* by HPLC).^18^

**Scheme 3:**
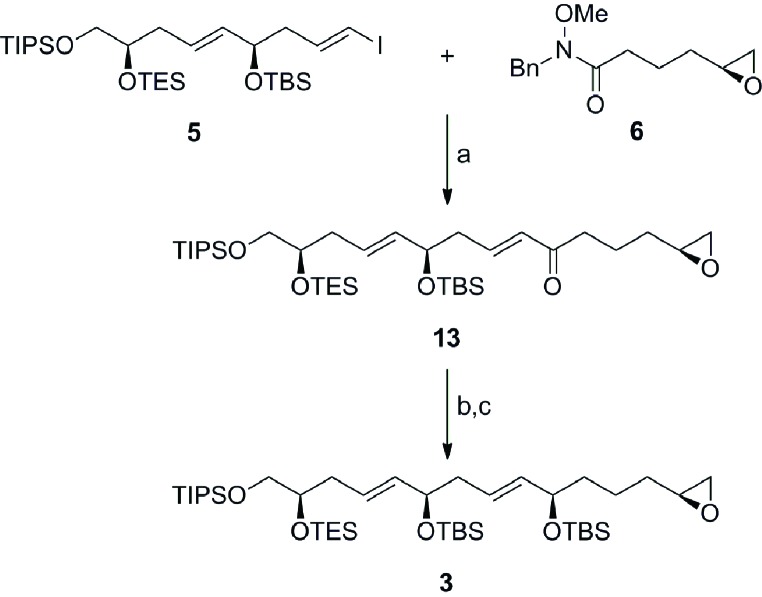
Preparation of the C1–C15 fragment **3**. Conditions: (a) ^*i*^PrMgCl·LiCl, 15-crown-5, THF, –10 °C, 64%; (b) (*S*)-Me-CBS, BH_3_·DMS, THF, –40 °C, 99%, *ds* ≥ 19 : 1; (c) MTBSTFA, DMAP, MeCN, RT, 99%; (*R*)-Me-CBS = (*R*)-methyl oxazaborolidine, DMS = dimethyl sulfide, MTBSTFA = *N-tert*-butyldimethylsilyl-*N*-methyltrifluoroacetamide, DMAP = 4-(dimethylamino)pyridine.

Scheme 3 outlines the coupling of the vinyl iodide **5** with the Weinreb amide **6** and elaboration to the terminal epoxide **3**. Preliminary attempts to facilitate the coupling with the vinyl lithium reagent derived from **5** proceeded with moderate success, due to the reduction of the intermediary organometallic reagent. Gratifyingly, treatment of the vinyl iodide **5** with *^i^*PrMgCl·LiCl in the presence of 15-crown-5 followed by the addition of the Weinreb amide **6** furnished the α,β-unsaturated ketone **13** in 64% yield without erosion of olefin geometry.^19^ The fragment was then completed with the enantioselective CBS reduction of ketone **13** (*ds* ≥ 19 : 1 by NMR) and protection of the allylic alcohol to afford the C1–C15 fragment **3** in excellent overall yield.^20^

**Scheme 4:**
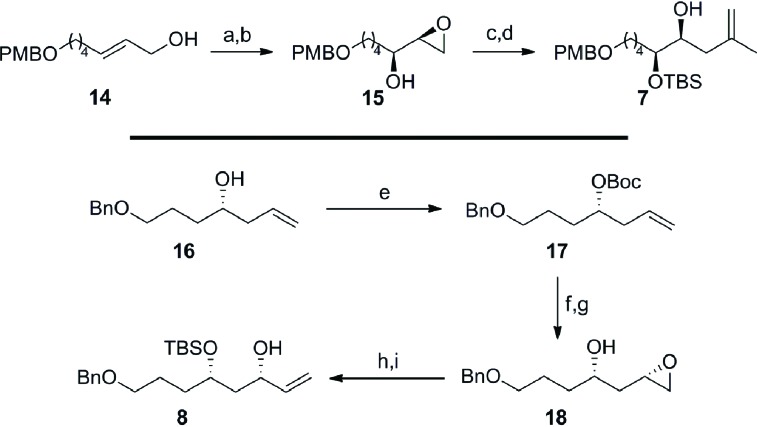
Preparation of the C16–C23 fragment **7** and the C24–C30 fragment **8**. Conditions: (a) Br_2_, PPh_3_, imid, 2-methyl-2-butene, CH_2_Cl_2_, 0 °C; (b) AD-mix-α, *^t^*BuOH/H_2_O (1 : 1), 0 °C, 75% (over 2 steps), 92% *ee*; (c) TBSCl, imid, CH_2_Cl_2_, 0 °C to RT, 80%; (d) Isopropenylmagnesium bromide, Li_2_[CuCl_4_], Et_2_O, –78 °C to RT, 99%; (e) Boc-ON, LiHMDS, THF, 0 °C, 95%; (f) IBr, PhMe, –85 °C, *ds* = 15 : 1; (g) K_2_CO_3_, MeOH, RT, 81% (over 2 steps); (h) TBSCl, TMEDA, DMF, 0 °C to RT, 97%; (i) Me_3_SOTf, *^n^*BuLi, THF, –10 °C to 0 °C, 92%; imid = imidazole, AD = asymmetric dihydroxylation, Boc-ON = 2-(*tert*-butoxycarbonyloxyimino)-2-phenylacetonitrile, HMDS = hexamethyldisilazane, PhMe = toluene, TMEDA = tetramethylethylenediamine, DMF = dimethylformamide.

In concurrent work, we focused on the preparation of the fragments required for the key TST-RCM cross-coupling reaction (Scheme 4).^21^ Conversion of the allylic alcohol **14**^22^ to the corresponding primary allylic bromide and concomitant Sharpless asymmetric dihydroxylation,^23^ afforded the required α-hydroxy epoxide **15** in 75% overall yield and with 92% enantiomeric excess (by ^1^H NMR analysis of the Mosher's ester). Protection of the secondary alcohol **15** as the *tert*-butyldimethylsilyl ether and regioselective ring-opening of the terminal epoxide with isopropenylmagnesium cuprate at –78 °C furnished **7** in 79% yield over two steps. The preparation of the allylic alcohol **8** commenced with Boc protection of the homoallylic alcohol **16**^24^ to afford carbonate **17** in 95% yield. This substrate provided the necessary functionalization to affect the strategic 1,3-*syn* stereoinduction using IBr at low temperature to install the C25 stereocenter with good diastereocontrol (*ds* = 15 : 1 by NMR).^25^ Hydrolysis of the intermediate cyclic iodocarbonate with potassium carbonate in methanol furnished the β-hydroxy epoxide **18** in 81% yield over two steps. The allylic alcohol **8** was then completed in 89% overall yield with the protection of the secondary alcohol **18** as the *tert*-butyldimethylsilyl ether and ring-opening of the terminal epoxide with the sulfonium ylide, generated *in situ* from Me_3_SOTf.

**Scheme 5:**
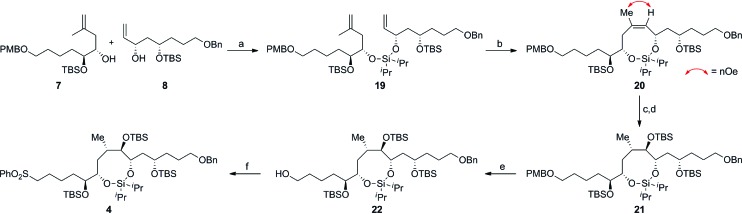
Construction of the C16–C30 fragment **4** using the TST-RCM/hydroboration reaction. Conditions: (a) **7**, ^*i*^Pr_2_SiCl_2_, imid, CH_2_Cl_2_, 0 °C to RT, then **8**, imid, CH_2_Cl_2_, 0 °C to RT, 84%; (b) 2 × 15 mol% G-II, CH_2_Cl_2_, 40 °C, 97%, *Z*/*E* ≥ 19 : 1; (c) BH_3_·THF, THF, RT, then H_2_O_2_, NaOH, 0 °C to RT; (d) TBSOTf, Et_3_N, CH_2_Cl_2_, –40 °C, 72% (over 2 steps), *ds* ≥ 19 : 1; (e) DDQ, CH_2_Cl_2_/pH 7 buffer (20 : 1), 0 °C, 87%; (f) PhSSPh, PBu_3_, MeCN, RT, then TPAP, NMO, 40 °C, CH_2_Cl_2_, 76%; DDQ = 2,3-dichloro-5,6-dicyano-1,4-benzoquinone, imid = imidazole, TPAP = tetra-*n*-propylammonium perruthenate, NMO = 4-methylmorpholine *N*-oxide.

**Fig. 2:**
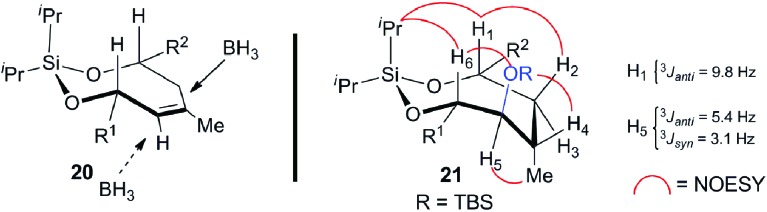
Model for the stereocontrol in the hydroboration and the NMR analysis of the stereochemical outcome.

Scheme 5 delineates the TST-RCM coupling of the fragments **7** and **8** and subsequent elaboration to afford **4**. Treatment of the homoallylic alcohol **7** with excess ^*i*^Pr_2_SiCl_2_ to afford the *mono*-alkoxychlorosilane, followed by removal of the excess tethering reagent and addition of the allylic alcohol **8**, furnished the diene **19** in 84% yield,^11^,^12^ thereby setting the stage for the ring-closing metathesis reaction. Although preliminary studies demonstrated that the cyclization of **19** was particularly challenging, Grubbs' second-generation catalyst provided the optimal catalyst to afford the silaketal **20** in 97% yield with excellent *Z*/*E* selectivity (≥19 : 1 by NMR).^26^,^27^ Furthermore, this transformation was highly scalable and reproducible (>1 g scale). Diastereoselective hydroboration of the trisubstituted olefin in **20** provided the required *anti-vic*-alcohol using medium-ring stereocontrol (Fig. 2). Although the transformation was accompanied by the cleavage of a *tert*-butyldimethylsilyl ether group, this was inconsequential since the crude diol was silylated to afford the fully protected silaketal **21** in good overall yield as a single diastereoisomer (*ds* ≥ 19 : 1 by NMR). The origin of stereocontrol in the hydroboration is evident from the inspection of the molecular model of **20**, which demonstrates the approach of the electrophile is favored from the convex face of the silaketal (Fig. 2).

The relative stereochemistry of the hydroboration product **21** was assigned using a series of 2D NMR experiments in conjunction with coupling constant analysis, as outlined in Fig. 2. The observed spectroscopic data indicates that the silaketal **21** adopts a boat-chair conformation. The 1,2-diequatorial (*gauche*) coupling constant between H_5_ and H_4_ (^3^*J*_*syn*_ = 3.1 Hz) and NOE correlation between H_2_–H_1_–^*i*^Pr–H_6_–OR–H_4_, which reside on the same face of the molecule support this assignment. Furthermore, the pseudo-1,2-diaxial *J*_H1,H3_ (9.8 Hz) and 1,2-axial-equatorial *J*_H5,H6_ (5.4 Hz) coupling constants provide additional support for this connectivity. The sulfone **4** was completed in 66% overall yield by the chemoselective cleavage of the primary PMB ether **21** followed by a one-pot Mitsunobu/oxidation sequence on the primary alcohol **22**.

**Scheme 6:**
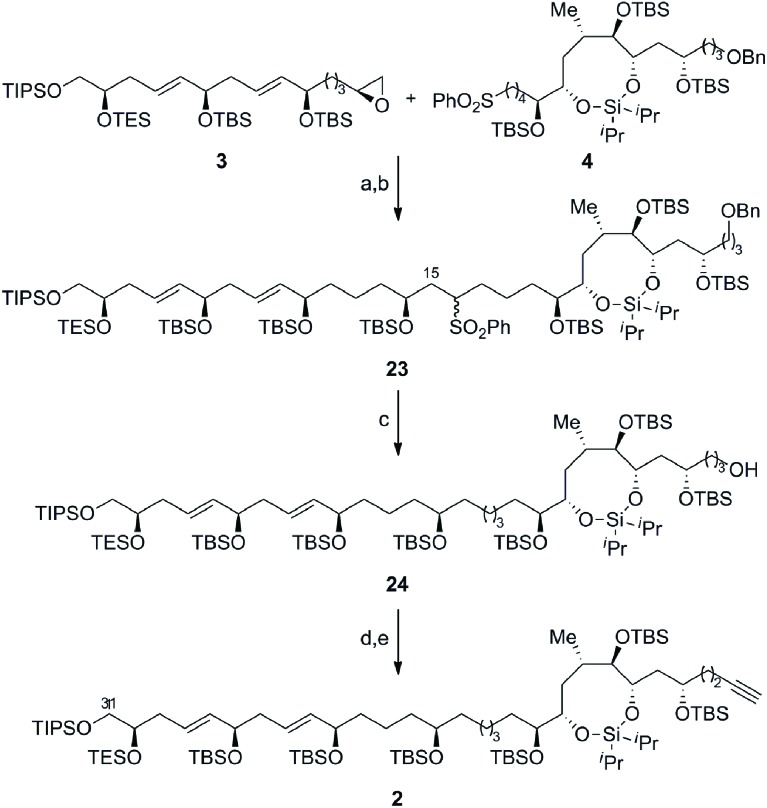
Completion of the C1–C31 fragment of amphidinol 3. Conditions: (a) **4**, *^n^*BuLi, THF, –78 °C, then **3**, BF_3_·Et_2_O; (b) TBSOTf, Et_3_N, CH_2_Cl_2_, 0 °C, 90% (over 2 steps); (c) LiDBB, THF, –78 °C, 64%; (d) TPAP, NMO, molecular sieves (4 Å), CH_2_Cl_2_, 0 °C; (e) Me(CO)C(N_2_)P(O) (OMe)_2_, K_2_CO_3_, THF/MeOH (1 : 1), 0 °C to RT, 89% (over 2 steps); LiDBB = lithium di-*tert*-butylbiphenylide.

**Fig. 3:**
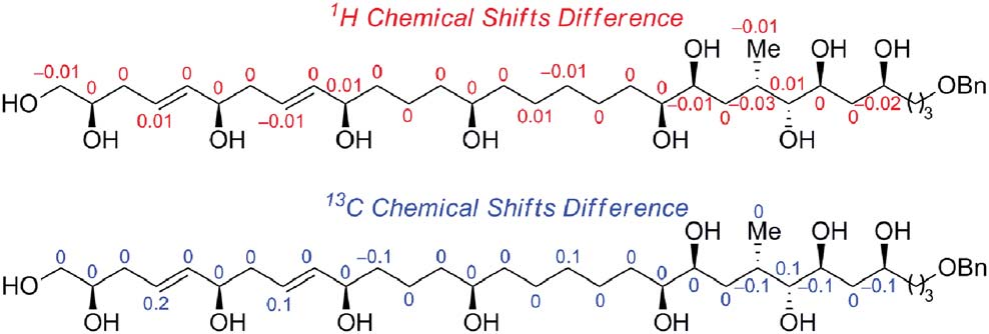
Comparison of ^1^H and ^13^C NMR data of synthetic and natural polyol fragment of AM3.

Scheme 6 outlines the union of the C1–C15 and C16–C30 fragments to complete the construction of the masked polyol **2**. Following our initial plan, regioselective epoxide opening was achieved by lithiation of the phenyl sulfone **4** with *^n^*BuLi, followed by addition of the terminal epoxide **3** and BF_3_·Et_2_O at –78 °C to furnish the requisite β-hydroxysulfone intermediate. The silylation of the latter afforded the C15–C16 coupling product **23** in excellent overall yield as an inconsequential mixture of diastereoisomers at C16.^29^,^30^ The selective removal of the sulfone and the primary benzyl ether groups in **23** was achieved using a single-electron reduction with freshly prepared lithium di-*tert*-butylbiphenylide complex in THF at –78 °C to afford **24** in 64% yield. The resulting primary alcohol was oxidized to the aldehyde using TPAP^31^ and converted to the alkyne **2***via* Seyferth–Gilbert homologation with the Bestmann–Ohira reagent in 89% yield over 2 steps to complete the stereoselective construction of the C1–C31 fragment of AM3 (**1**).^32^ Chemoselective desulfonylation and deprotection of the silyl ethers in **23** afforded the polyol fragment to facilitate a direct comparison of the spectroscopic data (^1^H and ^13^C NMR) with the natural product to confirm the reassigned relative configuration of amphidinol 3 (**1**) as outlined in Fig. 3.

## Conclusion

In conclusion, we have developed an expeditious synthesis of the reassigned C1–C31 fragment of polyketide amphidinol 3 (**1**) in 12.8% overall yield using a 16-step longest linear sequence from **16**. The strategy encompasses a high degree of convergence and allows for the convenient preparation of this intermediate for completion of the natural product. Our approach features the allylboration of an electron-deficient α,β-unsaturated aldehyde, mild iodine–magnesium exchange and chemoselective Weinreb amide coupling. Furthermore, the synthesis highlights the utility of the TST-RCM methodology for the non-aldol preparation of the polypropionate portion of AM3 (**1**) *via* the cross-coupling of advanced intermediates and a highly regio- and stereoselective electrophilic functionalization using medium-ring stereocontrol. Overall, this route provides the most expeditious approach to the polyol fragment of AM3 (**1**) developed to date and confirms the revised structure for the polyol domain of the natural product.

## Supplementary Material

Supplementary information
