# Supplementary material for: A highly convergent synthesis of the C1–C31 polyol domain of amphidinol 3 featuring a TST-RCM reaction: confirmation of the revised relative stereochemistry
Source: Chem Sci. 2015 Aug 6;6(11):6407–12. doi: 10.1039/c5sc00814j (PMC5507186; doi:10.1039/c5sc00814j)
Supplement: Supplementary file 1 [file SC-006-C5SC00814J-s001.pdf]

Electronic Supplementary Material (ESI) for Chemical Science.
This journal is © The Royal Society of Chemistry 2015

A highly convergent synthesis of the C1-C31 polyol domain of amphidinol 3 featuring a TST-RCM reaction: Confirmation of the revised relative stereochemistry

Aleksandr Grisin and P. Andrew Evans*

Department of Chemistry, Queens University, Kingston, Ontario, K7L 3N6, Canada

1.	General Information	S1
2.	Experimental Procedures and Spectral Data	S2
3.	Global Deprotection and NMR Correlation Studies	S25
4.	Copies of NMR Spectra	S29

1. General Information

All reactions were carried out under an atmosphere of argon with anhydrous solvents, unless otherwise stated. Anhydrous CH_2Cl_2, DMF and MeCN were obtained by passing degassed solvents through activated alumina columns in a Grubbs solvent purification system (PureSolv MD-6 of Innovative Technology Inc.). Imidazole was dried in high vacuum overnight; 2,6-lutidine and triethylamine were distilled from CaH_2 under nitrogen; $MgSO_4$ or Na_2SO_4 in their anhydrous form were used as drying agents; all other commercially available reagents (Aldrich, Alfa-Aesar, Acros) were purchased and used as received, unless otherwise noted. Analytical thin layer chromatography (t.l.c.) was performed on pre-coated 0.25 mm thick silica gel 60-F254 plates (Whatman PE SIL G/UV); UV light and by treatment with a spray of Pancaldi reagent [$(NH_4)_6MoO_4$, $Ce(SO_4)_2$, H_2SO_4, H_2O] followed by heating. All compounds were purified by flash chromatography using silica gel 60 (40–63 μm, SiliCycle) and gave spectroscopic data consistent with being ≥95% the assigned structure. Optical rotations ($[\alpha]_D^{20}$) were measured on a Perkin-Elmer Model 343 plus polarimeter with a sodium lamp (D line, 589 nm) at ambient temperature (indicated in °C as superscript) using a 1 dm path length quartz cell; solution concentration (c) are given in g/100 mL. HPLC analyses were carried out on an Agilent 1200 series HPLC instrument equipped with a diode-array detector using chiral column Chiralpac® AS-H (0.46 cm Ø × 25 cm). ^{1}H-NMR and ^{13}C-NMR spectra were recorded on a *Bruker Avance DRX-500* spectrometer in the solvent indicated ($CDCl_3$ or C_6D_6) at ambient temperature; chemical shifts (δ) are given in ppm and calibrated using the signal of residual undeuterated solvent as internal reference ($CDCl_3$: δ_H = 7.26 ppm and δ_C = 77.16 ppm; C_6D_6: δ_H = 7.15 ppm and δ_C = 128.06 ppm;). ^{1}H-NMR data are reported as follows: chemical shift (multiplicity, 1st order spin system if available, coupling constant, integration). Coupling

constants (J) are reported in Hz and splitting patterns are designated using the following abbreviations: s (singlet), d (doublet), t (triplet), q (quartet), m (multiplet), br (broad), app. (apparent) and combinations thereof. The reported chemical shifts for broad singlets, and each part of the 2nd order spin systems were averaged. ^{13}C-NMR spectra with complete proton decoupling were described with the aid of an APT sequence, separating methylene and quaternary carbons (e, even), from methyl and methine (o, odd). IR spectra were recorded on a Perkin-Elmer FT-IR Spectrum 100 spectrometer; wavenumbers (ν) are given in cm^{-1}; and the abbreviations w (weak, <33%), m (medium, 33-66%), s (strong, >66), vs (very strong, >95%) and br (broad) are used to describe the relative intensities of the IR absorbance bands. High resolution chemical ionization (CI) electron-impact (EI) and electrospray ionization (ESI) mass spectra were recorded on the *TermoFinnigan Mat 95 XP* spectrometer, *VG 7070E* double focusing magnetic sector mass spectrometer equipped with solid probe inlet and a *Thermo Scientific Orbitrap Velos Pro* spectrometer with HESI (heated electrospray ionization) source under positive mode.

2. Experimental Procedures and Spectral Data

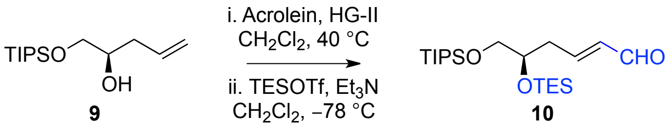

(*R*,*E*)-5-((Triethylsilyl)oxy)-6-((triisopropylsilyl)oxy)hex-2-enal (10). (*R*)-1-(Triisopropylsilyloxy)-pent-4-en-2-ol (**9**)[1] (2.58 g, 9.99 mmol) and acrolein (3.7 mL, 49.9 mmol) were dissolved in CH_2Cl_2 (25 mL) and stirred at room temperature. Hoveyda-Grubbs' 2nd generation catalyst (0.063 g, 0.100 mmol) was added and the reaction mixture refluxed for *ca.* 2 hours (t.l.c. control). The reaction was then concentrated *in vacuo* and dissolved in CH_2Cl_2 (30 mL), cooled to −78 °C and treated with triethylamine (4.4 mL, 31.99 mmol) followed by TESOTf (5.6 mL, 24.96 mmol) and stirred for *ca.* 10 minutes (t.l.c. control). The reaction mixture was then quenched with saturated aqueous $NaHCO_3$ solution, warmed to room temperature and partitioned between water and diethyl ether. The combined phases were washed with water, saturated NaCl solution, dried ($MgSO_4$), filtered and concentrated *in vacuo* to afford the crude product. Purification by flash chromatography (silica gel, eluting with 50:1, 19:1 diethyl ether/hexanes) afforded the *triethylsilyl ether* **10** (3.72 g, 93% yield) as a colorless oil: **R$_f$** = 0.86 (1:1 diethyl ether/hexanes); $[\alpha]_D^{20}$ +7.7 (c 1.0, $CHCl_3$); **^{1}H NMR** (500 MHz, $CDCl_3$) δ 9.52 (d, J = 8.0 Hz, 1H), 6.95 (ddd, J = 15.4, 8.0, 7.2 Hz, 1H), 6.18 (ddt, J = 15.5, 7.9,

[1] L. G. Quan, S. H. Kim, J. C. Lee and J. K. Cha, *Angew. Chem., Int. Ed.*, 2002, **41**, 2160.

1.2 Hz, 1H), 3.87 (ddt, J = 8.2, 6.0, 4.7 Hz, 1H), 3.68 (dd, A of ABX, J_{AB} = 9.6 Hz, J_{AX} = 4.8 Hz, 1H), 3.47 (dd, B of ABX, J_{AB} = 9.6 Hz, J_{BX} = 8.2 Hz, 1H), 2.69 (dddd, A of ABXYZ, J_{AB} = 14.4 Hz, J_{AX} = 6.5 Hz, J_{AY} = 4.7 Hz, J_{AZ} = 1.6 Hz, 1H), 2.53 (dddd, B of ABXYZ, J_{AB} = 14.4 Hz, J_{BX} = 7.8 Hz, J_{BY} = 6.5 Hz, J_{BZ} = 1.1 Hz, 1H), 1.12-1.04 (m, 21H), 0.95 (t, J = 8.0 Hz, 9H), 0.59 (q, J = 7.8 Hz, 6H); **^{13}C NMR** (125 MHz, $CDCl_3$) δ 194.18 (o), 155.85 (o), 135.06 (o), 71.89 (o), 67.03 (e), 38.15 (e), 18.10 (o), 11.99 (o), 6.96 (o), 4.97 (e); **IR** (Neat) 2944 (m), 2911 (m), 2867 (m), 2805 (w), 1696 (vs), 1639 (w), 1461 (m), 1119 (s), 1005 (s), 882 (m), 743 (s) cm^{-1}; **HRMS** (ESI, $[M+Na]^+$) calcd for $C_{21}H_{44}NaO_3Si_2$ 423.2727, found 423.2707.

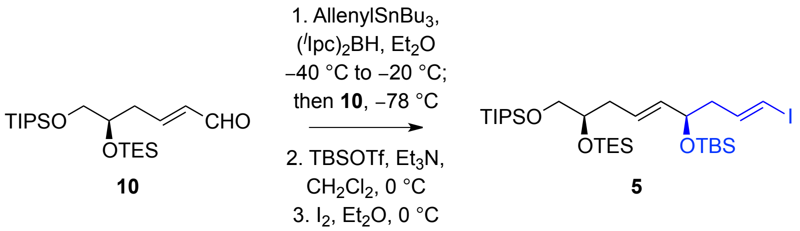

(5*R*,9*R*,*E*)-5-((*E*)-3-Iodoallyl)-12,12-diisopropyl-2,2,3,3,13-pentamethyl-9-((triethylsilyl)oxy)-4,11-dioxa-3,12-disilatetradec-6-ene (5). Finely crushed (^{l}Ipc)$_2$BH (3.32 g, 11.58 mmol) was weighed into a round-bottom flask in a glove box, capped with a rubber septum and removed from the glove box. The borane was suspended in Et_2O (45 mL) and the resulting mixture cooled with stirring to –40 ˚C. Allenyltributylstannane (5.5 mL, 90% w/w, 16.6 mmol) was added and the mixture stirred for *ca.* 2 hours at –40 ˚C then slowly warmed to –20 ˚C for *ca.* 3 hours, during which time most of the borane dissolved. The reaction mixture was then cooled to –78 ˚C and the α,β-unsaturated aldehyde **10** (2.02 g, 5.04 mmol) in Et_2O (2 mL) was added. The mixture was stirred for *ca.* 16 hours at –78 °C (t.l.c. control), quenched with MeOH (0.25 mL) and allowed to warm to 0 ˚C. Saturated aqueous $NaHCO_3$ solution (8.5 mL) was added, followed by the slow addition of 30% H_2O_2 (16 mL). The reaction was stirred vigorously for *ca.* 16 hours and then partitioned between saturated aqueous NaCl solution and diethyl ether. The combined organic phases were dried (Na_2SO_4), filtered and concentrated *in vacuo* to afford the crude product. Purification by flash chromatography (silica gel, neutralized with 1% triethylamine in hexane, eluting with 1:25 to 1:20 diethyl ether/hexanes) afforded the *vinylstannane* (3.27 g, 89% yield) as a colorless oil: **R_f** = 0.12 (1:20 diethyl ether/hexanes); $[\alpha]_D^{20}$ +1.3 (*c* 1.0, CH_2Cl_2); **^{1}H NMR** (500 MHz, C_6D_6) δ 6.24-6.09 (m, 2H), 5.89 (dt, J = 15.0, 7.4 Hz, 1H), 5.67 (dd, J = 15.4, 6.1 Hz, 1H), 4.15 (quintet, J = 5.5 Hz, 1H), 3.88 (quintet, J = 5.7 Hz, 1H), 3.77 (dd, A of ABX, J_{AB} = 9.6 Hz, J_{AX} = 5.0 Hz, 1H), 3.67 (dd, B of ABX, J_{AB} = 9.6 Hz, J_{BX} = 6.8 Hz, 1H), 2.56 (app. dt, A of ABXY, J_{AB} = 13.1 Hz, J_{AX} = J_{AY} = 6.4 Hz, 1H),

2.50-2.42 (m, 2H), 2.38 (app. dt, B of ABXY, J_{AB} = 13.9 Hz, J_{BX} = J_{BY} = 7.0 Hz, 1H), 1.67-1.51 (m, 6H), 1.38 (sextet, J = 7.4 Hz, 6H), 1.36 (s, 1H), 1.15-1.06 (m, 21H), 1.04 (t, J = 8.0 Hz, 9H), 0.98-0.90 (m, 6H), 0.94 (t, J = 7.4 Hz, 9H), 0.66 (q, J = 7.9 Hz, 6H); **^{13}C NMR** (125 MHz, C_6D_6) δ 145.92 (o), 135.83 (o), 131.80 (o), 127.24 (o), 73.46 (o), 71.83 (o), 67.60 (e), 46.93 (e), 37.91 (e), 29.62 (e), 27.74 (e), 18.31 (o), 14.02 (o), 12.35 (o), 9.78 (e), 7.26 (o), 5.46 (e); **IR** (Neat) 3433 (br, w), 2955 (s), 2923 (s), 2868 (s), 1599 (w), 1463 (m), 1103 (s), 995 (s) 882 (m), 743 (s) cm^{-1}; **HRMS** (ESI, $[M+Na]^+$) calcd for $C_{36}H_{76}NaO_3Si_2{}^{120}Sn$ 755.4253, found 755.4260. The vinylstannane (3.57 g, 4.88 mmol) was dissolved in CH_2Cl_2 (20 mL) and sequentially treated with triethylamine (2.72 mL, 19.50 mmol) and TBSOTf (1.68 mL, 7.31 mmol) at 0 °C. The reaction mixture was stirred for *ca.* 30 min (t.l.c. control), quenched with saturated aqueous $NaHCO_3$ and partitioned between water and diethyl ether. The combined organic phases were washed with water, saturated NaCl solution, dried (Na_2SO_4), filtered and concentrated *in vacuo* to afford the crude product. Purification by flash chromatography (silica gel deactivated with 1% triethylamine in hexane, eluting with 0:100 to 1:50 diethyl ether/hexanes) afforded the *tert-butyldimethylsilyl ether* (3.92 g, 95% yield) as a colorless oil: **R_f** = 0.44 (1:50 diethyl ether/hexanes); $[\alpha]_D^{20}$ +3.5 (*c* 1.0, CH_2Cl_2); **^{1}H NMR** (500 MHz, C_6D_6) δ 6.28 (dt, J= 18.9, 6.3 Hz, 1H), 6.17 (d, J = 19.0 Hz, 1H), 5.90 (dt, J = 15.0, 7.4 Hz, 1H), 5.72 (dd, J = 15.4, 6.2 Hz, 1H), 4.28 (q, J = 6.0 Hz, 1H), 3.90 (quintet, J = 5.5 Hz, 1H), 3.78 (dd, A of ABX, J_{AB} = 9.6 Hz, J_{AX} = 5.2 Hz, 1H), 3.69 (dd, B of ABX, J_{AB} = 9.6 Hz, J_{BX} = 6.8 Hz, 1H), 2.57 (dd, A of ABX, J_{AB} = 13.8 Hz, J_{AX} = 6.5 Hz, 1H), 2.54-2.46 (m, 2H), 2.40 (app. dt, B of ABXY, J_{AB} = 13.5 Hz, J_{BX} = J_{BY} = 7.0 Hz, 1H), 1.71-1.55 (m, 6H), 1.40 (sextet, J = 7.4 Hz, 6H), 1.17-1.09 (m, 21H), 1.06-1.03 (m, 9H), 1.05 (s, 9H), 1.02-0.99 (m, 6H), 0.96 (t, J = 7.4 Hz, 9H), 0.67 (q, J = 8.0 Hz, 6H), 0.15 (s, 6H); **^{13}C NMR** (125 MHz, C_6D_6) δ 146.54 (o), 136.42 (o), 130.42 (o), 126.16 (o), 73.92 (o), 73.38 (o), 67.36 (e), 47.96 (e), 37.69 (e), 29.68 (e), 27.83 (e), 26.27 (o), 18.56 (e), 18.32 (o), 14.03 (o), 12.37 (o), 9.78 (e), 7.28 (o), 5.50 (e), –3.81 (o), –4.38 (o); **IR** (Neat) 2955 (s), 2927 (s), 2868 (s), 1599 (w), 1463 (s), 1250 (m), 1114 (s), 1069 (s), 1004 (s), 882 (m), 834 (s), 743 (s) cm^{-1}; **HRMS** (ESI, $[M+Na]^+$) calcd for $C_{42}H_{90}NaO_3Si_3{}^{120}Sn$ 869.5118, found 869.5119. Iodine (1.05 g, 4.15 mmol) was added to the *tert*-butyldimethylsilyl ether (1.754 g, 2.073 mmol) in Et_2O (20 mL) at 0 °C and the mixture stirred for *ca.* 20 minutes (t.l.c. control). The reaction was then quenched with saturated aqueous Na_2SO_3 solution and partitioned between water and dichloromethane. The combined organic phases were dried (Na_2SO_4), filtered and concentrated *in vacuo* to afford the crude product. Purification by flash chromatography (silica gel deactivated with 1% triethylamine in hexane, eluting with 0:100 to

1:100 diethyl ether/hexanes) afforded the *vinyl iodide* **5** (1.398 g, 99%) as a colorless oil: $\mathbf{R_f}$ = 0.44 (1:50 diethyl ether/hexanes); $[\alpha]_D^{20}$ –1.3 (*c* 1.0, CH_2Cl_2); **^{1}H NMR** (500 MHz, C_6D_6) δ 6.46 (dt, *J* = 14.6, 7.4 Hz, 1H), 5.83 (dtd, *J* = 15.4, 7.7, 1.0 Hz, 1H), 5.77 (dt, *J* = 14.4, 1.2 Hz, 1H), 5.48 (ddt, *J* = 15.4, 6.0, 1.3 Hz, 1H), 3.97 (q, *J* = 5.9 Hz, 1H), 3.86 (app. dq, *J* = 6.9, 5.1 Hz, 1H), 3.73 (dd, A of ABX, J_{AB} = 9.6 Hz, J_{AX} = 5.2 Hz, 1H), 3.63 (dd, B of ABX, J_{AB} = 9.6 Hz, J_{BX} = 7.0 Hz, 1H), 2.46 (app. dtd, A of ABXYZ, J_{AB} = 13.8 Hz, J_{AX} = J_{AY} = 6.7 Hz, J_{AZ} = 1.0 Hz, 1H), 2.35 (app. dtd, B of ABXYZ, J_{AB} = 13.6 Hz, J_{BX} = J_{BY} = 6.7 Hz, J_{BZ} = 1.2 Hz, 1H), 2.04 (app. dtd, A of ABXYZ, J_{AB} = 14.1 Hz, J_{AX} = J_{AY} = 7.2 Hz, J_{AZ} = 1.1 Hz, 1H), 1.98 (dddd, B of ABXYZ, J_{AB} = 14.0 Hz, J_{BX} = 7.1 Hz, J_{BY} = 2.2 Hz, J_{BZ} = 1.5 Hz, 1H), 1.16-1.07 (m, 21H), 1.03 (t, *J* = 8.0 Hz, 9H), 0.97 (s, 9H), 0.65 (q, *J* = 7.9 Hz, 6H), 0.07 (s, 3H), 0.06 (s, 3H); **^{13}C NMR** (125 MHz, C_6D_6) δ 143.30 (o), 135.46 (o), 126.78 (o), 77.11 (o), 73.19 (o), 72.52 (o), 67.19 (e), 45.16 (e), 37.47 (e), 26.14 (o), 18.42 (e), 18.33 (o), 12.36 (o), 7.29 (o), 5.47 (e), –4.06 (o), –4.54 (o); **IR** (Neat) 2950 (s), 2866 (s), 1607 (w), 1462 (m), 1251 (m), 1116 (s), 1069 (s), 1005 (m), 975 (m), 882 (m), 835 (s), 775 (s) cm^{-1}; **HRMS** (ESI, $[M+Na]^+$) calcd for $C_{30}H_{63}NaO_3Si_3{}^{127}I$ 705.3028, found 705.3019.

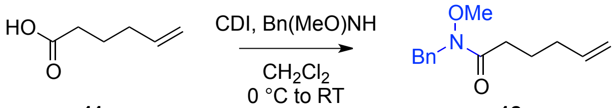

11 12

***N*-Benzyl-*N*-methoxyhex-5-enamide (12).** 5-Hexenoic acid **11** (1.45 g, 12.73 mmol) was added to CDI (2.48 g, 15.27 mmol) in CH_2Cl_2 (40 mL) at 0 °C and the resulting mixture warmed to room temperature and stirred for *ca.* 2.5 hours (t.l.c. control). *N*-Benzyl-*O*-methylhydroxylamine[2] (8.32 g, 15.27 mmol) in CH_2Cl_2 (5 mL) was added and the reaction mixture was stirred for *ca.* 16 hours (t.l.c. control). The reaction was then quenched with saturated aqueous NH_4Cl solution and partitioned between water and diethyl ether. The combined organic phases were dried ($MgSO_4$), filtered and concentrated *in vacuo* to afford the crude product. Purification by flash chromatography (silica gel, eluting with 1:1 diethyl ether/hexanes) afforded the *Weinreb amide* **12** (2.75 g, 92% yield) as a colorless oil: $\mathbf{R_f}$ = 0.19 (1:1 diethyl ether/hexanes); **^{1}H NMR** (500 MHz, $CDCl_3$) δ 7.29-7.23 (m, 5H), 5.75 (ddt, *J* = 17.0, 10.3, 6.7 Hz, 1H), 4.98 (dq, *J* = 17.1, 1.7 Hz, 1H), 4.92 (ddt, *J* = 10.2, 2.1, 1.1 Hz, 1H), 4.73 (s, 2H), 3.56 (s, 3H), 2.43 (t, *J* = 7.5 Hz, 2H), 2.07 (ddt, *J* = 14.4, 7.1, 1.2 Hz, 2H), 1.72 (quintet, *J* = 7.5 Hz, 2H); **^{13}C NMR** (125 MHz, $CDCl_3$ @328 K) δ 174.70 (e), 138.28 (o), 137.00 (e), 128.67 (o), 128.58 (o), 127.73 (o), 115.18 (e), 62.31 (o), 49.71 (e), 33.34 (e), 31.67

[2] R. W. Gantt, R. D. Goff, G. J. Williams and J. S. Thorson, *Angew. Chem., Int. Ed.*, 2008, **47**, 8889.

(e), 23.95 (e); **IR** (Neat) 3066 (w), 3033 (w), 2937 (w), 1659 (vs), 1605 (w), 1496 (w), 1440 (s), 1401 (s), 1236 (m), 995 (s), 729 (m) cm^{-1}; **HRMS** (CI, $[M+H]^+$) calcd for $C_{14}H_{20}NO_2$ 234.1489, found 234.1487.

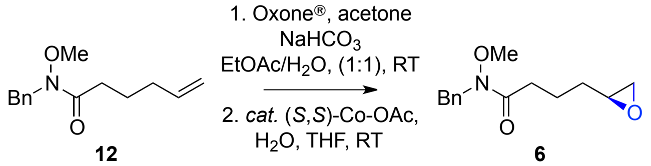

(*S*)-*N*-Benzyl-*N*-methoxy-4-(oxiran-2-yl)butanamide (6). Acetone (17 mL, 235 mmol) and $NaHCO_3$ (9.88 g, 118 mmol) were added to a solution of the amide **12** (2.75 g, 11.77 mmol) in a mixture of reagent grade EtOAc/H_2O (118 mL) under an atmosphere of air. The resulting suspension was stirred vigorously at room temperature and an aqueous solution of Oxone® (200 mL, 28.9 g, 47.1 mmol) was added dropwise over *ca.* 3 hours (t.l.c. control). Additional $NaHCO_3$ (4.94 g, 59 mmol), acetone (8.5 mL, 117.5 mmol) and Oxone® (7.0 g, 11.39 mmol) was added and the mixture stirred for an additional hour (t.l.c. control) before being partitioned between water and ethyl acetate. The combined organic phases were dried ($MgSO_4$), filtered and concentrated *in vacuo* to afford the crude product. Purification by flash chromatography (silica gel, eluting with 2:1 to 3:1 diethyl hexane/hexanes) afforded *epoxide* ***rac*-6** (2.87 g, 98% yield) as a colorless oil: $\mathbf{R_f}$ = 0.35 (1:1 ethyl acetate/hexanes); **^{1}H NMR** (500 MHz, $CDCl_3$) δ 7.30-7.21 (m, 5H), 4.73 (s, 2H), 3.57 (s, 3H), 2.87 (app. ddt, J = 6.4, 4.2, 2.6 Hz, 1H), 2.68 (app. t, M of AMX, $J_{AM} = J_{MX}$ = 4.5 Hz, 1H), 2.49 (t, J = 7.4 Hz, 2H), 2.41 (dd, A of AMX, J_{AM} = 5.0 Hz, J_{AX} = 2.7 Hz, 1H), 1.80 (app. d of quintets, A of ABX_2Y_2, J_{AB} = 15.5 Hz, $J_{AX} = J_{AY}$ = 7.5 Hz, 1H), 1.77 (app. d of quintets, B of ABX_2Y_2, J_{AB} = 15.9 Hz, $J_{BX} = J_{BY}$ = 7.6 Hz, 1H), 1.60 (dddd, A of ABXYZ, J_{AB} = 13.8 Hz, J_{AX} = 8.6 Hz, J_{AY} = 6.6 Hz, J_{AZ} = 5.0 Hz, 1H), 1.50 (app. dq, B of $ABXY_2$, J_{AB} = 14.3 Hz, $J_{BX} = J_{BY}$ = 7.2 Hz, 1H); **IR** (Neat) 3032 (br, w), 2938 (w), 1655 (vs), 1605 (w), 1496 (w), 1445 (m), 1404 (s), 1242 (m), 992 (m), 731 (m) cm^{-1}. The epoxide ***rac*-6** (2.87 g, 11.50 mmol), (*S*,*S*)-Co-OAc (0.076 g, 0.115 mmol) and water (0.114 mL, 6.32 mmol) in THF (1 mL) were stirred at room temperature under an atmosphere of air for *ca.* 36 hours (t.l.c. control). The reaction mixture was then directly purified by flash chromatography (silica gel, eluting with 1:1 dichloromethane/hexanes followed by 2:1 to 3:1 diethyl ether/hexanes) to afford the (*S*)-*epoxide* **6** (0.86 g, 60% yield, based on 50% conversion) as a brown oil: Enantiomeric excess of **6** was determined by chiral HPLC analysis and was shown to be ≥99% *ee*; Chiralpak® AS-H column, ^{i}PrOH/Hexane – 3.0:97.0, 1.5 mL/min, 25 °C, 210 nm, t_r(minor) = 27.5 min, t_r(major) = 36.0 min; $\mathbf{R_f}$ = 0.35 (1:1 ethyl acetate/hexanes);

$[\alpha]_D^{20}$ −4.0 (*c* 1.0, $CHCl_3$); **^{1}H NMR** (500 MHz, $CDCl_3$) δ 7.29-7.21 (m, 5H), 4.73 (s, 2H), 3.57 (s, 3H), 2.87 (app. ddt, *J* = 6.5, 4.3, 2.5 Hz, 1H), 2.68 (app. t, M of AMX, J_{AM} = J_{MX} = 4.5 Hz, 1H), 2.49 (t, *J* = 7.4 Hz, 2H), 2.41 (dd, A of AMX, J_{AM} = 5.0 Hz, J_{AX} = 2.7 Hz, 1H), 1.80 (app. d of quintets, A of ABX_2Y_2, J_{AB} = 16.3 Hz, J_{AX} = J_{AY} = 7.5 Hz, 1H), 1.77 (app. d of quintets, B of ABX_2Y_2, J_{AB} = 16.0 Hz, J_{BX} = J_{BY} = 7.6 Hz, 1H), 1.60 (dddd, A of ABXYZ, J_{AB} = 13.8 Hz, J_{AX} = 8.6 Hz, J_{AY} = 6.6 Hz, J_{AZ} = 5.0 Hz, 1H), 1.50 (app. dq, B of $ABXY_2$, J_{AB} = 14.3 Hz, J_{BX} = J_{BY} = 7.0 Hz, 1H); **^{13}C NMR** (125 MHz, $CDCl_3$) δ 174.17 (e), 136.63 (e), 128.59 (o), 128.41 (o), 127.69 (o), 62.17 (o), 52.06 (o), 49.15 (e), 46.88 (e), 32.03 (e), 31.70 (e), 21.09 (e); **IR** (Neat) 3033 (w), 2938 (w), 1655 (vs), 1605 (w), 1497 (w), 1445 (m), 1404 (s), 1242 (w), 991 (m), 731 (m) cm^{-1}; **HRMS** (CI, $[M+H]^+$) calcd for $C_{14}H_{20}NO_3$ 250.1438, found 250.1436.

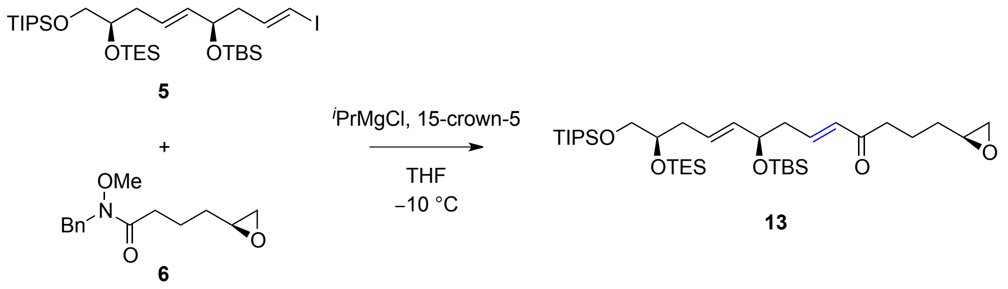

(5*E*,8*R*,9*E*,12*R*)-8-((*tert*-Butyldimethylsilyl)oxy)-1-((*S*)-oxiran-2-yl)-12-((triethylsilyl)oxy)-13-((triisopropylsilyl)oxy)trideca-5,9-dien-4-one (13). The vinyl iodide **5** (0.548 g, 0.802 mmol) was dissolved in THF (1.2 mL) and treated with ^{i}PrMgCl·LiCl complex (2.47 ml, 1.055 M in THF, 2.61 mmol) −10 °C.[3] The reaction mixture was warmed to room temperature and stirred for *ca.* 1.5 hours, then cooled to −20 °C and the epoxide **6** added in THF (0.7 mL). The reaction was warmed to −10 °C and stirred for *ca.* 2 hours (t.l.c. control), quenched with saturated aqueous NH_4Cl solution and partitioned between water and dichloromethane. The combined organic phases were dried ($MgSO_4$), filtered and concentrated *in vacuo* to afford the crude product. Purification by flash chromatography (silica gel, eluting with 1:12, 1:8 diethyl ether/hexanes) afforded *enone* **13** (0.171 g, 64% yield) as a colorless oil: **R_f** = 0.72 (2:1 diethyl ether/hexanes); $[\alpha]_D^{20}$ +8.1 (*c* 0.5, $CHCl_3$); **^{1}H NMR** (500 MHz, $CDCl_3$) δ 6.83 (dt, *J* = 15.8, 7.4 Hz, 1H), 6.09 (d, *J* = 16.0 Hz, 1H), 5.68 (dt, *J* = 15.0, 7.4 Hz, 1H), 5.48 (dd, *J* = 15.4, 6.4 Hz, 1H), 4.20 (q, *J* = 6.1 Hz, 1H), 3.72 (app. dq, *J* = 7.1, 5.2 Hz, 1H), 3.59 (dd, A of ABX, J_{AB} = 9.6 Hz, J_{AX} = 5.1 Hz, 1H), 3.47 (dd, B of ABX, J_{AB} = 9.6 Hz, J_{BX} = 7.3 Hz, 1H), 2.91 (dddd, *J* = 6.3, 5.0, 3.8, 2.6 Hz, 1H), 2.74 (dd, M of AMX, J_{AM} = 4.8 Hz, J_{MX} = 4.1 Hz, 1H), 2.62 (dt, A of

[3] H. Ren, A. Krasovskiy and P. Knochel, *Org. Lett.*, 2004, **6**, 4215; A. Krasovskiy, B. F. Straub and P. Knochel, *Angew. Chem., Int. Ed.*, 2006, **45**, 159.

ABX$_2$, J_{AB} = 16.8 Hz, J_{AX} = 7.2 Hz, 1H), 2.58 (dt, B of ABX$_2$, J_{AB} = 16.8 Hz, J_{BX} = 7.2 Hz, 1H), 2.46 (dd, A of AMX, J_{AM} = 5.0 Hz, J_{AX} = 2.7 Hz, 1H), 2.42-2.33 (m, 3H), 2.19 (app. dt, B of ABXY, J_{AB} = 13.7 Hz, J_{BX} = J_{BY} = 6.8 Hz, 1H), 1.80 (app. d of quintets, A of ABX$_2$Y$_2$, J_{AB} = 15.9 Hz, J_{AX} = J_{AY} = 7.4 Hz, 1H), 1.77 (app. d of quintets, B of ABX$_2$Y$_2$, J_{AB} = 15.7 Hz, J_{BX} = J_{BY} = 7.4 Hz, 1H), 1.60 (dddd, A of ABXYZ, J_{AB} = 13.9 Hz, J_{AX} = 8.8 Hz, J_{AY} = 6.5 Hz, J_{AZ} = 5.0 Hz, 1H), 1.52 (app. dq, B of ABXY$_2$, J_{AB} = 14.2 Hz, J_{BX} = J_{BY} = 7.1 Hz, 1H), 1.12-0.99 (m, 21H), 0.95 (t, J = 8.0 Hz, 9H), 0.88 (s, 9H), 0.59 (q, J = 7.8 Hz, 6H), 0.03 (s, 3H), 0.02 (s, 3H); **^{13}C NMR** (125 MHz, CDCl$_3$) δ 199.92 (e), 144.24 (o), 134.98 (o), 132.42 (o), 127.24 (o), 72.83 (o), 72.66 (o), 66.90 (e), 52.15 (o), 47.01 (e), 41.90 (e), 39.22 (e), 37.21 (e), 32.07 (e), 25.97 (o), 20.64 (e), 18.31 (e), 18.15 (o), 12.09 (o), 7.03 (o), 5.12 (e), –4.05 (o), –4.67 (o); **IR** (Neat) 2948 (s), 2866 (s), 1700 (w), 1676 (m), 1633 (w), 1462 (m), 1251 (m), 1067 (s), 1004 (s), 974 (s), 882 (m), 775 (s) 742 (s) cm^{-1}; **HRMS** (ESI, [M+Na]$^+$) calcd for $C_{36}H_{72}NaO_5Si_3$ 691.4585, found 691.4590.

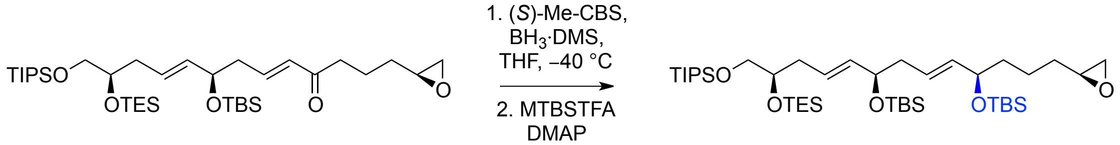

13 → **3**

((5*R*,6*E*,9*R*,10*E*,13*R*)-9-((*tert*-Butyldimethylsilyl)oxy)-16,16-diisopropyl-2,2,3,3,17-pentamethyl-5-(3-((*S*)-oxiran-2-yl)propyl)-13-((triethylsilyl)oxy)-4,15-dioxa-3,16-disilaoctadeca-6,10-diene (3). Borane dimethylsulfide complex (0.181 ml, 1.792 mmol) was added to the α,β-unsaturated ketone **13** (0.171 g, 0.256 mmol) and (*S*)-Me-CBS (0.256 ml, 1M in PhMe, 0.256 mmol) in THF (1.5 mL) at −40 °C. The reaction mixture was then stirred for *ca*. 16 hours (t.l.c. control) at this temperature, quenched with MeOH (0.1 mL) and partitioned between water and dichloromethane. The combined organic phases were dried (MgSO$_4$), filtered and concentrated *in vacuo* to afford the crude product. Purification by flash chromatography (silica gel, eluting with 1:3 to 1:2 diethyl ether/hexanes) afforded the *secondary alcohol* (0.170 g, 99% yield) as a colorless oil: **R$_f$** = 0.35 (1:1 diethyl ether/hexanes); $[\alpha]_D^{20}$ +3.4 (*c* 1.0, CHCl$_3$); **^{1}H NMR** (500 MHz, CDCl$_3$) δ 5.65 (ddd, J = 15.7, 7.2, 0.6 Hz, 1H), 5.61 (ddd, J = 15.7, 7.1, 0.9 Hz, 1H), 5.49 (ddt, J = 11.9, 7.1, 1.2 Hz, 1H), 5.46 (ddt, J = 13.8, 6.1, 1.3 Hz, 1H), 4.09 (q, J = 6.2 Hz, 1H), 4.05 (q, J = 6.4 Hz, 1H), 3.72 (app. dq, J = 6.5, 5.3 Hz, 1H), 3.59 (dd, A of ABX, J_{AB} = 9.7 Hz, J_{AX} = 5.3 Hz, 1H), 3.52 (dd, B of ABX, J_{AB} = 9.7 Hz, J_{BX} = 6.7 Hz, 1H), 2.91 (app. tdd, J = 5.3, 4.0, 2.7 Hz, 1H), 2.74 (dd, M of AMX, J_{AM} = 5.0 Hz, J_{MX} = 4.0 Hz, 1H), 2.47 (dd, A of AMX, J_{AM} = 5.0 Hz, J_{AX} = 2.7 Hz, 1H), 2.33 (app. dt, A of

ABXY, J_{AB} = 13.6 Hz, $J_{AX} = J_{AY}$ = 6.7 Hz, 1H), 2.27-2.16 (m, 3H), 1.62-1.43 (m, 7H), 1.10-1.03 (m, 21H), 0.95 (t, J = 8.0 Hz, 9H), 0.88 (s, 9H), 0.59 (q, J = 7.9 Hz, 6H), 0.03 (s, 3H), 0.02 (s, 3H); **^{13}C NMR** (125 MHz, $CDCl_3$) δ 135.44 (o), 135.18 (o), 128.86 (o), 126.54 (o), 73.41 (o), 73.14 (o), 73.05 (o), 66.94 (e), 52.35 (o), 47.21 (e), 41.54 (e), 37.14 (e), 36.96 (e), 32.50 (e), 26.04 (o), 22.10 (e), 18.37 (e), 18.16 (o), 12.10 (o), 7.04 (o), 5.10 (e), –4.09 (o), –4.58 (o); **IR** (Neat) 3441 (br, w), 2929 (s), 2866 (s), 1462 (m), 1251 (m), 1113 (s), 1097 (s), 1067 (s), 1005 (s), 971 (s), 882 (m), 835 (s), 775 (s), 744 (m) cm^{-1}; **HRMS** (ESI, $[M+Na]^+$) calcd for $C_{36}H_{74}NaO_5Si_3$ 693.4742, found 693.4745. The secondary alcohol (0.0713 g, 0.106 mmol) was dissolved in MeCN (0.15 mL) and treated with MTBSTFA (0.256 g, 1.062 mmol) and DMAP (0.065 g, 0.531 mmol) at room temperature. The reaction was allowed to stir for *ca.* 3 hours (t.l.c. control) and purified by flash chromatography (silica gel, eluting with 1:30 diethyl ether/hexanes) to afford the *tert-butyldimethylsilyl ether* **3** (0.083 g, 99% yield) as a colorless oil: **R$_f$** = 0.77 (1:2 diethyl ether/hexanes); $[\alpha]_D^{20}$ +7.9 (c 1.0, $CHCl_3$); **^{1}H NMR** (500 MHz, $CDCl_3$) δ 5.62 (dt, J = 14.9, 7.3 Hz, 1H), 5.55 (dt, J = 14.9, 7.3 Hz, 1H), 5.47 (dd, J = 15.4, 6.4 Hz, 1H), 5.41 (dd, J = 15.4, 6.6 Hz, 1H), 4.08 (q, J = 5.9 Hz, 1H), 4.05 (q, J = 6.0 Hz, 1H), 3.71 (app. dq, J = 6.6, 5.3 Hz, 1H), 3.58 (dd, A of ABX, J_{AB} = 9.6 Hz, J_{AX} = 5.2 Hz, 1H), 3.49 (dd, B of ABX, J_{AB} = 9.7 Hz, J_{BX} = 6.9 Hz, 1H), 2.91-2.87 (m, 1H), 2.74 (dd, M of AMX, J_{AM} = 4.9 Hz, J_{MX} = 4.1 Hz, 1H), 2.45 (dd, A of AMX, J_{AM} = 5.0 Hz, J_{AX} = 2.7 Hz, 1H), 2.35 (app. dt, A of ABXY, J_{AB} = 13.2 Hz, $J_{AX} = J_{AY}$ = 6.4 Hz, 1H), 2.24-2.14 (m, 3H), 1.58-1.40 (m, 6H), 1.12-1.00 (m, 21H), 0.95 (t, J = 8.0 Hz, 9H), 0.89 (s, 9H), 0.88 (s, 9H), 0.59 (q, J = 7.8 Hz, 6H), 0.04 (s, 3H), 0.03 (s, 3H), 0.02 (s, 3H), 0.02 (s, 3H); **^{13}C NMR** (125 MHz, $CDCl_3$) δ 135.70 (o), 135.56 (o), 126.66 (o), 126.34 (o), 73.60 (o), 73.54 (o), 73.04 (o), 67.04 (e), 52.45 (o), 47.26 (e), 41.82 (e), 38.31 (e), 37.40 (e), 32.62 (e), 26.07 (o), 21.85 (e), 18.37 (e), 18.16 (o), 12.08 (o), 7.05 (o), 5.11 (e), –3.96 (o), –4.07 (o),–4.59 (o), –4.63 (o); **IR** (Neat) 2947 (m), 2928 (s), 2864 (m), 1463 (m), 1251 (m), 1115 (s), 1095 (s), 1069 (s), 1005 (m), 882 (m), 836 (s), 775 (s), 745 (m) cm^{-1}; **HRMS** (ESI, $[M+Na]^+$) calcd for $C_{42}H_{88}O_5NaSi_4$ 807.5607, found 807.5618.

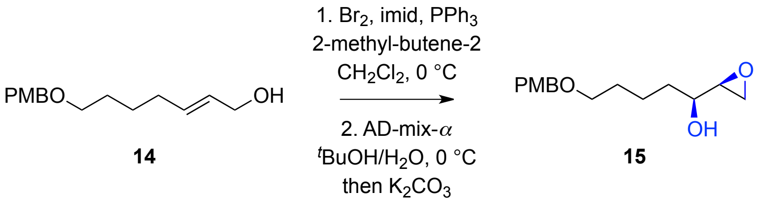

(*S*)-5-((4-Methoxybenzyl)oxy)-1-((*S*)-oxiran-2-yl)pentan-1-ol (15). Bromine (7.4 mL, 143 mmol) was carefully added dropwise to imidazole (15.02 g, 221 mmol) and triphenylphosphine (38.3 g, 146 mmol) in CH_2Cl_2 (210 mL) at 0 °C. The resultant yellow slurry was stirred for *ca.*

15 minutes and then the allylic alcohol **14**[4] (14.35 g, 57.3 mmol) and 2-methylbut-2-ene (20 mL, 57.3 mmol) in CH_2Cl_2 (50 mL) were added dropwise and stirred for *ca.* 15 minutes (t.l.c. control). The reaction was quenched with MeOH (1 mL), diluted with petroleum ether and filtered through silica gel eluting with 1:5 diethyl ether/petroleum ether and concentrated *in vacuo* to afford the crude *allylic bromide* that was used directly in the following step. An analytical sample was obtained by purification by flash chromatography (silica gel, eluting with 1:15 diethyl ether/hexanes) as a colorless oil: $\mathbf{R_f}$ = 0.65 (1:1 diethyl ether/hexanes); **^{1}H NMR** (500 MHz, $CDCl_3$) δ 7.27-7.25 (m, 2H), 6.90-6.87 (m, 2H), 5.76 (dt, *J* = 15.0, 6.6 Hz, 1H), 5.68 (dt, *J* = 15.0, 7.5 Hz, 1H), 4.43 (s, 2H), 3.94 (d, *J* = 7.3 Hz, 2H), 3.80 (s, 3H), 3.43 (t, *J* = 6.5 Hz, 2H), 2.07 (q, *J* = 7.1 Hz, 2H), 1.60 (dt, *J* = 14.5, 7.1 Hz, 2H), 1.46 (dt, *J* = 15.0, 7.5 Hz, 2H); **^{13}C NMR** (125 MHz, $CDCl_3$) δ 159.16 (e), 136.46 (o), 130.69 (e), 129.39 (o), 126.62 (o), 113.82 (o), 72.66 (e), 69.87 (e), 55.39 (o), 33.82 (e), 31.97 (e), 29.31 (e), 25.53 (e); **IR** (Neat) 2999 (w), 2933 (m), 2855 (m), 1660 (w), 1612 (m), 1586 (w), 1512 (s), 1463 (m), 1440 (w), 1246 (vs), 1098 (s), 1036 (s), 967 (m), 821 (m) cm^{-1}; **HRMS** (ESI, $[M+Na]^+$) calcd for $C_{15}H_{21}NaO_2{}^{79}Br$ 335.0623, found 335.0611, calcd for $C_{15}H_{21}O_2Na^{81}Br$ 337.0602, found 337.0618. The crude allylic bromide (17.95 g, 57.3 mmol) was dissolved in ^{t}BuOH/H_2O (560 mL; 1:1) and cooled with mechanical stirring to 0 °C. Potassium osmate(IV) dihydrate (0.211 g, 0.573 mmol), methanesulfonamide (5.45 g, 57.3 mmol), K_2CO_3 (23.76 g, 172 mmol), $K_3[Fe(CN)_6]$ (56.6 g, 172 mmol), $(DHQ)_2PHAL$ (0.893 g, 1.146 mmol) were added sequentially and the reaction stirred for *ca.* 6 hours (t.l.c. control). K_2CO_3 (39.6 g, 287 mmol) and H_2O (100 mL) were then added and the mixture was warmed to room temperature. The cyclisation was periodically checked over *ca.* 16 hours (by ^{1}H NMR), quenched with Na_2SO_3 (10 g, 73.5 mmol) and stirred for an additional hour. The resulting green solution was partitioned between water and ethyl acetate. The combined organic phases were washed with 2N aqueous NaOH solution, saturated aqueous NaCl solution, dried ($MgSO_4$), filtered and concentrated *in vacuo* to afford the crude product. Purification by flash chromatography (silica gel, eluting with 2.5:1 diethyl ether/petroleum ether) afforded the *hydroxy epoxide* **15** (11.5 g, 75% yield over 2 steps) as a colorless oil: $\mathbf{R_f}$ = 0.07 (1:1 Et_2O/hexanes); $[\alpha]_D^{20}$ +2.6 (*c* 1.0, $CHCl_3$); **^{1}H NMR** (500 MHz, $CDCl_3$) δ 7.26-7.24 (m, 2H), 6.88-6.85 (m, 2H), 4.42 (s, 2H), 3.79 (s, 3H), 3.45 (t, *J* = 6.4 Hz, 2H), 3.41 (app. quintet, *J* = 6.1 Hz, 1H), 2.96 (ddd, *J* = 5.0, 4.0, 2.9 Hz, 1H), 2.80 (dd, M of AMX, J_{AM} = 4.8 Hz, J_{MX} = 4.7 Hz, 1H), 2.69 (dd, A of AMX, J_{AM} = 4.9 Hz, J_{AX} = 2.8 Hz, 1H), 2.04 (br. s, 1H), 1.70-1.42 (m, 6H); **^{13}C NMR** (125 MHz, $CDCl_3$) δ 159.24 (e), 130.73 (e),

[4] M. E. Jung, J. A. Berliner, D. Angst, D. W. Yue, L. Koroniak, A. D. Watson and R. S. Li, *Org. Lett.*, 2005, **7**, 3933.

129.38 (o), 113.87 (o), 72.69 (e), 71.71 (o), 69.90 (e), 55.45 (o), 55.39 (o), 45.26 (e), 34.25 (e), 29.75 (e), 22.19 (e); **IR** (Neat) 3434 (br, w), 2936 (m), 2860 (m), 1612 (m), 1586 (w), 1512 (s), 1461 (w), 1245 (s), 1092 (s), 1032 (s), 819 (m) cm^{-1}; **HRMS** (ESI, $[M+Na]^+$) calcd for $C_{15}H_{22}NaO_4$ 289.1416, found 289.1403.

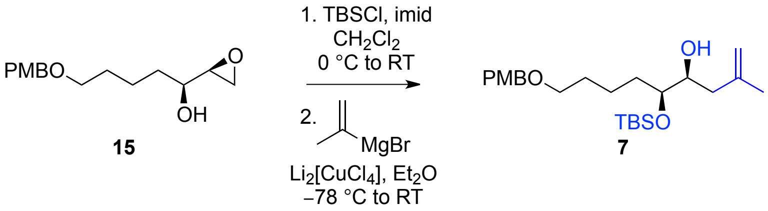

(4*S*,5*S*)-5-((*tert*-Butyldimethylsilyl)oxy)-9-((4-methoxybenzyl)oxy)-2-methylnon-1-en-4-ol (7). The secondary alcohol **15** (2.8 g, 10.51 mmol) was dissolved in DMF (30 mL) and treated with imidazole (2.147 g, 31.5 mmol) and TBSCl (3.17 g, 21.03 mmol) at 0 °C. The reaction mixture was warmed to room temperature and stirred for *ca.* 16 hours (t.l.c. control), quenched with saturated aqueous NH_4Cl solution and partitioned between water and diethyl ether. The combined organic phases were washed with water, saturated aqueous NaCl solution, dried ($MgSO_4$), filtered and concentrated *in vacuo* to afford the crude product. Purification by flash chromatography (silica gel, eluting with 1:7 to 1:6 diethyl ether/petroleum ether) afforded the *silyl ether* (3.22 g, 80% yield) as a colorless oil: $\mathbf{R_f}$ = 0.6 (1:1 diethyl ether/hexanes); $[\alpha]_D^{20}$ −4.2 (*c* 1.0, $CHCl_3$); **^{1}H NMR** (500 MHz, $CDCl_3$) δ 7.26-7.24 (m, 2H), 6.89-6.86 (m, 2H), 4.43 (s, 2H), 3.80 (s, 3H), 3.44 (t, J = 6.5 Hz, 2H), 3.25 (td, J = 7.0, 4.8 Hz, 1H), 2.90 (ddd, J = 6.7, 4.0, 2.8 Hz, 1H), 2.77 (app. t, M of AMX, $J_{AM} = J_{MX}$ = 4.5 Hz, 1H), 2.53 (dd, A of AMX, J_{AM} = 4.9 Hz, J_{AX} = 2.7 Hz, 1H), 1.65-1.35 (m, 6H), 0.91 (s, 9H), 0.11 (s, 3H), 0.06 (s, 3H); **^{13}C NMR** (125 MHz, $CDCl_3$) δ 159.22 (e), 130.80 (e), 129.33 (o), 113.85 (o), 74.68 (o), 72.67 (e), 69.97 (e), 56.04 (o), 55.37 (o), 45.01 (e), 34.67 (e), 29.90 (e), 25.99 (o), 22.17 (e), 18.30 (e), −4.24 (o), −4.87 (o); **IR** (Neat) 2929 (m), 2856 (m), 1613 (m), 1587 (w), 1513 (m), 1463 (m), 1246 (s), 1098 (s), 1036 (m), 939 (m), 835 (s), 776 (s) cm^{-1}; **HRMS** (ESI, $[M+Na]^+$) calcd for $C_{21}H_{36}NaO_4Si$ 403.2281, found 403.2296. Isopropenylmagnesium bromide (109 mL, 0.5 M in THF, 54.3 mmol) in Et_2O (120 mL) was cooled with stirring to −78 °C. Dilithium tetrachlorocuprate(II) (18.1 mL, 0.1 M in THF, 1.81 mmol) was added dropwise and the resultant orange suspension was stirred for *ca.* 10 min, before the addition of the terminal epoxide (6.89 g, 18.11 mmol) in Et_2O (25 mL). The reaction mixture was slowly warmed to room temperature and stirred for *ca.* 14 hours (t.l.c. control), quenched with saturated aqueous NH_4Cl/NH_4OH (4:1) and partitioned between water and diethyl ether. The combined organic phases were washed with water, saturated aqueous NaCl solution, dried ($MgSO_4$), filtered and

concentrated *in vacuo* to afford the crude product. Purification by flash chromatography (silica gel, eluting with 1:4 diethyl ether/petroleum ether) afforded the *homoallylic alcohol* **7** (7.6 g, 99% yield) as a colorless oil: The enantiomeric purity of **7** was determined by the formation of Mosher esters analysis (92% *ee*); **R**$_f$ = 0.58 (1:1 diethyl ether/hexanes); $[\alpha]_D^{20}$ −1.0 (*c* 0.5, $CHCl_3$); **^{1}H NMR** (500 MHz, $CDCl_3$) δ 7.26-7.25 (m, 2H), 6.88-6.86 (m, 2H), 4.84 (s, 1H), 4.77 (s, 1H), 4.43 (s, 2H), 3.80 (s, 3H), 3.66 (dt, J = 8.3, 4.1 Hz, 1H), 3.58 (dt, J = 5.5, 3.4 Hz, 1H), 3.45 (dt, A of ABX_2, J_{AB} = 9.7 Hz, J_{AX} = 6.6 Hz, 1H), 3.43 (dt, B of ABX_2, J_{AB} = 9.7 Hz, J_{BX} = 6.6 Hz, 1H), 2.18 (dd, A of ABX, J_{AB} = 14.3 Hz, J_{AX} = 5.1 Hz, 1H), 2.16 (s, 1H), 2.14 (dd, B of ABX, J_{AB} = 14.0 Hz, J_{BX} = 8.6 Hz, 1H), 1.76 (s, 3H), 1.69-1.58 (m, 3H), 1.46-1.36 (m, 3H), 0.91 (s, 9H), 0.08 (s, 6H); **^{13}C NMR** (125 MHz, $CDCl_3$) δ 159.19 (e), 143.06 (e), 130.77 (e), 129.30 (o), 113.83 (o), 112.77 (e), 74.54 (o), 72.64 (e), 70.46 (o), 70.00 (e), 55.33 (o), 42.16 (e), 33.29 (e), 30.05 (e), 26.00 (o), 22.60 (o), 22.11 (e), 18.20 (e), −4.02 (o), −4.43 (o); **IR** (Neat) 3472 (br, w), 2930 (m), 2856 (m), 1647 (w), 1613 (w), 1587 (w), 1513 (s), 1462 (m), 1247 (s), 1096 (s), 1037 (s), 835 (s), 775 (s) cm^{-1}; **HRMS** (ESI, $[M+Na]^+$) calcd for $C_{24}H_{42}NaO_4Si$ 445.2750, found 445.2759.

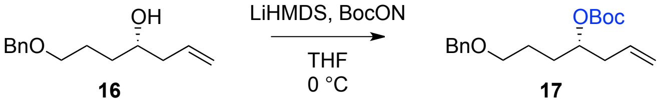

(*S*)-7-(Benzyloxy)hept-1-en-4-yl *tert*-butyl carbonate (17). LiHMDS (107 mL, 1 M in THF, 107 mmol) was added dropwise to the homoallylic alcohol **16**[5] (9.41 g, 42.72 mmol) in THF (140 mL) at 0 °C and stirred for *ca.* 10 minutes. BocON (26.35 g, 107 mmol) in THF (50 mL) was added and the mixture stirred for an additional *ca.* 15 minutes (t.l.c. control), quenched with saturated aqueous NH_4Cl solution and partitioned between water and diethyl ether. The organic phases were combined, dried ($MgSO_4$), filtered and concentrated *in vacuo* to afford the crude product. Purification by flash chromatography (silica gel, eluting with 1:20, 1:11, 1:10 diethyl ether/petroleum ether) afforded the *carbonate* **17** (13.0 g, 40.58 mmol, 95% yield) as a colorless oil: **R**$_f$ = 0.42 (1:3 diethyl ether/hexanes); $[\alpha]_D^{20}$ –15.6 (*c* 1.0, $CHCl_3$); **^{1}H NMR** (500 MHz, $CDCl_3$) δ 7.36-7.32 (m, 4H), 7.29-7.26 (m, 1H), 5.78 (ddt, J = 17.1, 10.1, 7.1 Hz, 1H), 5.11 (dd, J = 17.3, 1.7 Hz, 1H), 5.08 (dd, J = 11.6, 1.2 Hz, 1H), 4.72 (tt, J = 7.3, 5.5 Hz, 1H), 4.50 (s, 2H), 3.50-3.44 (m, 2H), 2.36 (t, J = 6.7 Hz, 2H), 1.74-1.62 (m, 4H), 1.48 (s, 9H); **^{13}C NMR** (125 MHz, $CDCl_3$) δ 153.46 (e), 138.57 (e), 133.57 (o), 128.44 (o), 127.68 (o), 127.60 (o), 117.95 (e), 81.77 (e), 76.34 (o), 72.93 (e), 69.95 (e), 38.90 (e), 30.44 (e), 27.88 (o), 25.71 (e); **IR**

[5] G. K. Packard, Y. Q. Hu, A. Vescovi and S. D. Rychnovsky, *Angew. Chem., Int. Ed.*, 2004, **43**, 2822.

(Neat) 2980 (w), 2934 (w), 2856 (w), 1732 (vs), 1643 (w), 1455 (m), 1367 (m), 1274 (vs), 1253 (vs), 1162 (s), 1093 (s), 917 (m), 736 (m) cm^{-1}; **HRMS** (ESI, $[M+Na]^+$) calcd for $C_{19}H_{28}NaO_4$ 343.1885, found 343.1886.

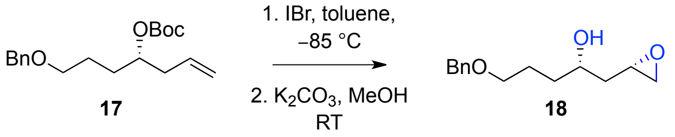

(*S*)-5-(Benzyloxy)-1-((*S*)-oxiran-2-yl)pentan-2-ol (18). Iodine bromide (5.54 mL, 1 M in CH_2Cl_2, 5.54 mmol) was added to a stirred solution of alkene **17** (2.985 g, 9.32 mmol) in toluene (28 mL) at −85 °C (Et_2O and dry ice bath) in the dark.[6] The reaction mixture was stirred for *ca.* 15 minutes (t.l.c. control), quenched with a mixture of 5% aqueous $NaHCO_3$ solution and 20% aqueous $Na_2S_2O_3$ solution (1:1), warmed to room temperature and partitioned between water and dichloromethane. The combined organic phases were dried (Na_2SO_4), filtered and concentrated *in vacuo* to afford the crude unstable *cyclic iodocarbonate*, which was used in the following step without purification. K_2CO_3 (3.86 g, 28.0 mmol) was then added to the crude cyclic iodocarbonate (~9.32 mmol) in MeOH (28 mL) at room temperature and the mixture stirred for *ca.* 4.5 hours (t.l.c. control). The reaction was quenched with saturated aqueous NH_4Cl solution and partitioned between water and dichloromethane. The combined organic phases were dried ($MgSO_4$), filtered and concentrated *in vacuo* to afford the crude product. Purification by flash chromatography (silica gel, eluting with 1:5 diethyl ether/hexanes) afforded the *hydroxy epoxide* **18** (1.779 g, 81% yield over 2 steps) as a colorless oil: $\mathbf{R_f}$ = 0.2 (1:1 ethyl acetate/hexanes); $[\alpha]_D^{20}$ −9.5 (*c* 1.0, $CHCl_3$); **^{1}H NMR** (500 MHz, $CDCl_3$) δ 7.36-7.32 (m, 3H), 7.30-7.26 (m, 2H), 4.51 (s, 2H), 3.90-3.82 (m, 1H), 3.53 (dt, A of ABX_2, J_{AB} = 9.5 Hz, J_{AX} = 5.9 Hz, 1H), 3.51 (dt, B of ABX_2, J_{AB} = 9.9 Hz, J_{BX} = 6.0 Hz, 1H), 3.08 (dtd, J = 7.0, 4.2, 2.8 Hz, 1H), 3.00 (br. s, 1H), 2.77 (app. t, M of AMX, J_{AM} = J_{MX} = 4.5 Hz, 1H), 2.49 (dd, A of AMX, J_{AM} = 5.0 Hz, J_{AX} = 2.8 Hz, 1H), 1.79-1.72 (m, 3H), 1.71-1.64 (m, 1H), 1.60 (dd, A of ABX, J_{AB} = 14.9 Hz, J_{AX} = 7.4 Hz, 1H), 1.54 (dd, B of ABX, J_{AB} = 14.0 Hz, J_{BX} = 7.1 Hz, 1H); **^{13}C NMR** (125 MHz, $CDCl_3$) δ 138.16 (e), 128.51 (o), 127.82 (o), 127.78 (o), 73.15 (e), 70.49 (e), 69.99 (o), 50.53 (o), 46.78 (e), 39.89 (e), 34.85 (e), 26.22 (e); **IR** (Neat) 3426 (br, w), 2921 (m), 2856 (m), 1496 (w), 1453 (m), 1362 (m), 1093 (s), 1027 (m), 925 (w), 829 (m), 737 (s) cm^{-1}; **HRMS** (CI, $[M+H]^+$) calcd for $C_{14}H_{21}O_3$ 237.1485, found 237.1488.

[6] J. J. W. Duan and A. B. Smith, *J. Org. Chem.*, 1993, **58**, 3703.

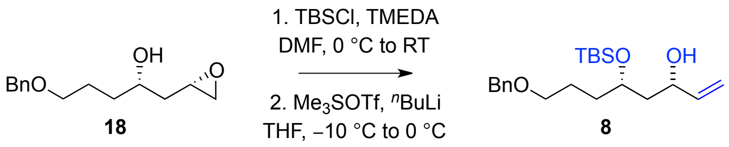

(3*S*,5*S*)-8-(Benzyloxy)-5-((*tert*-butyldimethylsilyl)oxy)oct-1-en-3-ol (8). The secondary alcohol **18** (1.779 g, 7.53 mmol) was dissolved in DMF (26 mL) at 0 °C and TMEDA (2.80 ml, 18.82 mmol) followed by TBSCl (2.269 g, 15.06 mmol) were added. The reaction mixture was warmed to room temperature and stirred for *ca.* 16 hours (t.l.c. control), quenched with saturated aqueous NH_4Cl solution and partitioned between water and diethyl ether. The combined organic phases were washed with water, saturated aqueous NaCl solution, dried ($MgSO_4$), filtered and concentrated *in vacuo* to afford the crude product. Purification by flash chromatography (silica gel, eluting with 1:13 to 1:10 diethyl ether/petroleum ether) afforded the *tert-butyldimethylsilyl ether* **32** (2.555 g, 97% yield) as a colorless oil: $\mathbf{R_f}$ = 0.79 (1:1 diethyl ether/hexanes); $[\alpha]_D^{20}$ +34.8 (*c* 0.25, $CHCl_3$); **^{1}H NMR** (500 MHz, $CDCl_3$) δ 7.37-7.32 (m, 4H), 7.31-7.26 (m, 1H), 4.51 (s, 2H), 3.91 (quintet, J = 5.5 Hz, 1H), 3.49 (t, J = 6.0 Hz, 2H), 3.04 (ddt, J = 6.8, 4.1, 2.7 Hz, 1H), 2.75 (app. t, M of AMX, $J_{AM} = J_{MX}$ = 4.5 Hz, 1H), 2.44 (dd, A of AMX, J_{AM} = 5.1 Hz, J_{AX} = 2.7 Hz, 1H), 1.76-1.59 (m, 6H), 0.91 (s, 9H), 0.07 (s, 3H), 0.07 (s, 3H); **^{13}C NMR** (125 MHz, $CDCl_3$) δ 138.65 (e), 128.39 (o), 127.67 (o), 127.55 (o), 72.89 (e), 70.45 (e), 70.19 (o), 49.44 (o), 46.83 (e), 40.21 (e), 33.74 (e), 25.91 (o), 25.82 (e), 18.09 (e), −4.46 (o), −4.50 (o); **IR** (Neat) 2950 (m), 2928 (m), 2854 (m), 1470 (w), 1456 (w), 1361 (w), 1253 (m), 1098 (m), 1071 (m), 834 (s), 774 (s), 735 (m) cm^{-1}; **HRMS** (ESI, $[M+Na]^+$) calcd for $C_{20}H_{34}NaO_3Si$ 373.2175, found 373.2162. ^{n}BuLi (14.3 mL, 2.5 M in hexanes, 35.7 mmol) was added slowly to a suspension of Me_3SOTf (8.24 g, 36.4 mmol) in THF (30 mL) at −10 °C and stirred for *ca.* 30 minutes. The epoxide (2.555 g, 7.29 mmol) in THF (5 mL) was added and the mixture was allowed to warm up to 0 °C and stirred for *ca*. 16 hours (t.l.c. control). The reaction was then quenched with saturated aqueous NH_4Cl solution and partitioned between water and dichloromethane. The combined organic phases were dried ($MgSO_4$), filtered and concentrated *in vacuo* to afford the crude product. Purification by flash chromatography (silica gel, eluting with 1:5, 1:4, 1:3 diethyl ether/petroleum ether) afforded the *allylic alcohol* **8** (2.45 g, 92% yield) as a colorless oil: $\mathbf{R_f}$ = 0.21 (1:3 diethyl ether/hexanes); $[\alpha]_D^{20}$ +20.8 (*c* 1.0, $CHCl_3$); **^{1}H NMR** (500 MHz, $CDCl_3$) δ 7.36-7.31 (m, 4H), 7.30-7.26 (m, 1H), 5.84 (ddd, J = 17.0, 10.7, 6.0 Hz, 1H), 5.26 (dt, J = 17.2, 1.3 Hz, 1H), 5.08 (dt, J = 10.5, 1.2 Hz, 1H), 4.50 (s, 2H), 4.26 (app. q, J = 6.0 Hz, 1H), 3.98 (quintet, J = 5.8 Hz, 1H), 3.47 (dt, A of ABX_2, J_{AB} = 9.1 Hz, J_{AX} = 5.8 Hz, 1H), 3.46 (dt, B of ABX_2, J_{AB} = 9.2 Hz, J_{BX} = 6.0 Hz, 1H), 3.15 (br. s, 1H),

1.69-1.58 (m, 4H), 1.66 (t, J = 5.4 Hz, 2H), 0.91 (s, 9H), 0.11 (s, 3H), 0.10 (s, 3H); **^{13}C NMR** (125 MHz, $CDCl_3$) δ 140.90 (o), 138.60 (e), 128.47 (o), 127.70 (o), 127.65 (o), 114.25 (e), 72.94 (e), 72.40 (o), 71.95 (o), 70.45 (e), 43.08 (e), 34.48 (e), 25.96 (o), 24.99 (e), 18.05 (e), −3.89 (o), −4.56 (o); **IR** (Neat) 3432 (br, w), 2947 (m), 2929 (m), 2856 (m), 1644 (w), 1472 (w), 1455 (w), 1361 (m), 1254 (m), 1060 (s), 920 (m), 836 (s), 775 (s), 735 (m) cm^{-1}; **HRMS** (ESI, $[M+Na]^+$) calcd for $C_{21}H_{36}O_3NaSi$ 387.2331, found 387.2323.

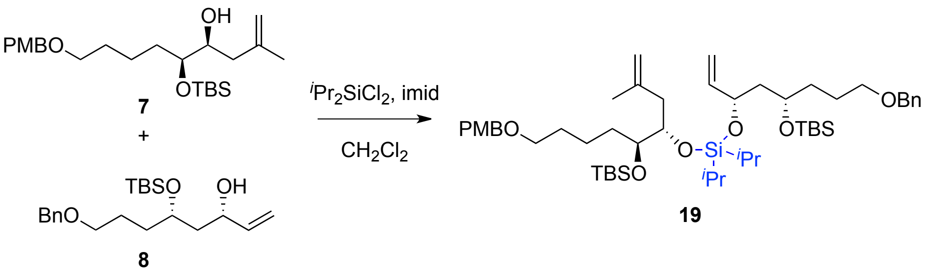

(5*S*,6*S*,10*S*,12*S*)-12-(3-(Benzyloxy)propyl)-8,8-diisopropyl-5-(4-((4-methoxybenzyl)oxy)butyl)-2,2,3,3,14,14,15,15-octamethyl-6-(2-methylallyl)-10-vinyl-4,7,9,13-tetraoxa-3,8,14-trisilahexadecane (19). *The procedure requires usage of Schlenk techniques and all operational steps should be performed under an atmosphere of argon.* A 50 mL round bottom Schlenk flask was charged with imidazole (0.340 g, 5.00 mmol) and CH_2Cl_2 (15 mL) and cooled with stirring to 0 °C. $^iPr_2SiCl_2$ (0.81 ml, 4.50 mmol) was added dropwise and after *ca.* 10 minutes a solution of **7** (0.423 g, 1 mmol) in CH_2Cl_2 (3 mL) was added *via* syringe pump over *ca.* 3 hours. The reaction mixture was then warmed to room temperature and stirred for *ca*. 16 hours, concentrated under reduced pressure using a dry ice trap, triturated with hexane (10 mL) and the solution carefully decanted from the precipitate using a syringe to a separate 100 ml Kugelrohr flask (3x). The hexane phases were concentrated under reduced pressure using a dry ice trap and the residue was heated at 75 °C *in vacuo* (1 Torr) for *ca.* 3 hours to remove the excess $^iPr_2SiCl_2$. The crude *monochlorosilane* was cooled with stirring to 0 °C in CH_2Cl_2 (2 mL) and imidazole (0.340 g, 5.00 mmol) added followed by the allylic alcohol **8** (0.286 g, 0.840 mmol) in CH_2Cl_2 (3 mL). The reaction mixture was warmed to room temperature and stirred for *ca*. 15 hours (t.l.c. control), quenched with saturated aqueous NH_4Cl solution and partitioned between water and diethyl ether. The combined organic phases were washed with water (2x), saturated aqueous NaCl solution, dried ($MgSO_4$), filtered and concentrated *in vacuo* to afford the crude product. Purification by flash chromatography (silica gel, eluting with 1:22 to 1:20 diethyl ether/petroleum ether) afforded the *mixed bis-alkoxysilane* **19** (0.634 g, 84% yield) as colorless oil: **R$_f$** = 0.53 (1:3 diethyl

ether/hexanes); $[\alpha]_D^{20}$ –4.9 (*c* 1.0, $CHCl_3$); **^{1}H NMR** (500 MHz, $CDCl_3$) δ 7.38-7.36 (m, 5H), 7.34-7.30 (m, 2H), 6.92 (d, J = 8.5 Hz, 2H), 5.85 (ddd, J = 17.2, 10.3, 7.0 Hz, 1H), 5.24 (d, J = 17.2 Hz, 1H), 5.13 (d, J = 10.2 Hz, 1H), 4.83 (s, 2H), 4.54 (s, 2H), 4.49 (d, A of AB, J_{AB} = 11.4 Hz, 1H), 4.50-4.46 (m, 1H), 4.47 (d, B of AB, J_{AB} = 11.5 Hz, 1H), 4.04 (ddd, J = 7.0, 3.8, 2.2 Hz, 1H), 3.82 (s, 3H), 3.82-3.77 (m, 2H), 3.54-3.47 (m, 4H), 2.43 (d, A of AB, J_{AB} = 13.2 Hz, 1H), 2.08 (dd, B of ABX, J_{AB} = 13.6 Hz, J_{BX} = 9.5 Hz, 1H), 1.93 (ddd, A of ABXY, J_{AB} = 13.4 Hz, J_{AX} = 8.6 Hz, J_{AY} = 4.8 Hz, 1H), 1.81 (s, 3H), 1.75-1.55 (m, 9H), 1.38-1.26 (m, 2H), 1.11-0.98 (m, 14H), 0.96 (s, 9H), 0.95 (s, 9H), 0.14 (s, 3H), 0.12 (s, 3H), 0.11 (s, 3H), 0.10 (s, 3H); **^{13}C NMR** (125 MHz, $CDCl_3$) δ 159.10 (e), 143.47 (e), 141.24 (o), 138.67 (e), 130.87 (e), 129.19 (o), 128.37 (o), 127.59 (o), 127.52 (o), 114.82 (e), 113.73 (o), 112.91 (e), 74.93 (o), 73.45 (o), 72.85 (e), 72.54 (e), 71.42 (o), 70.59 (e), 70.22 (e), 68.96 (o), 55.19 (o), 45.62 (e), 39.15 (e), 34.41 (e), 30.15 (e), 29.79 (e), 25.98 (o), 24.96 (e), 23.48 (e), 23.05 (o), 18.08 (e), 18.06 (e), 17.73 (o), 17.68 (o), 17.62 (o), 17.57 (o), 13.22 (o), 12.97 (o), –3.81 (o), –3.92 (o), –4.29 (o), –4.39 (o); **IR** (Neat) 2947 (m), 2929 (m), 2857 (m), 1646 (w), 1614 (w), 1585 (w), 1513 (m), 1463 (m), 1361 (m), 1248 (s), 1098 (s), 1039 (s), 1003 (m), 923 (m), 884 (m), 835 (s), 774 (s) cm^{-1}; **HRMS** (ESI, $[M+Na]^+$) calcd for $C_{51}H_{90}NaO_7Si_3$ 921.5892, found 921.5909.

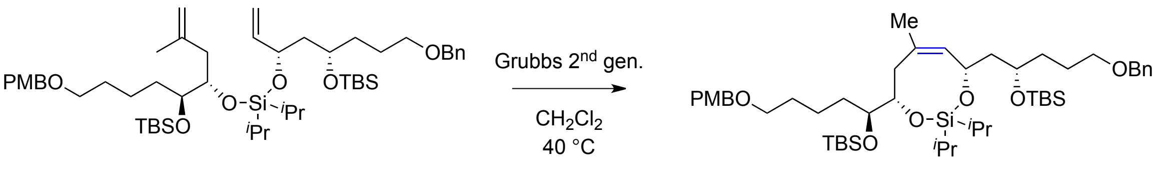

(4*S*,8*S*,*Z*)-8-((*S*)-5-(Benzyloxy)-2-((*tert*-butyldimethylsilyl)oxy)pentyl)-4-((*S*)-1-((*tert*-butyldimethylsilyl)oxy)-5-((4-methoxybenzyl)oxy)pentyl)-2,2-diisopropyl-6-methyl-5,8-dihydro-4*H*-1,3,2-dioxasilocine (20). Grubbs' 2nd generation catalyst (0.142 g, 0.167 mmol) in CH_2Cl_2 (4 mL) was added to a solution of diene **19** (1.0 g, 1.112 mmol) in CH_2Cl_2 (102 mL) and heated at reflux for *ca.* 24 hours. A second portion of Grubbs' 2nd generation catalyst (0.142 g, 0.167 mmol) in CH_2Cl_2 (4 mL) was added and the mixture was refluxed for a further *ca.* 48 hours (t.l.c. control), before it was cooled to room temperature and treated with DMSO (2.37 ml, 33.4 mmol) and stirred for *ca.* 16 hours. The reaction was then concentrated, filtered through a pad of silica gel, eluting with 1:5 diethyl ether/petroleum ether and concentrated *in vacuo* to afford the crude product. Purification by flash chromatography (silica gel, eluting with 1:20 diethyl ether/hexanes) afforded the *silaketal* **20** (0.94 g, 97% yield) as a colorless oil: $\mathbf{R_f}$ = 0.74 (1:2 diethyl ether/hexanes); $[\alpha]_D^{20}$ +1.4 (*c* 0.5, $CHCl_3$); **^{1}H NMR** (500 MHz, $CDCl_3$) δ 7.34-7.31 (m, 4H), 7.29-7.25 (m, 3H), 6.89-6.85 (m, 2H), 5.56 (dd, J = 5.9, 1.1 Hz, 1H), 4.50 (s, 2H),

4.44 (d, A of AB, J_{AB} = 11.7 Hz, 1H), 4.42 (d, B of AB, J_{AB} = 11.7 Hz, 1H), 4.40 (quintet, J = 4.9 Hz, 1H), 3.94 (app. septet, J = 3.9 Hz, 1H), 3.89 (ddd, J = 9.7, 3.7, 1.4 Hz, 1H), 3.80 (s, 3H), 3.62 (quintet, J = 3.9 Hz, 1H), 3.51-3.42 (m, 4H), 2.80 (dd, A of ABX, J_{AB} = 13.5 Hz, J_{AX}= 9.8 Hz, 1H), 1.98 (d, A of AB, J_{AB} = 12.8 Hz, 1H), 1.86 (ddd, B of ABXY, J_{AB} = 13.7 Hz, J_{BX} = 9.5 Hz, J_{BY} = 4.4 Hz, 1H), 1.76-1.68 (m, 2H), 1.72 (s, 3H), 1.67-1.55 (m, 5H), 1.54-1.41 (m, 2H), 1.35-1.25 (m, 2H), 1.07-1.00 (m, 1H), 0.99 (s, 3H), 0.98 (s, 3H), 0.98 (s, 3H), 0.97 (s, 3H), 0.94-0.85 (m, 1H), 0.89 (s, 9H), 0.88 (s, 9H), 0.06 (s, 3H), 0.05 (s, 3H), 0.05 (s, 3H), 0.05 (s, 3H); **^{13}C NMR** (125 MHz, $CDCl_3$) δ 159.20 (e), 138.83 (e), 136.44 (e), 131.12 (o), 130.93 (e), 129.34 (o), 128.45 (o), 127.70 (o), 127.57 (o), 113.86 (o), 75.74 (o), 75.56 (o), 72.88 (e), 72.69 (e), 70.89 (e), 70.32 (e), 69.65 (o), 68.77 (o), 55.40 (o), 45.82 (e), 35.25 (e), 32.73 (e), 31.02 (e), 30.15 (e), 26.09 (o), 26.05 (o), 25.69 (e), 25.65 (o), 23.12 (e), 18.23 (e), 18.17 (e), 17.95 (o), 17.92 (o), 17.75 (o), 17.73 (o), 13.58 (o), 13.19 (o), –4.09 (o), –4.19 (o), –4.36 (o), –4.40 (o); **IR** (Neat) 2929 (m), 2857 (m), 1729 (w), 1613 (w), 1587 (w), 1513 (m), 1462 (m), 1361 (w), 1248 (s), 1097 (s), 1038 (m), 1005 (m), 834 (s), 773 (s) 732 (m) cm^{-1}; **HRMS** (ESI, $[M+Na]^+$) calcd for $C_{49}H_{86}NaO_7Si_3$ 893.5579, found 893.5598.

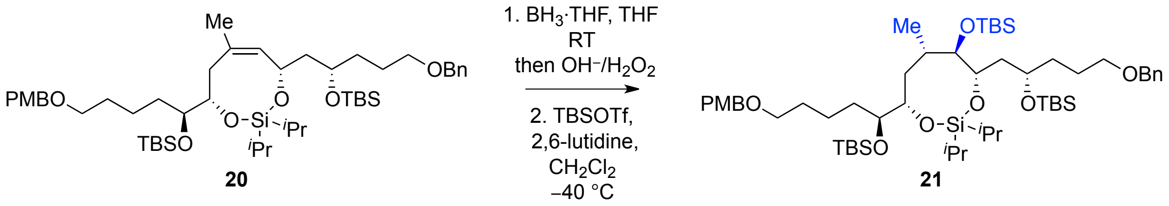

(4*S*,5*R*,6*S*,8*S*)-4-((*S*)-5-(Benzyloxy)-2-((*tert*-butyldimethylsilyl)oxy)pentyl)-5-((*tert*-butyldimethylsilyl)oxy)-8-((*S*)-1-((*tert*-butyldimethylsilyl)oxy)-5-((4-methoxybenzyl)oxy)pentyl)-2,2-diisopropyl-6-methyl-1,3,2-dioxasilocane (21). Borane tetrahydrofuran complex (3.0 mL, 1.0 M in THF, 3 mmol) was added to the silaketal **20** (0.516 g, 0.592 mmol) in THF (3 mL) at room temperature and stirred for *ca.* 2 hours (t.l.c. control). The reaction mixture was cooled to 0 °C and quenched with MeOH (0.1 mL), followed by the dropwise addition of a premixed cold solution of sodium hydroxide (4.9 mL, 14.80 mmol) and hydrogen peroxide (1.51 mL, 30% w/w, 14.80 mmol). The resulting mixture was vigorously stirred at room temperature for *ca.* 16 hours and then partitioned between water and ethyl acetate. The combined organic phases were washed with water, saturated aqueous NaCl solution, dried ($MgSO_4$), filtered and concentrated *in vacuo* to afford the *crude alcohol*, which was directly used in the next step. The crude alcohol (~0.592 mmol) was dissolved in CH_2Cl_2 (2 mL) and treated sequentially with 2,6-lutidine (0.69 mL, 5.92 mmol) and TBSOTf (0.82 mL, 3.55 mmol) at −40 °C for *ca.* 40 minutes (t.l.c. control). The reaction was then quenched with

saturated aqueous $NaHCO_3$ solution and partitioned between water and diethyl ether. The combined organic phases were washed with water, saturated aqueous NaCl solution, dried ($MgSO_4$), filtered and concentrated *in vacuo* to afford the crude product. Purification by flash chromatography (silica gel, eluting with 1:20 diethyl ether/hexanes) afforded the *tert-butyldimethyl ether* **21** (0.4254 g, 72% yield over 2 steps) as a colorless oil: **R_f** = 0.56 (1:5 diethyl ether/hexanes); $[\alpha]_D^{20}$ –5.5 (*c* 1.0, $CHCl_3$); **^{1}H NMR** (500 MHz, C_6D_6) δ 7.33-7.31 (m, 2H), 7.27-7.26 (m, 2H), 7.21-7.18 (m, 2H), 7.11-7.09 (m, 1H), 6.84-6.81 (m, 2H), 4.38 (d, A of AB, J_{AB} = 11.9 Hz, 1H), 4.35 (d, B of AB, J_{AB} = 10.9 Hz, 1H), 4.35 (s, 2H), 4.26 (app. qd, J = 6.4, 3.2 Hz, 1H), 4.21 (dd, J = 5.4, 3.1 Hz, 1H), 4.17 (dd, J = 9.8, 3.8 Hz, 1H), 4.08 (dt, J = 6.4, 5.3 Hz, 1H), 3.74 (dt, J = 8.1, 4.0 Hz, 1H), 3.44-3.35 (m, 4H), 3.30 (s, 3H), 2.19-2.08 (m, 2H), 2.11 (app. dt, A of ABXY, J_{AB} = 14.3 Hz, J_{AX} = J_{AY} = 7.2 Hz, 1H), 1.97 (ddd, B of ABXY, J_{AB} = 14.1 Hz, J_{BX} = 7.2 Hz, J_{BY} = 5.0 Hz, 1H), 1.93-1.84 (m, 4H), 1.77-1.63 (m, 4H), 1.57 (app. dt, B of ABXY, J_{AB} = 14.5 Hz, J_{BX} = J_{BY} = 9.9 Hz, 1H), 1.59-1.43 (m, 2H), 1.23 (d, J = 6.1 Hz, 3H), 1.23 (s, 3H), 1.21 (d, J = 7.3 Hz, 3H), 1.22 (s, 3H), 1.20-1.14 (m, 1H), 1.13-1.08 (m, 1H), 1.11 (d, J= 6.6 Hz, 3H), 1.05 (s, 9H), 1.04 (s, 9H), 1.00 (s, 9H), 0.25 (s, 3H), 0.23 (s, 3H), 0.22 (s, 3H), 0.19 (s, 3H), 0.10 (s, 3H), 0.10 (s, 3H); **^{13}C NMR** (125 MHz, C_6D_6) δ 159.64 (e), 139.53 (e), 131.56 (e), 129.27 (o), 128.51 (o), 127.67 (o), 127.56 (o), 114.05 (o), 77.95 (o), 76.21 (o), 75.49 (o), 72.96 (e), 72.79 (e), 71.92 (o), 70.78 (e), 70.18 (e), 70.10 (o), 54.80 (o), 43.71 (e), 37.08 (e), 34.81 (o), 33.90 (e), 31.39 (e), 30.63 (e), 26.35 (o), 26.32 (o), 26.22 (o), 25.44 (e), 23.50 (e), 18.46 (e), 18.44 (e), 18.42 (e), 18.12 (o), 18.08 (o), 17.79 (o), 17.72 (o), 16.43 (o), 14.10 (o), 13.78 (o), –3.53 (o), –3.64 (o), –3.68 (o), –4.05 (o), –4.22 (o); **IR** (Neat) 2951 (m), 2928 (m), 2856 (m), 1613 (w), 1587 (w), 1513 (m), 1463 (m), 1361 (w), 1248 (s), 1100 (s), 1075 (s), 1040 (s), 1004 (m), 884 (w), 832 (vs), 774 (vs), 733 (w) cm^{-1}; **HRMS** (ESI, $[M+Na]^+$) calcd for $C_{55}H_{102}NaO_8Si_4$ 1025.6550, found 1025.6556.

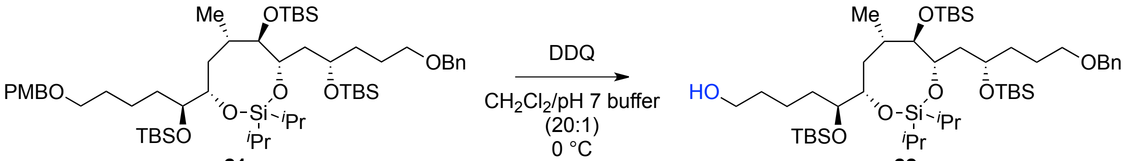

21 **22**

(4*S*,5*R*,6*S*,8*S*)-4-((*S*)-5-(Benzyloxy)-2-((*tert*-butyldimethylsilyl)oxy)pentyl)-5-((*tert*-butyldimethylsilyl)oxy)-8-((*S*)-1-((*tert*-butyldimethylsilyl)oxy)-5-(phenylsulfonyl)pentyl)-2,2-diisopropyl-6-methyl-1,3,2-dioxasilocane (22). DDQ (0.212 g, 0.934 mmol) was added to the PMB ether **21** (0.5857 g, 0.584 mmol) in CH_2Cl_2 (2.6 mL) and phosphate pH 7.0 buffer (0.13 mL) at 0 °C and stirred for *ca.* 2.5 hours (t.l.c. control). The reaction mixture was then

diluted with MeOH (1.5 mL) and treated portionwise with $NaBH_4$ (0.15 g, 3.96 mmol) and stirred for an additional *ca.* 3 hours (t.l.c. control). The reaction mixture was then quenched with saturated aqueous NH_4Cl solution and partitioned between water and ethyl acetate. The combined organic phases were washed with water, saturated aqueous NaCl solution, dried (Na_2SO_4), filtered and concentrated *in vacuo* to afford the crude product. Purification by flash chromatography (silica gel, eluting with 1:7 to 1:5 diethyl ether/petroleum ether) furnished the *primary alcohol* **22** (0.447 g, 87% yield) as a colorless oil: **R_f** = 0.14 (1:5 diethyl ether/hexanes); $[\alpha]_D^{20}$ –4.6 (*c* 0.5, $CHCl_3$); **^{1}H NMR** (500 MHz, C_6D_6) δ 7.31 (d, J = 7.3 Hz, 2H), 7.21-7.18 (m, 2H), 7.11-7.08 (m, 1H), 4.35 (s, 2H), 4.24 (app. qd, J = 6.4, 3.3 Hz, 1H), 4.20 (dd, J = 5.4, 3.2 Hz, 1H), 4.15 (dd, J = 9.9, 3.9 Hz, 1H), 4.08 (dt, J = 6.4, 5.4 Hz, 1H), 3.71 (dt, J = 8.2, 4.0 Hz, 1H), 3.41-3.38 (m, 4H), 2.18-2.06 (m, 2H), 2.09 (dd, A of ABX, J_{AB} = 15.6 Hz, J_{AX} = 6.8 Hz, 1H), 1.96 (ddd, A of ABXY, J_{AB} = 14.0 Hz, J_{AX} = 7.1 Hz, J_{AY} = 5.1 Hz, 1H), 1.92-1.82 (m, 4H), 1.68-1.62 (m, 1H), 1.58-1.40 (m, 5H), 1.55 (app. dt, B of ABXY, J_{AB} = 14.8 Hz, J_{BX} = J_{BY} = 10.0 Hz, 1H), 1.38-1.31 (m, 1H), 1.22 (d, J = 7.2 Hz, 3H), 1.21 (d, J = 7.4 Hz, 3H), 1.21 (s, 3H), 1.21 (d, J = 5.4 Hz, 3H), 1.19-1.14 (m, 1H), 1.12-1.07 (m, 1H), 1.10 (d, J = 6.5 Hz, 3H), 1.05 (s, 9H), 1.03 (s, 9H), 0.99 (s, 9H), 0.24 (s, 3H), 0.22 (s, 3H), 0.21 (s, 3H), 0.19 (s, 3H), 0.10 (s, 3H), 0.09 (s, 3H); **^{13}C NMR** (125 MHz, C_6D_6) δ 139.51 (e), 128.53 (o), 127.69 (o), 127.58 (o), 77.97 (o), 76.17 (o), 75.45 (o), 72.96 (e), 71.88 (o), 70.78 (e), 70.09 (o), 62.62 (e), 43.74 (e), 37.05 (e), 34.81 (o), 33.91 (e), 33.42 (e), 31.30 (e), 26.32 (o), 26.30 (o), 26.18 (o), 25.45 (e), 22.91 (e), 18.44 (e), 18.43 (e), 18.40 (e), 18.11 (o), 18.06 (o), 17.77 (o), 17.70 (o), 16.42 (o), 14.10 (o), 13.78 (o), –3.55 (o), –3.64 (o), –3.69 (o), –4.07 (o), –4.25 (o); **IR** (Neat) 3337 (br, w), 2947 (m), 2929 (m), 2857 (m), 1463 (m), 1361 (w), 1251 (m), 1099 (s), 1067 (s), 1004 (m), 884 (w), 833 (s), 773 (s), 733 (w) cm^{-1}; **HRMS** (ESI, $[M+Na]^+$) calcd for $C_{47}H_{94}NaO_7Si_4$ 905.5974, found 905.5984.

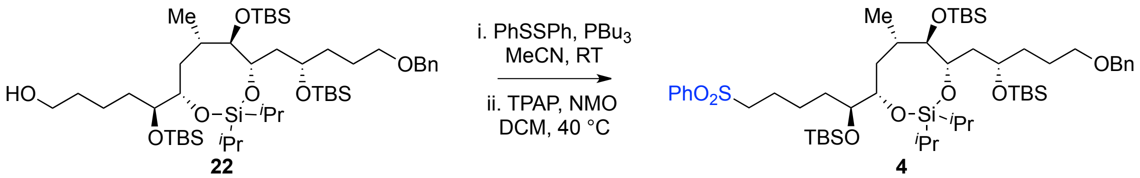

(4*S*,5*R*,6*S*,8*S*)-4-((*S*)-5-(Benzyloxy)-2-((*tert*-butyldimethylsilyl)oxy)pentyl)-5-((*tert*-butyldimethylsilyl)oxy)-8-((*S*)-1-((*tert*-butyldimethylsilyl)oxy)-5-(phenylsulfonyl)pentyl)-2,2-diisopropyl-6-methyl-1,3,2-dioxasilocane (4). Tributylphosphine (0.123 mL, 0.497 mmol) was added to a mixture of the primary alcohol **22** (0.3252 g, 0.368 mmol) and PhSSPh (0.108 g, 0.497 mmol) in MeCN (1.4 mL) and the resulting mixture stirred at room temperature for *ca.* 5

hours (t.l.c. control). TPAP (0.013 g, 0.037 mmol), NMO (0.690 g, 5.89 mmol) and CH_2Cl_2 (1.4 mL) were then added and the reaction was stirred at 40 °C for *ca.* 16 hours (t.l.c. control). The reaction mixture was quenched with saturated aqueous NH_4Cl solution and partitioned between water and ethyl acetate. The combined organic phases were washed with water, saturated aqueous NaCl solution, dried ($MgSO_4$), filtered and concentrated *in vacuo* to afford the crude product. Purification by flash chromatography (silica gel, eluting with 1:7 to 1:5 diethyl ether/hexanes) furnished *phenyl sulfone* **4** (0.283 g, 76% yield) as a colorless oil: $\mathbf{R_f}$ = 0.14 (1:5 diethyl ether/hexanes); $[\alpha]_D^{20}$ –3.6 (*c* 0.5, $CHCl_3$); **^{1}H NMR** (500 MHz, C_6D_6) δ 7.79-7.75 (m, 2H), 7.29 (d, J = 7.5 Hz, 2H), 7.20-7.17 (m, 2H), 7.11-7.08 (m, 1H), 7.01-6.93 (m, 3H), 4.35 (d, A of AB, J_{AB} = 12.4 Hz, 1H), 4.33 (d, B of AB, J_{AB} = 12.5 Hz, 1H), 4.20 (app. qd, J = 6.3, 3.4 Hz, 1H), 4.13 (d, J = 5.5, 3.2 Hz, 1H), 4.06-4.01 (m, 2H), 3.56 (dt, J = 7.2, 4.7 Hz, 1H), 3.38 (t, J = 6.1 Hz, 2H), 2.76 (dt, A of ABX_2, J_{AB} = 14.5 Hz, J_{AX} = 7.8 Hz, 1H), 2.73 (dt, B of ABX_2, J_{AB} = 14.7 Hz, J_{BX} = 7.8 Hz, 1H), 2.14-2.09 (m, 1H), 2.07 (app. dt, A of ABXY, J_{AB} = 13.9 Hz, J_{AX} = J_{AY} = 6.9 Hz, 1H), 1.99 (dd, A of ABX, J_{AB} = 14.9 Hz, J_{BX} = 7.0 Hz, 1H), 1.91 (ddd, B of ABXY, J_{AB} = 14.0 Hz, J_{BX} = 7.0 Hz, J_{BY} = 5.1 Hz, 1H), 1.88-1.78 (m, 3H), 1.68-1.59 (m, 4H), 1.42 (app. dt, B of ABXY, J_{AB} = 14.9 Hz, J_{BX} = J_{BY} = 10.1 Hz, 1H), 1.37-1.23 (m, 3H), 1.16 (d, J = 7.1 Hz, 3H), 1.15 (d, J = 7.2 Hz, 3H), 1.14 (d, J = 7.4 Hz, 3H), 1.13 (d, J = 7.5 Hz, 3H), 1.10-1.04 (m, 2H), 1.05 (d, J = 6.6 Hz, 3H), 1.02 (s, 9H), 1.00 (s, 9H), 0.94 (s, 9H), 0.21 (s, 3H), 0.19 (s, 3H), 0.19 (s, 3H), 0.16 (s, 3H), 0.04 (s, 3H), 0.01 (s, 3H); **^{13}C NMR** (125 MHz, C_6D_6) δ 140.64 (e), 139.52 (e), 133.08 (o), 129.13 (o), 128.52 (o), 128.34 (o), 127.67 (o), 127.57 (o), 77.98 (o), 75.68 (o), 75.30 (o), 72.96 (e), 71.80 (o), 70.77 (e), 70.08 (o), 56.36 (e), 43.75 (e), 36.82 (e), 34.71 (o), 33.92 (e), 30.69 (e), 26.32 (o), 26.31 (o), 26.14 (o), 25.40 (e), 25.35 (e), 23.32 (e), 18.43 (e), 18.33 (e), 18.08 (o), 18.03 (o), 17.75 (o), 17.65 (o), 16.40 (o), 14.03 (o), 13.68 (o), –3.57 (o), –3.63 (o), –3.69 (o), –4.06 (o), –4.27 (o), –4.30 (o); **IR** (Neat) 2951 (m), 2929 (m), 2857 (m), 1601 (w), 1463 (m), 1251 (m), 1149 (m), 1087 (s), 1004 (m), 885 (w), 834 (s), 774 (s), 733 (m) cm^{-1}; **HRMS** (ESI, $[M+Na]^+$) calcd for $C_{53}H_{98}NaO_8SSi_4$ 1029.5957, found 1029.5956.

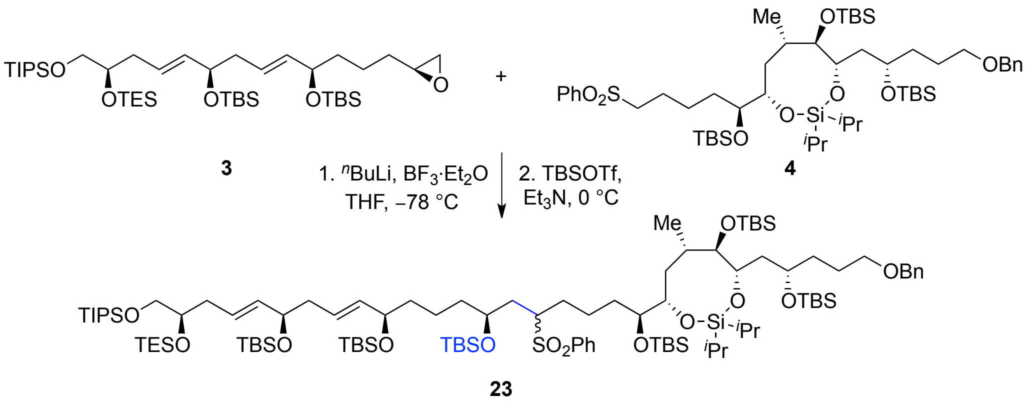

(4S,5R,6S,8S)-4-((S)-5-(Benzyloxy)-2-((*tert*-butyldimethylsilyl)oxy)pentyl)-5-((*tert*-butyldimethylsilyl)oxy)-2,2-diisopropyl-6-methyl-8-((5S,11S,15R,16E,19R,20E,23R)-11,15,19-tris((*tert*-butyldimethylsilyl)oxy)-26,26-diisopropyl-2,2,3,3,27-pentamethyl-9-(phenylsulfonyl)-23-((triethylsilyl)oxy)-4,25-dioxa-3,26-disilaoctacosa-16,20-dien-5-yl)-1,3,2-dioxasilocane (23). ^{n}BuLi (0.080 mL, 1.6 M in hexanes, 0.128 mmol) was added to the phenyl sulfone **4** (0.129 g, 0.128 mmol) in THF (0.3 mL) at –78 °C and stirred for *ca.* 15 minutes, which resulted in a yellow mixture. The epoxide **3** (0.050 g, 0.064 mmol) in THF (0.3 mL) was added, followed by $BF_3{\cdot}Et_2O$ (0.020 ml, 48% w/w, 0.077 mmol) and the mixture stirred for *ca.* 30 minutes (t.l.c. control). The reaction was then quenched with saturated aqueous NH_4Cl solution and partitioned between water and dichloromethane. The combined organic phases were dried ($MgSO_4$), filtered and concentrated *in vacuo* to afford the crude *hydroxy sulfone*, which was used in the next step without purification. The crude hydroxy sulfone (~0.064 mmol) was dissolved in CH_2Cl_2 (0.85 mL) and sequentially treated with triethylamine (0.053 mL, 0.384 mmol) and TBSOTf (0.073 mL, 0.320 mmol) at 0 °C and stirred for *ca.* 10 minutes (t.l.c. control). The reaction was then quenched with saturated aqueous $NaHCO_3$ solution and partitioned between water and diethyl ether. The combined organic phases were dried ($MgSO_4$), filtered and concentrated *in vacuo* to afford the crude product. Purification by flash chromatography (silica gel, eluting with 1:30, 1:22, 1:10, 1:5 diethyl ether/hexanes) afforded the *tert-butyldimethylsilyl ether* **23** (0.11 g, 90% yield) as a colorless oil and the recovered *phenyl sulfone* **4** (0.056 g, 87%): $\mathbf{R_f}$ = 0.42 (1:7 Et_2O/hexanes); $[\alpha]_D^{20}$ +5.9 (*c* 0.47, $CHCl_3$). *The presence of diastereoisomers complicated the NMR analysis. The ^1H NMR data of the predominant diastereoisomer is presented, while all observed ^{13}C peaks are provided;* **^{1}H NMR** (500 MHz, $CDCl_3$) δ 7.87-7.85 (m, 2H), 7.65-7.61 (m, 1H), 7.56-7.52 (m, 2H), 7.34-7.33 (m, 4H), 7.30-7.26 (m, 1H), 5.63 (dt, J = 15.0, 7.4 Hz, 1H), 5.55 (dd, J = 15.3, 7.7 Hz, 1H), 5.48 (ddd, J = 15.5, 6.4, 2.4 Hz, 1H), 5.41 (dd, J = 12.9, 6.6 Hz, 1H), 4.51 (d, A of

AB, J_{AB} = 12.5 Hz, 1H), 4.49 (d, B of AB, J_{AB} = 12.4 Hz, 1H), 4.08 (dt, J = 9.5, 5.8 Hz, 1H), 4.03-3.96 (m, 2H), 3.91-3.86 (m, 2H), 3.83-3.79 (m, 1H), 3.75-3.68 (m, 2H), 3.58 (dd, A of ABX, J_{AB} = 9.7 Hz, J_{AX} = 5.3 Hz, 1H), 3.50-3.46 (m, 3H), 3.49 (dd, B of ABX, J_{AB} = 9.6 Hz, J_{BX} = 6.9 Hz, 1H), 3.16-3.09 (m, 1H), 2.35 (app. dt, A of ABXY, J_{AB} = 13.2 Hz, J_{AX} = J_{AY} = 6.4 Hz, 1H), 2.27-2.13 (m, 2H), 1.94-1.53 (m, 11H), 1.46-1.17 (m, 13H), 1.10-0.99 (m, 17H), 1.06 (s, 12H), 1.05 (s, 6H), 0.95 (t, J = 8.0 Hz, 9H), 0.91-0.85 (m, 15H), 0.89 (s, 21H), 0.87 (s, 12H), 0.85 (s, 6H), 0.81 (s, 3H), 0.59 (q, J = 7.9 Hz, 6H), 0.07 (s, 3H), 0.06 (d, J = 7.1 Hz, 3H), 0.06 (s, 3H), 0.06 (d, J = 7.2 Hz, 3H), 0.04 (s, 9H), 0.03 (d, J = 8.3 Hz, 3H), 0.02 (s, 3H), 0.02 (d, J = 6.3 Hz, 3H), 0.01 (d, J = 6.5 Hz, 3H), –0.01 (s, 3H); **^{13}C NMR** (125 MHz, $CDCl_3$) δ 138.86 (e), 138.47 (e), 138.24 (e), 135.73 (o), 135.58 (o), 133.60 (o), 133.54 (o), 129.22 (o), 129.19 (o), 129.14 (o), 128.87 (o), 128.44 (o), 127.65 (o), 127.56 (o), 126.57 (o), 126.34 (o), 77.47 (o), 75.49 (o), 75.27 (o), 74.79 (o), 74.71 (o), 73.68 (o), 73.61 (o), 73.53 (o), 73.24 (o), 73.05 (o), 72.80 (e), 71.41 (o), 71.35 (o), 70.96 (e), 70.36 (o), 69.59 (o), 69.57 (o), 69.52 (o), 67.04 (e), 61.70 (o), 61.44 (o), 43.29 (e), 43.22 (e), 41.91 (e), 39.00 (e), 38.95 (e), 38.40 (e), 37.73 (e), 37.45 (e), 37.41 (e), 36.34 (e), 35.88 (e), 35.56 (e), 34.26 (o), 34.18 (o), 33.25 (e), 31.29 (e), 31.18 (e), 30.65 (e), 29.85 (e), 29.01 (e), 26.13 (o), 26.11 (o), 26.09 (o), 26.02 (o), 25.97 (o), 24.84 (e), 24.80 (e), 23.74 (e), 23.65 (e), 20.83 (e), 20.40 (e), 18.38 (e), 18.23 (e), 18.17 (o), 18.07 (e), 17.90 (o), 17.87 (o), 17.82 (o), 17.57 (o), 17.54 (o), 17.46 (o), 17.43 (o), 16.16 (o), 16.13 (o), 13.63 (o), 13.25 (o), 12.08 (o), 7.06 (o), 5.11 (e), –3.74 (o), –3.76 (o), –3.84 (o), –3.86 (o), –3.92 (o), –3.94 (o), –4.00 (o), –4.03 (o), –4.16 (o), –4.23 (o), –4.24 (o), –4.28 (o), –4.30 (o), –4.34 (o), –4.54 (o), –4.58 (o); **IR** (Neat) 2952 (m), 2929 (m), 2857 (m), 1472 (w), 1463 (w), 1361 (w), 1251 (m), 1084 (m), 1004 (m), 882 (w), 833 (s), 773 (s), 731 (m) cm^{-1}; **MS** (ESI, $[M+Na]^+$) calcd for $C_{101}H_{200}NaO_{13}SSi_9$ 1928.2525, found 1928.2481.

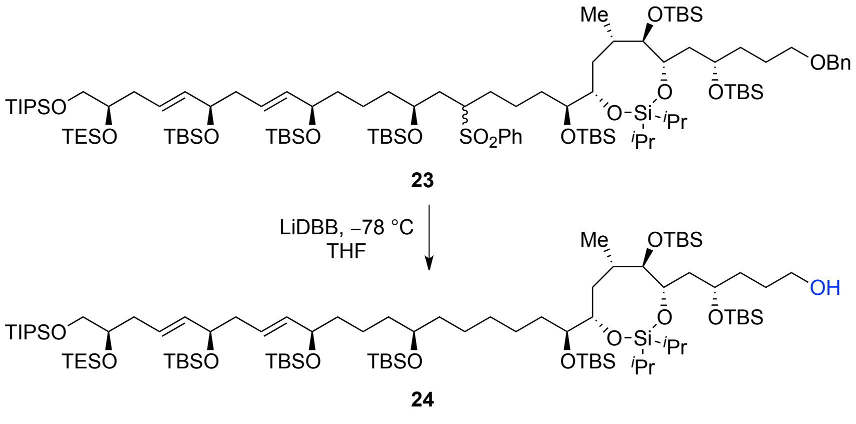

(*S*)-4-((*tert*-Butyldimethylsilyl)oxy)-5-((4*S*,5*R*,6*S*,8*S*)-5-((*tert*-butyldimethylsilyl)oxy)-2,2-diisopropyl-6-methyl-8-((5*S*,11*R*,15*R*,16*E*,19*R*,20*E*,23*R*)-11,15,19-tris((*tert*-

butyldimethylsilyl)oxy)-26,26-diisopropyl-2,2,3,3,27-pentamethyl-23-((triethylsilyl)oxy)-4,25-dioxa-3,26-disilaoctacosa-16,20-dien-5-yl)-1,3,2-dioxasilocan-4-yl)pentan-1-ol (24). Freshly prepared LiDBB (0.6 mL, 0.3 M in THF, 0.180 mmol) was added dropwise to the solution of phenyl sulfone **23** (0.0463 g, 0.024 mmol) in THF (0.2 mL) at –78 °C until the mixture maintained a deep green-blue color (t.l.c. control). The reaction mixture was quenched with saturated aqueous NH_4Cl solution and partitioned between water and dichloromethane. The combined organic phases were dried ($MgSO_4$), filtered and concentrated *in vacuo* to afford the crude product. Purification by flash chromatography (silica gel, eluting with 1:20, 1:10, 1:7 diethyl ether/hexanes) afforded the *primary alcohol* **24** (0.026 g, 64% yield) as a colorless oil: $\mathbf{R_f}$ = 0.21 (1:7 diethyl ether/hexanes); $[\alpha]_D^{20}$ +3.6 (*c* 0.07, $CHCl_3$); **^{1}H NMR** (500 MHz, $CDCl_3$) δ 5.62 (dt, *J* = 14.9, 7.4 Hz, 1H), 5.53 (dt, *J* = 14.8, 7.3 Hz, 1H), 5.47 (dd, *J* = 15.4, 6.9 Hz, 1H), 5.41 (dd, *J* = 15.2, 6.7 Hz, 1H), 4.10-4.05 (m, 2H), 4.01 (dt, *J* = 10.0, 5.0 Hz, 1H), 3.92 (dd, *J* = 6.3, 4.4 Hz, 1H), 3.91 (dd, *J* = 4.4, 2.6 Hz, 1H), 3.81 (dt, *J* = 7.4, 4.7 Hz, 1H), 3.71 (app. quintet, *J* = 5.6 Hz, 1H), 3.67-3.55 (m, 4H), 3.58 (dd, A of ABX, J_{AB} = 9.7 Hz, J_{AX} = 5.4 Hz, 1H), 3.49 (dd, B of ABX, J_{AB} = 9.6 Hz, J_{BX} = 6.9 Hz, 1H), 2.34 (app. dt, A of ABXY, J_{AB} = 13.2 Hz, J_{AX} = J_{AY} = 6.4 Hz, 1H), 2.24-2.13 (m, 4H), 1.95-1.87 (m, 2H), 1.83 (app. dt, A of ABXY, J_{AB} = 13.9 Hz, J_{AX} = J_{AY} = 6.9 Hz, 1H), 1.72 (ddd, B of ABXY, J_{AB} = 13.7 Hz, J_{BX} = 8.8 Hz, J_{BY} = 4.9 Hz, 1H), 1.67-1.58 (m, 5H), 1.52 (q, *J* = 6.2 Hz, 1H), 1.49-1.20 (m, 15H), 1.12-1.01 (m, 35H), 0.95 (t, *J* = 8.0 Hz, 9H), 0.92-0.87 (m, 57H), 0.59 (q, *J* = 7.9 Hz, 6H), 0.09 (s, 3H), 0.08 (s, 3H), 0.07 (s, 6H), 0.04 (s, 3H), 0.04-0.03 (m, 15H), 0.02 (s, 3H), 0.01 (s, 3H); **^{13}C NMR** (125 MHz, $CDCl_3$) δ 135.98 (o), 135.61 (o), 126.32 (o), 126.28 (o), 75.79 (o), 75.09 (o), 73.79 (o), 73.65 (o), 73.07 (o), 72.48 (o), 71.62 (o), 69.57 (o), 67.05 (e), 63.47 (e), 42.32 (e), 41.89 (e), 38.93 (e), 37.43 (e), 37.41 (e), 37.29 (e), 36.51 (e), 34.37 (o), 33.01 (e), 31.01 (e), 30.40 (e), 27.71 (e), 26.56 (e), 26.13 (o), 26.10 (o), 26.09 (o), 26.07 (o), 26.04 (o), 25.52 (e), 21.46 (e), 18.38 (e), 18.30 (e), 18.28 (e), 18.24 (e), 18.17 (o), 17.84 (o), 17.49 (o), 17.42 (o), 16.19 (o), 13.67 (o), 13.35 (o), 12.09 (o), 7.06 (o), 5.11 (e), –3.66 (o), –3.95 (o), –4.06 (o), –4.24 (o), –4.30 (o), –4.31 (o), –4.35 (o), –4.59 (o); **IR** (Neat) 3347 (br, w), 2929 (m), 2858 (m), 1472 (w), 1463 (w), 1361 (w), 1251 (m), 1067 (m), 1004 (m), 833 (s), 772 (s) cm^{-1}; **MS** (ESI, $[M+NH_4]^+$) calcd for $C_{88}H_{194}NO_{11}Si_9$ 1693.2570, found 1698.2575.

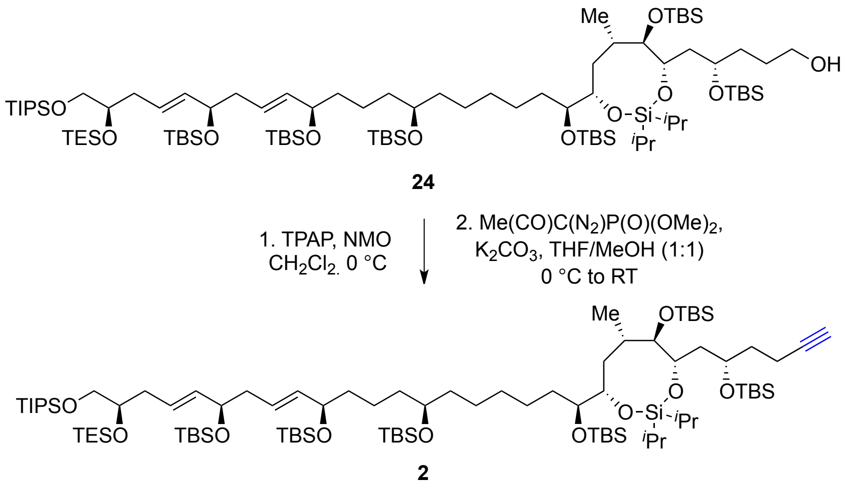

(4*S*,5*R*,6*S*,8*S*)-5-((*tert*-Butyldimethylsilyl)oxy)-4-((*S*)-2-((*tert*-butyldimethylsilyl)oxy)hex-5-yn-1-yl)-2,2-diisopropyl-6-methyl-8-((5*S*,11*R*,15*R*,16*E*,19*R*,20*E*,23*R*)-11,15,19-tris((*tert*-butyldimethylsilyl)oxy)-26,26-diisopropyl-2,2,3,3,27-pentamethyl-23-((triethylsilyl)oxy)-4,25-dioxa-3,26-disilaoctacosa-16,20-dien-5-yl)-1,3,2-dioxasilocane (2). NMO (0.011 g, 0.098 mmol) and TPAP (0.9 mg, 2.56 μmol) were added to the primary alcohol **24** (0.0164 g, 9.78 μmol) in CH_2Cl_2 (0.1 mL) at 0 °C and the mixture stirred for *ca.* 1 hour (t.l.c. control). The reaction mixture was then passed through a pad of silica gel, eluting with 1:10 diethyl ether/petroleum ether and concentrated *in vacuo* to furnish the crude *aldehyde*, which was used without purification. 1-Diazo-2-oxopropylphosphonate (0.019 g, 0.098 mmol) and K_2CO_3 (0.014 g, 0.098 mmol) were sequentially added to a stirred solution of the crude aldehyde in MeOH/THF (0.2 mL; 1:1) at 0 °C. The reaction mixture was warmed to room temperature and stirred for *ca.* 16 hours (t.l.c. control), passed through a pad of silica gel, eluting with diethyl ether and concentrated *in vacuo* to afford the crude product. Purification by flash chromatography (silica gel, eluting with 1:100 diethyl ether/hexanes) afforded *alkyne* **2** (0.0145 g, 89% yield over 2 steps) as a colorless oil: **R_f** = 0.89 (1:1 diethyl ether/hexanes); $[\alpha]_D^{20}$ –3.2 (*c* 0.32, $CHCl_3$); **^{1}H NMR** (500 MHz, $CDCl_3$) δ 5.62 (dt, *J* = 15.0, 7.4 Hz, 1H), 5.53 (dt, *J* = 14.9, 7.3 Hz, 1H), 5.47 (dd, *J* = 15.5, 6.3 Hz, 1H), 5.41 (dd, *J* = 15.3, 6.7 Hz, 1H), 4.06 (q, *J* = 6.3 Hz, 1H), 4.04-3.99 (m, 2H), 3.92-3.89 (m, 2H), 3.78 (dt, *J* = 8.0, 4.1 Hz, 1H), 3.70 (app. quintet, *J* = 5.7 Hz, 1H), 3.61-3.54 (m, 2H), 3.57 (dd, A of ABX, J_{AB} = 9.5 Hz, J_{AX} = 5.3 Hz, 1H), 3.48 (dd, B of ABX, J_{AB} = 9.6 Hz, J_{BX} = 6.9 Hz, 1H), 2.34 (app. dt, A of ABXY, J_{AB} = 13.0 Hz, J_{AX} = J_{AY} = 6.2 Hz, 1H), 2.28 (dd, A of ABX, J_{AB} = 11.2 Hz, J_{AX} = 3.0 Hz, 1H), 2.28-2.25 (m, 1H), 2.20 (quintet, *J* = 7.0 Hz, 1H), 2.17-2.13 (m, 2H), 1.92 (t, *J* = 2.6 Hz, 1H), 1.89 (dd, B of ABX, J_{AB} = 10.7 Hz, J_{BX} = 6.2 Hz, 1H), 1.82 (app. dt, A of ABXY, J_{AB} = 13.5 Hz, J_{AX} = J_{AY} = 6.9 Hz, 1H), 1.82-1.76 (m, 1H), 1.68-1.51 (m, 2H), 1.66 (ddd, B of ABXY, J_{AB} = 14.0 Hz, J_{BX} =

8.2 Hz, J_{BY} = 4.1 Hz, 1H), 1.53 (d, B of ABX, J_{AB} = 13.7 Hz, J_{BX} = 7.7 Hz, 1H), 1.47-1.38 (m, 6H), 1.34-1.18 (m, 10H), 1.10-0.98 (m, 35H), 0.94 (t, J = 7.9 Hz, 9H), 0.90-0.87 (m, 57H), 0.59 (q, J = 7.9 Hz, 6H), 0.08 (s, 3H), 0.08 (s, 3H), 0.07 (s, 3H), 0.06 (s, 3H), 0.04 (s, 3H), 0.03 (s, 3H), 0.03 (s, 6H), 0.02 (s, 6H), 0.01 (s, 3H), 0.01 (s, 3H); **^{13}C NMR** (125 MHz, $CDCl_3$) δ 135.95 (o), 135.59 (o), 126.31 (o), 126.26 (o), 85.08 (e), 77.34 (o), 75.70 (o), 75.08 (o), 73.78 (o), 73.63 (o), 73.03 (o), 72.46 (o), 71.44 (o), 68.47 (o), 68.33 (e), 67.00 (e), 42.96 (e), 41.87 (e), 38.89 (e), 37.39 (e), 37.26 (e), 36.34 (e), 35.61 (e), 34.45 (o), 30.86 (e), 30.38 (e), 26.57 (e), 26.15 (o), 26.09 (o), 26.08 (o), 26.05 (o), 26.02 (o), 25.51 (e), 24.26 (e), 21.47 (e), 19.93 (e), 18.38 (e), 18.29 (e), 18.23 (e), 18.16 (o), 17.81 (o), 17.50 (o), 17.42 (o), 16.09 (o), 13.87 (e), 13.64 (o), 13.31 (o), 12.04 (o), 7.07 (o), 5.07 (e), –3.65 (o), –3.84 (o), –3.94 (o), –3.96 (o), –4.06 (o), –4.25 (o), –4.33 (o), –4.45 (o), –4.60 (o); **IR** (Neat) 3315 (w), 2952 (m), 2929 (m), 2858 (m), 1463 (w), 1361 (w), 1252 (w), 1071 (m), 1004 (m), 834 (s), 773 (s) cm^{-1}; **MS** (ESI, [M+Na]$^{+}$) calcd for $C_{89}H_{188}NaO_{10}Si_9$ 1692.2018, found 1692.2082.

3. Global Deprotection and NMR Correlation Studies

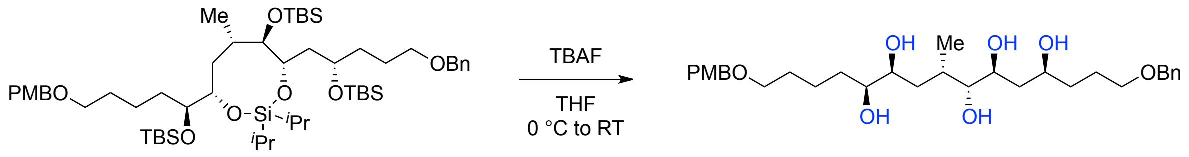

21

(4*S*,6*S*,7*R*,8*S*,10*S*,11*S*)-1-(Benzyloxy)-15-((4-methoxybenzyl)oxy)-8-methylpentadecane-4,6,7,10,11-pentaol. TBAF (0.66 ml, 1 M in THF, 660 μmol) was added to a solution of silaketal **21** (0.022 g, 0.022 mmol) in THF (0.1 mL) at 0 °C and the reaction mixture allowed to warm to room temperature and stirred for *ca.* 2 hours (t.l.c. control). The reaction was quenched with saturated aqueous NH_4Cl solution and partitioned between water and ethyl acetate. The combined organic phases were dried ($MgSO_4$), filtered and concentrated *in vacuo* to afford the crude product. Purification by flash chromatography (silica gel, eluting with 1:20 to 1:15 methanol/dichloromethane) provided *pentaol* (0.0083 g, 0.015 mmol, 69% yield) as clear oil. Analytically pure sample was obtained using preparative HPLC (Inertsil CN-3 column, 50:50 methanol/water, 0.8 mL/min, 25 °C, 210 nm, t_r(*pentaol*) = 26.0 min); **R_f** = 0.18 (1:20 methanol/dicholoromethane); $[\alpha]_D^{20}$ –19.4 (*c* 0.12, MeOH); **^{1}H NMR** (600 MHz, CD_3OD/pyridine-d_5 (2:1)) δ 7.28-7.25 (m, 4H), 7.23-7.18 (m, 3H), 6.86-6.84 (m, 2H), 4.41 (s, 2H), 4.35 (s, 2H), 3.96 (app. septet, J = 4.1 Hz, 1H), 3.85 (ddd, J = 9.5, 7.8, 2.5 Hz, 1H), 3.68 (s, 3H), 3.63 (ddd, J = 9.9, 4.4, 3.2 Hz, 1H), 3.46-3.43 (m, 2H), 3.43 (t, J = 5.8 Hz, 2H), 3.39 (t, J = 6.2 Hz, 2H), 2.38-2.32 (m, 1H), 2.08 (ddd, A of ABXY, J_{AB} = 14.3 Hz, J_{AX} = 4.2 Hz, J_{AY} = 2.6

Hz, 1H), 1.82-1.75 (m, 1H), 1.69-1.42 (m, 12H), 1.02 (d, J = 6.8 Hz, 3H); **^{13}C NMR** (125 MHz, CD_3OD/pyridine-d_5 (2:1)) δ 160.5, 140.0, 132.0, 130.3, 129.3, 128.7, 128.4, 114.7, 79.6, 75.9, 73.6, 73.4, 73.1, 72.9, 71.9, 71.5, 71.1, 55.7, 41.3, 38.9, 35.4, 33.9, 31.7, 30.9, 26.8, 23.8, 13.3; **IR** (Neat) 3399 (br, m), 2929 (s), 2871 (s), 1714 (w), 1610 (w), 1584 (w), 1512 (m), 1457 (m), 1362 (s), 1247 (vs), 1096 (s), 1030 (s), 988 (m), 880 (m), 821 (w), 737 (w) cm^{-1}; **HRMS** (ESI, $[M+H]^+$) calcd for $C_{31}H_{49}O_8$ 549.3422, found 549.3409.

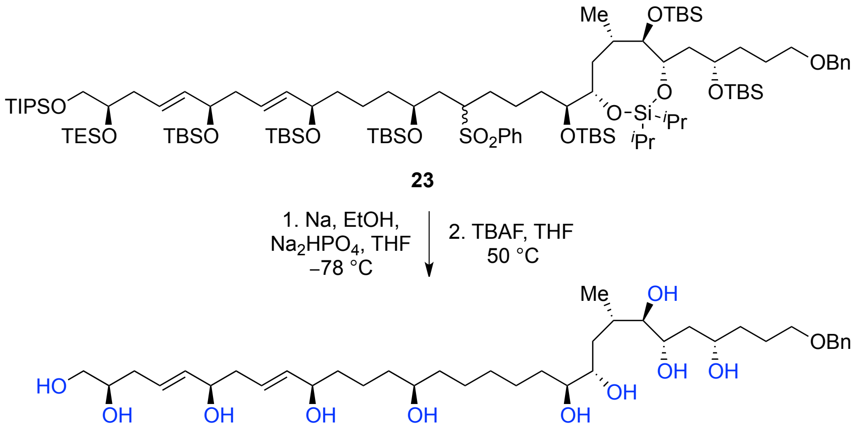

(2*R*,4*E*,6*R*,8*E*,10*R*,14*R*,20*S*,21*S*,23*S*,24*R*,25*S*,27*S*)-30-(Benzyloxy)-23-methyltriaconta-4,8-diene-1,2,6,10,14,20,21,24,25,27-decaol. Sodium (4.18 mg, 0.182 mmol) was added to a mixture of the phenyl sulfone **23** (0.0105 g, 5.50 μmol) and Na_2HPO_4 (5.47 mg, 0.039 mmol) in THF (0.324 ml) at –78 °C. The reaction was stirred for *ca.* 10 minutes, EtOH (0.021 ml, 0.363 mmol) added and the mixture stirred for *ca.* 16 hours (t.l.c. control). The reaction was then quenched with saturated aqueous NH_4Cl solution and partitioned between water and dichloromethane. The combined organic phases were dried (Na_2SO_4), filtered and concentrated *in vacuo* to furnish crude *desulfurization product*, which was used in the next step without purification. TBAF (0.550 ml, 1 M in THF, 550 μmol) was added to the desulfonated adduct (*ca*. 9.72 mg, 5.50 μmol) in THF (0.05 mL) and stirred at 50 °C for *ca.* 5 hours. The reaction mixture was then cooled to ambient temperature and stirred for a further *ca.* 14 hours (t.l.c. control). The mixture was diluted with MeOH (1 mL) and treated with $CaCO_3$ (0.165 g, 1650 μmol) and DOWEX 50WX8 resin (0.495 g) and stirred for *ca.* 5 hours, filtered through a pad of Celite, washing with MeOH and concentrated *in vacuo* to afford the crude product. The residue was dissolved in MeOH/H_2O (1:1), filtered through 0.2 μ porocity filter, concentrated and crude polyol was purified using preparative HPLC. Inertsil CN-3 column, 30:70 methanol/water, 0.61 mL/min, 25 °C, 210 nm, t_r(*polyol*) = 37.7 min to provide *polyol* (2.2 mg, 57% yield) as clear oil: $[\alpha]_D^{20}$ –14.8 (*c* 0.1, MeOH); **^{1}H NMR** (600 MHz, CD_3OD/pyridine-d_5 (2:1)) δ 7.28-7.25 (m, 4H), 7.21-7.18 (m, 1H), 5.77 (dt, J = 15.0, 7.4 Hz, 1H), 5.70 (dt, J = 14.9, 7.4 Hz, 1H), 5.61 (dd,

J = 15.4, 6.6 Hz, 1H), 5.56 (dd, J = 15.4, 6.9 Hz, 1H), 4.41 (s, 2H), 4.11 (app. q, J = 6.4 Hz, 1H), 4.05 (app. q, J = 6.4 Hz, 1H), 3.97 (app. septet, J = 4.1 Hz, 1H), 3.86 (ddd, J = 9.6, 7.6, 2.2 Hz, 1H), 3.74 (dtd, J = 11.8, 6.4, 5.4 Hz, 1H), 3.64 (ddd, J = 9.7, 4.4, 3.5 Hz, 1H), 3.59 (dd, A of ABX, J_{AB} = 11.0 Hz, J_{AX} = 4.7 Hz, 1H), 3.56-3.52 (m, 1H), 3.54 (dd, B of ABX, J_{AB} = 11.0 Hz, J_{BX} = 6.4 Hz, 1H), 3.47-3.42 (m, 2H), 3.43 (t, J = 6.5 Hz, 2H), 2.40-2.34 (m, 1H), 2.31 (app. dt, A of ABXY, J_{AB} = 13.6 Hz, J_{AX} = J_{AY} = 6.8 Hz, 1H), 2.29 (app. dt, B of ABXY, J_{AB} = 13.6 Hz, J_{BX} = J_{BY} = 6.8 Hz, 1H), 2.25 (app. dt, A of ABXY, J_{AB} = 13.7 Hz, J_{AX} = J_{AY} = 6.9 Hz, 1H), 2.22 (app. dt, B of ABXY, J_{AB} = 14.4 Hz, J_{BX} = J_{BY} = 7.2 Hz, 1H), 2.08 (ddd, A of ABXY, J_{AB} = 14.2 Hz, J_{AX} = 4.1 Hz, J_{AY} = 2.6 Hz, 1H), 1.82-1.75 (m, 1H), 1.70-1.31 (m, 18H), 1.26-1.17 (m, 4H), 1.02 (d, J = 6.7 Hz, 3H); **^{13}C NMR** (125 MHz, CD_3OD/pyridine-d$_5$ (2:1)) δ 140.0, 137.2, 136.5, 129.3 (2x), 128.7 (2x), 128.4, 128.3, 128.0, 79.6, 76.0, 73.6, 73.4, 73.1 (2x), 73.0, 72.9, 72.1, 71.9, 71.5, 66.9, 41.6, 41.4, 38.9, 38.6, 38.5 (2x), 37.7, 35.4, 34.1, 31.7, 30.9, 27.1, 26.8 (2x), 22.8, 13.2; **IR** (Neat) 3344 (br, s), 2928 (vs), 2859 (s), 1720 (w), 1668 (w), 1597 (m), 1562 (m), 1455 (s), 1381 (s), 1071 (s), 1035 (vs), 970 (s), 882 (m), 844 (m), 743 (s) cm^{-1}; **HRMS** (ESI, $[M+2H]^+$) calcd for $C_{38}H_{68}O_{11}$ 700.4756, found 700.4787.

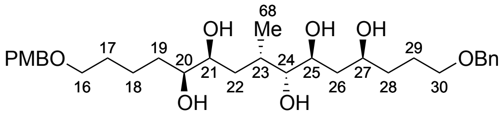

Table 2. Comparison of ^{1}H and ^{13}C NMR data of pentaol and natural polyol fragment of AM3 (CD_3OD-C_5D_5N (2:1)).[a]

Postn	Synthetic		Natural[b]		Difference	
	δ_C[b]	δ_H[c]	δ_C	δ_H	$\Delta\delta_C$	$\Delta\delta_H$
**19**	33.9	1.57, 1.49	34.1	1.56, 1.48	0.2	–0.01, –0.01
**20**	75.9	3.45	76.0	3.46	0.1	0.01
**21**	73.1	3.63	73.1	3.63	0	0
**22**	38.9	1.62, 1.58	38.9	1.63, 1.58	0	0.01, 0
**23**	31.7	2.34	31.6	2.32	–0.1	–0.02
**24**	79.6	3.43	79.7	3.45	0.1	0.02
**25**	72.9	3.85	72.8	3.86	–0.1	0.01
**26**	41.3	2.08, 1.63	41.4	2.08, 1.63	0.1	0, 0
**27**	71.9	3.96	71.8	3.95	–0.1	–0.01
**68**	13.3	1.02	13.2	1.01	–0.1	–0.01

[a] Chemical shifts calibrated using the signal of CD_2HOD and $^{13}CD_3OD$ at δ_H 3.30 and δ_C 49.0 respectively [b] 500 MHz [c] 600 MHz.

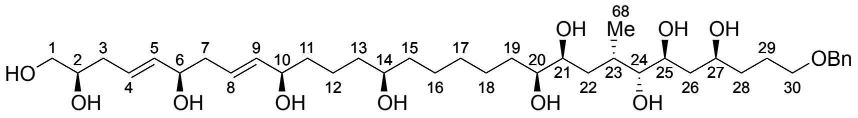

Table 1. Comparison of ^{1}H and ^{13}C NMR data of synthetic and natural polyol fragment of AM3 (CD_3OD-C_5D_5N (2:1)).[a]

Postn	Synthetic		Natural[b]		Difference	
	δ_C[b]	δ_H[c]	δ_C	δ_H	$\Delta\delta_C$	$\Delta\delta_H$
**1**	66.9	3.59, 3.54	66.9	3.58, 3.53	0	–0.01, –0.01
**2**	73.0	3.74	73.0	3.74	0	0
**3**	37.7	2.31, 2.22	37.7	2.31, 2.22	0	0, 0
**4**	128.3	5.76	128.5	5.77	0.2	0.01
**5**	136.5	5.61	136.5	5.61	0	0
**6**	73.1	4.11	73.1	4.11	0	0
**7**	41.6	2.29, 2.25	41.6	2.29, 2.25	0	0, 0
**8**	128.0	5.70	128.1	5.69	0.1	–0.01
**9**	137.2	5.56	137.2	5.56	0	0
**10**	73.4	4.05	73.4	4.06	0	0.01
**11**	38.6	1.57, 1.52	38.5	1.57, 1.52	–0.1	0, 0
**12**	22.8	1.60, 1.43	22.8	1.60, 1.43	0	0, 0
**13**	38.5	1.43	38.5	1.43	0	0
**14**	72.1	3.54	72.1	3.54	0	0
**15**	38.5	1.43	38.5	1.43	0	0
**16**	26.8	1.45, 1.33	26.8	1.45, 1.34	0	0, 0.01
**17**	30.9	1.32, 1.24	31.0	1.33, 1.25	0.1	0.01, 0.01
**18**	27.1	1.56, 1.38	27.1	1.56, 1.38	0	0, 0
**19**	34.1	1.56, 1.48	34.1	1.56, 1.48	0	0, 0
**20**	76.0	3.46	76.0	3.46	0	0
**21**	73.1	3.64	73.1	3.63	0	–0.01
**22**	38.9	1.63, 1.58	38.9	1.63, 1.58	0	0, 0
**23**	31.7	2.35	31.6	2.32	–0.1	–0.03
**24**	79.6	3.44	79.7	3.45	0.1	0.01
**25**	72.9	3.86	72.8	3.86	–0.1	0
**26**	41.4	2.08, 1.63	41.4	2.08, 1.63	0	0, 0
**27**	71.9	3.97	71.8	3.95	–0.1	–0.02
**68**	13.2	1.02	13.2	1.01	0	–0.01

[a] Chemical shifts calibrated using the signal of CD_2HOD and $^{13}CD_3OD$ at δ_H 3.30 and δ_C 49.0 respectively [b] 500 MHz [c] 600 MHz.

4. Copies of NMR Spectra

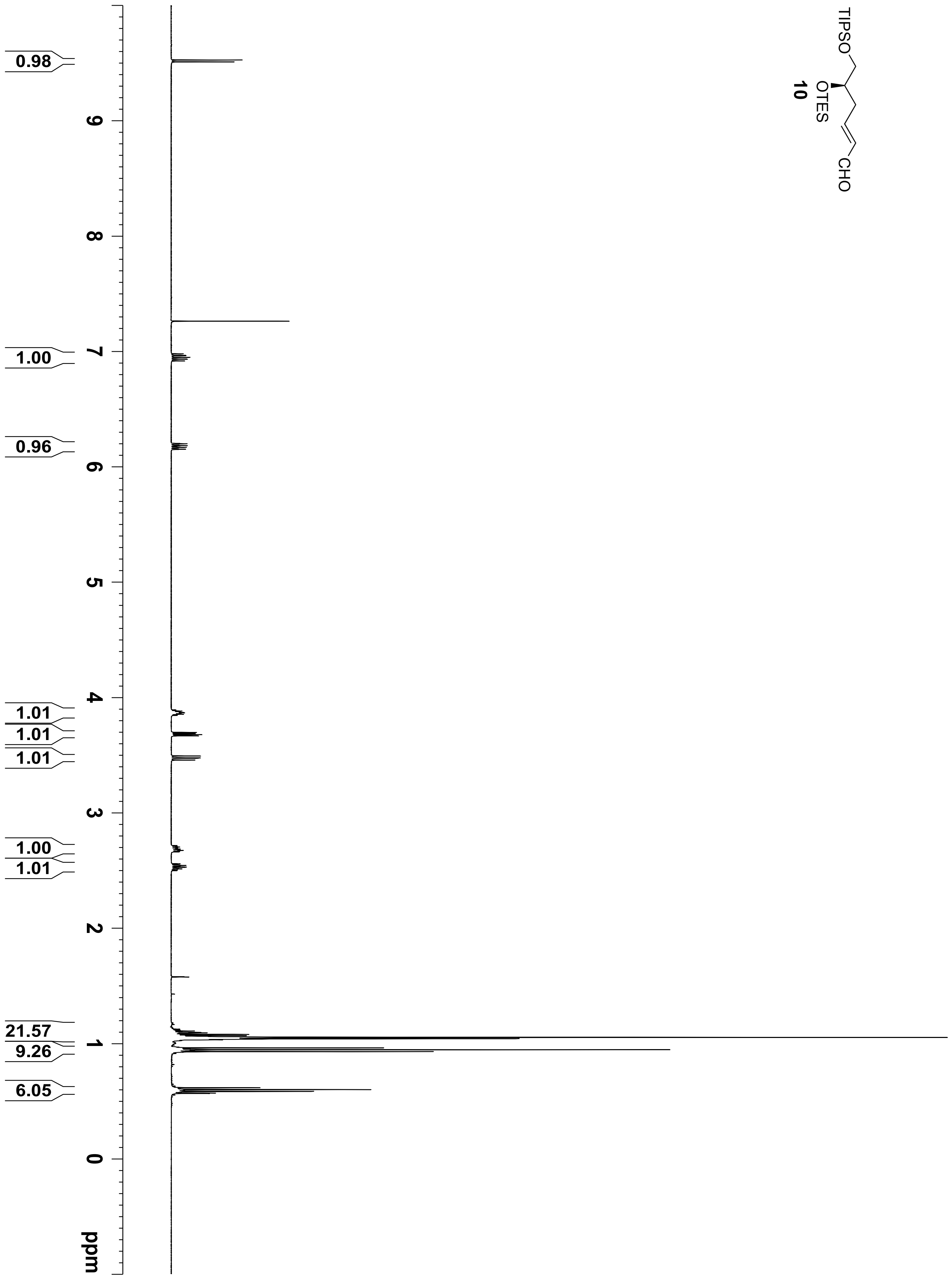

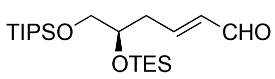
TIPSO
OTES
CHO

10

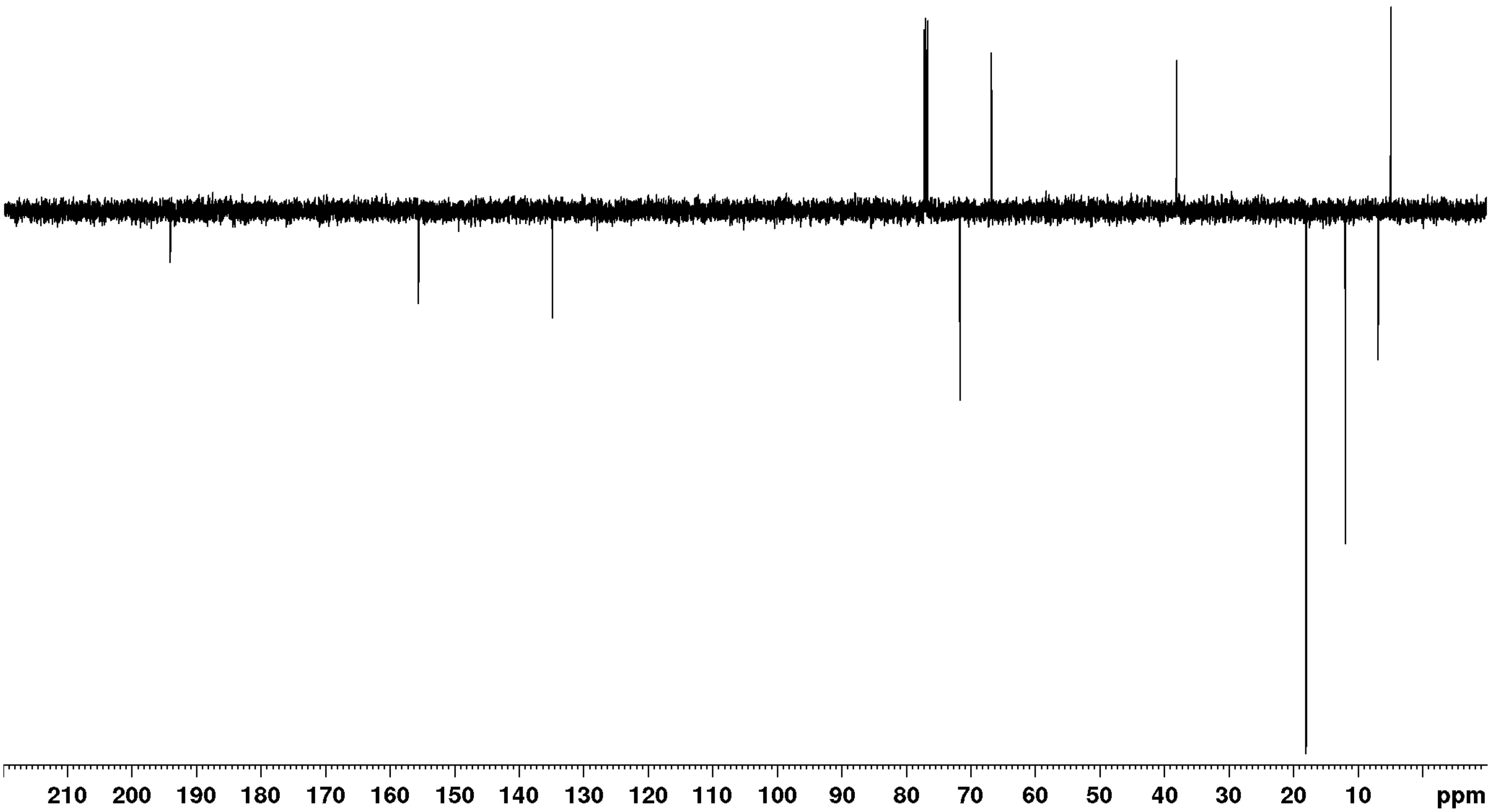
210 200 190 180 170 160 150 140 130 120 110 100 90 80 70 60 50 40 30 20 10 ppm

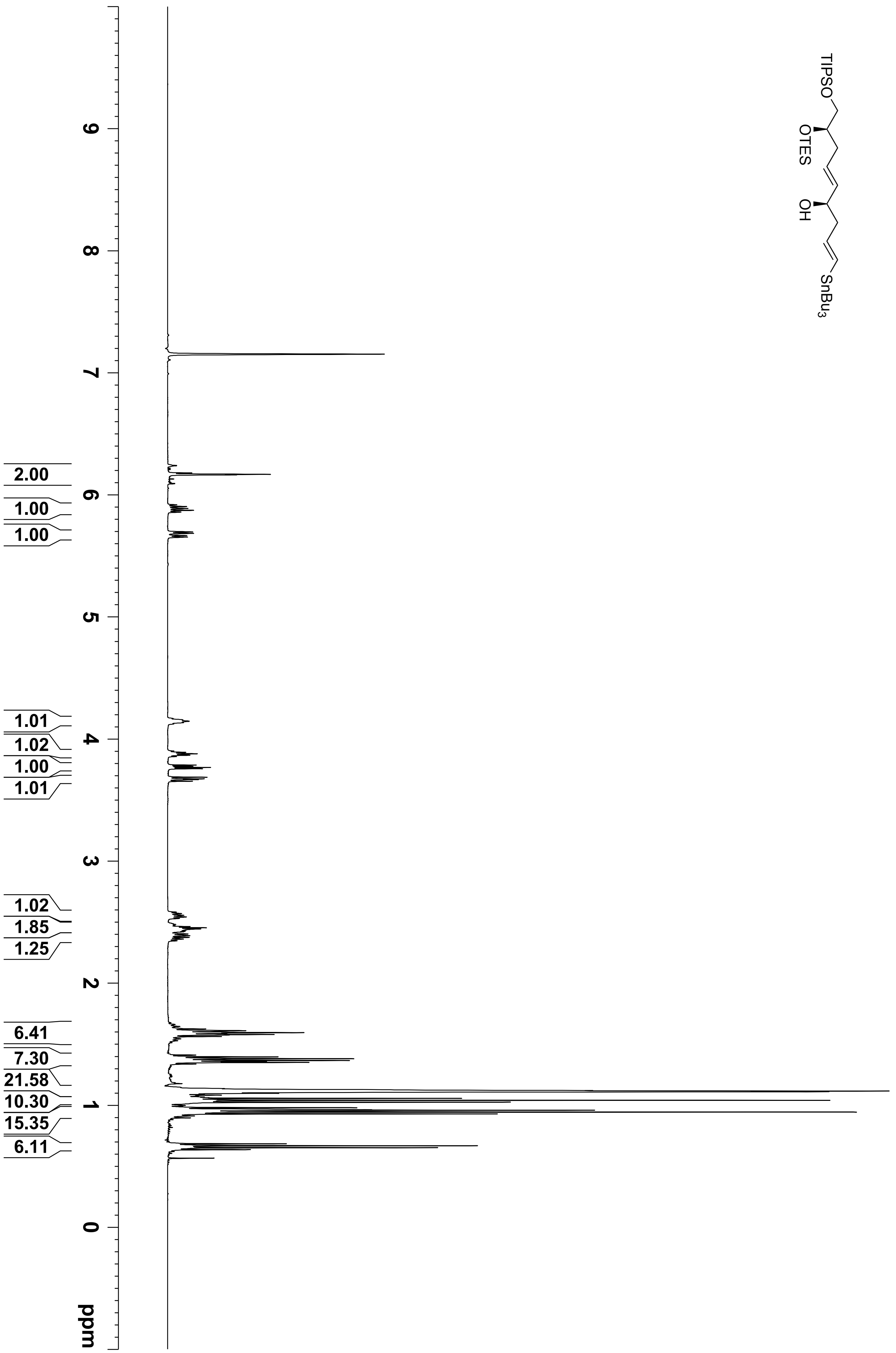
TIPSO
OTES
OH
$SnBu_3$
2.00
1.00
1.00
1.01
1.02
1.00
1.01
1.02
1.85
1.25
6.41
7.30
21.58
10.30
15.35
6.11
9
8
7
6
5
4
3
2
1
0
ppm

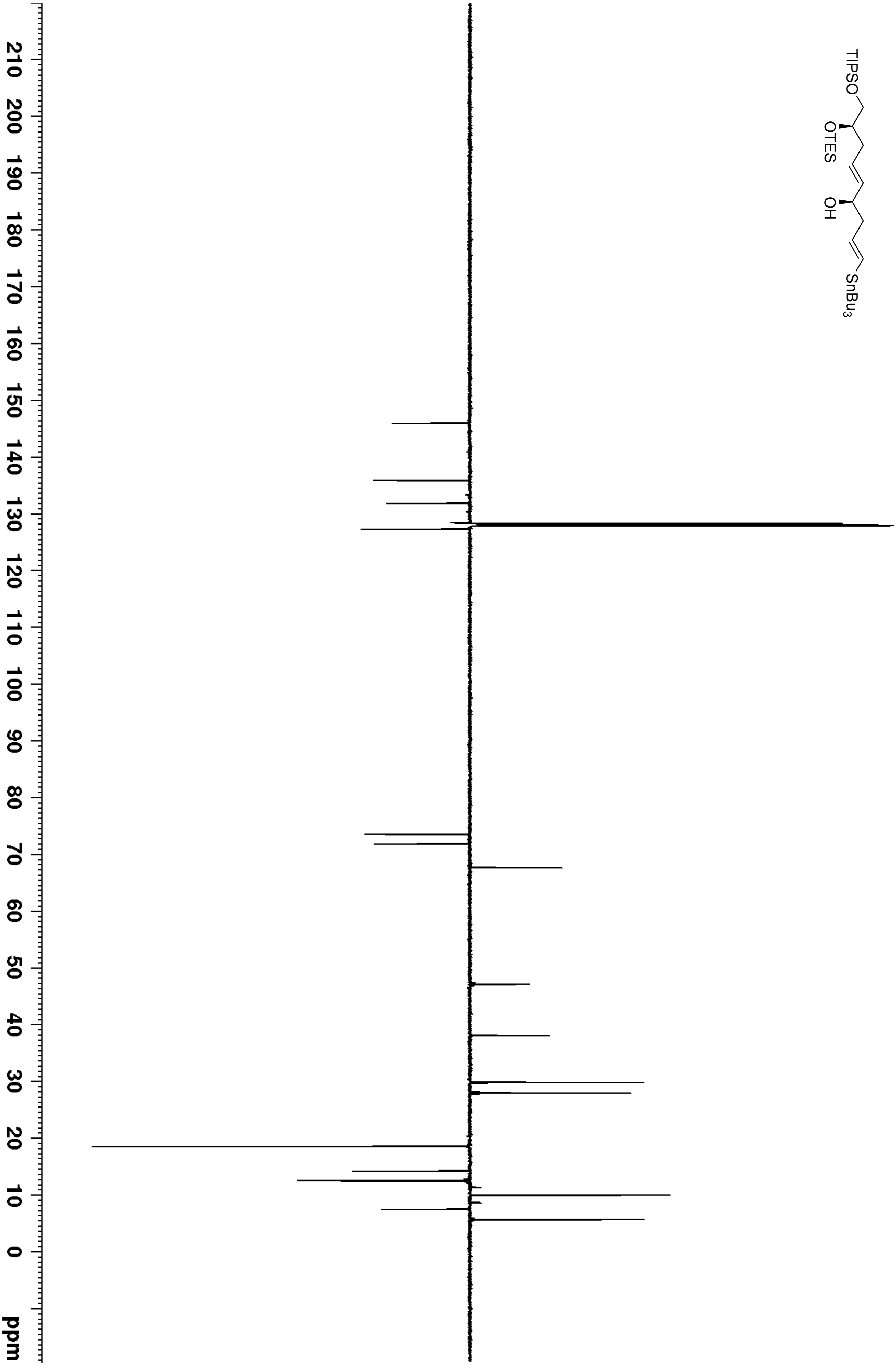
TIPSO
OTES
OH
SnBu3
210 200 190 180 170 160 150 140 130 120 110 100 90 80 70 60 50 40 30 20 10 0 ppm

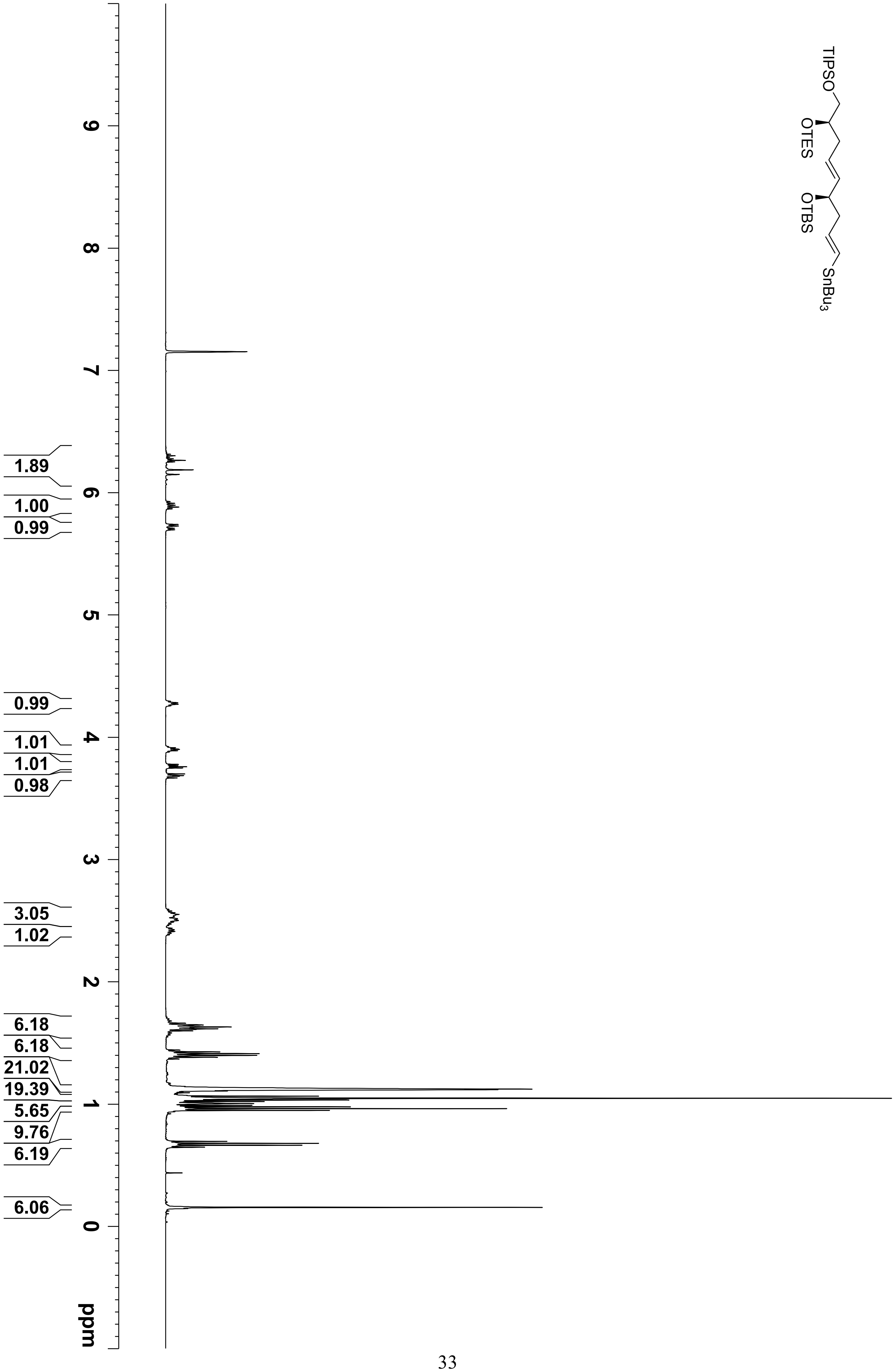
TIPSO
OTES
OTBS
SnBu3
1.89
1.00
0.99
0.99
1.01
1.01
0.98
3.05
1.02
6.18
6.18
21.02
19.39
5.65
9.76
6.19
6.06
9
8
7
6
5
4
3
2
1
0
ppm

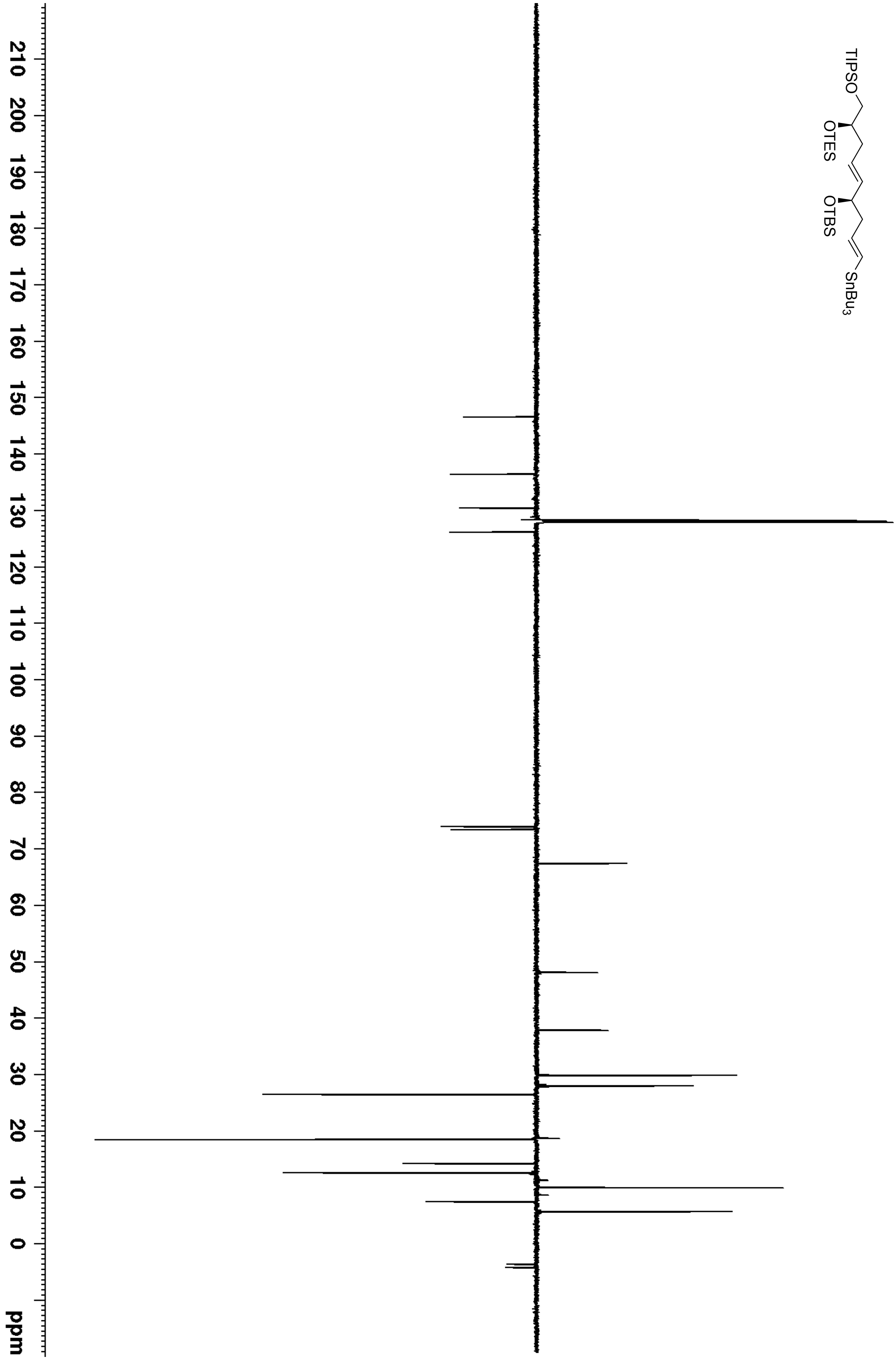
TIPSO
OTES
OTBS
SnBu3
210 200 190 180 170 160 150 140 130 120 110 100 90 80 70 60 50 40 30 20 10 0 ppm

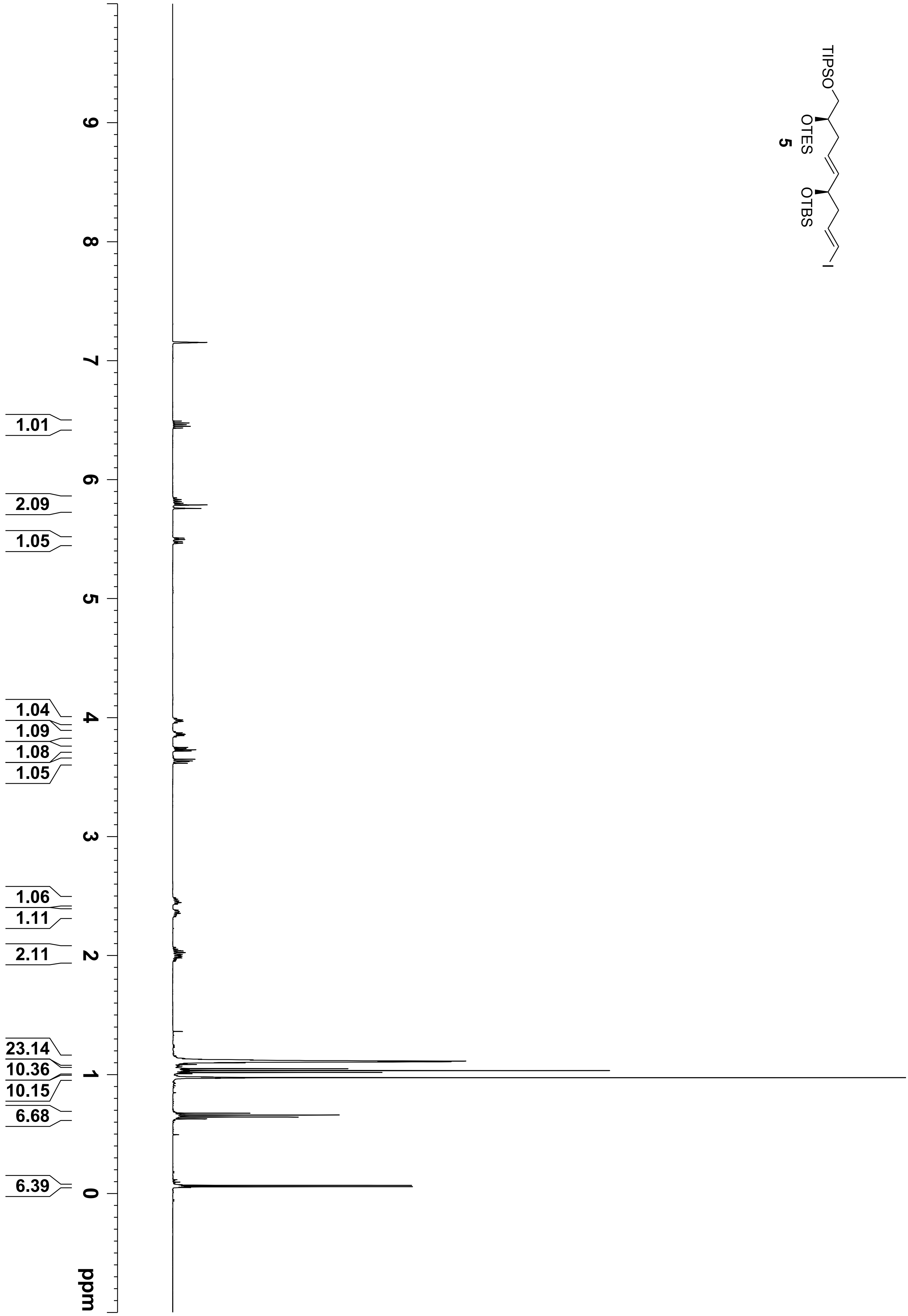
TIPSO
OTES
OTBS
I
5
1.01
2.09
1.05
1.04
1.09
1.08
1.05
1.06
1.11
2.11
23.14
10.36
10.15
6.68
6.39
9
8
7
6
5
4
3
2
1
0
ppm

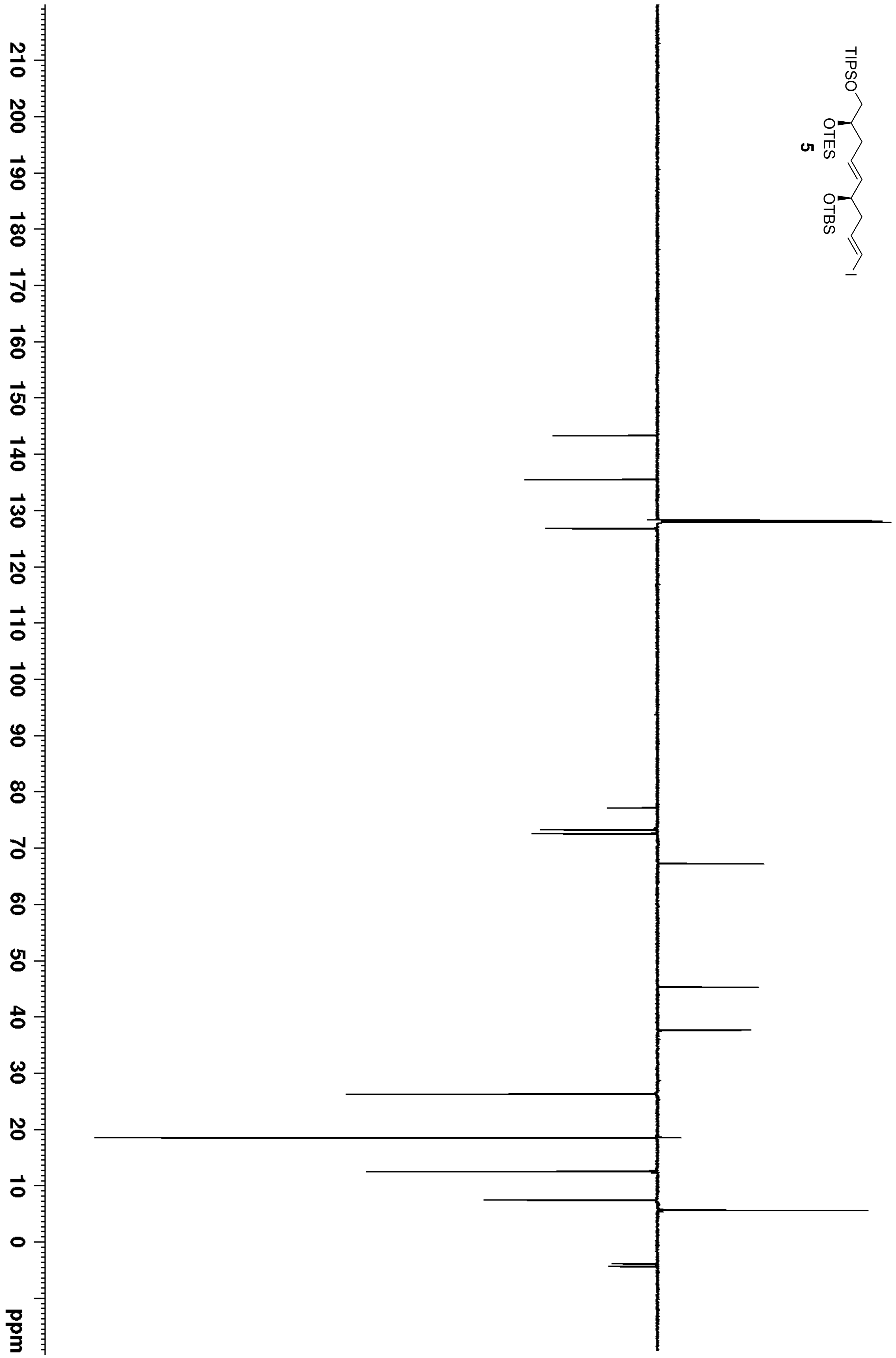

TIPSO
OTES
OTBS
I
5
210 200 190 180 170 160 150 140 130 120 110 100 90 80 70 60 50 40 30 20 10 0
ppm

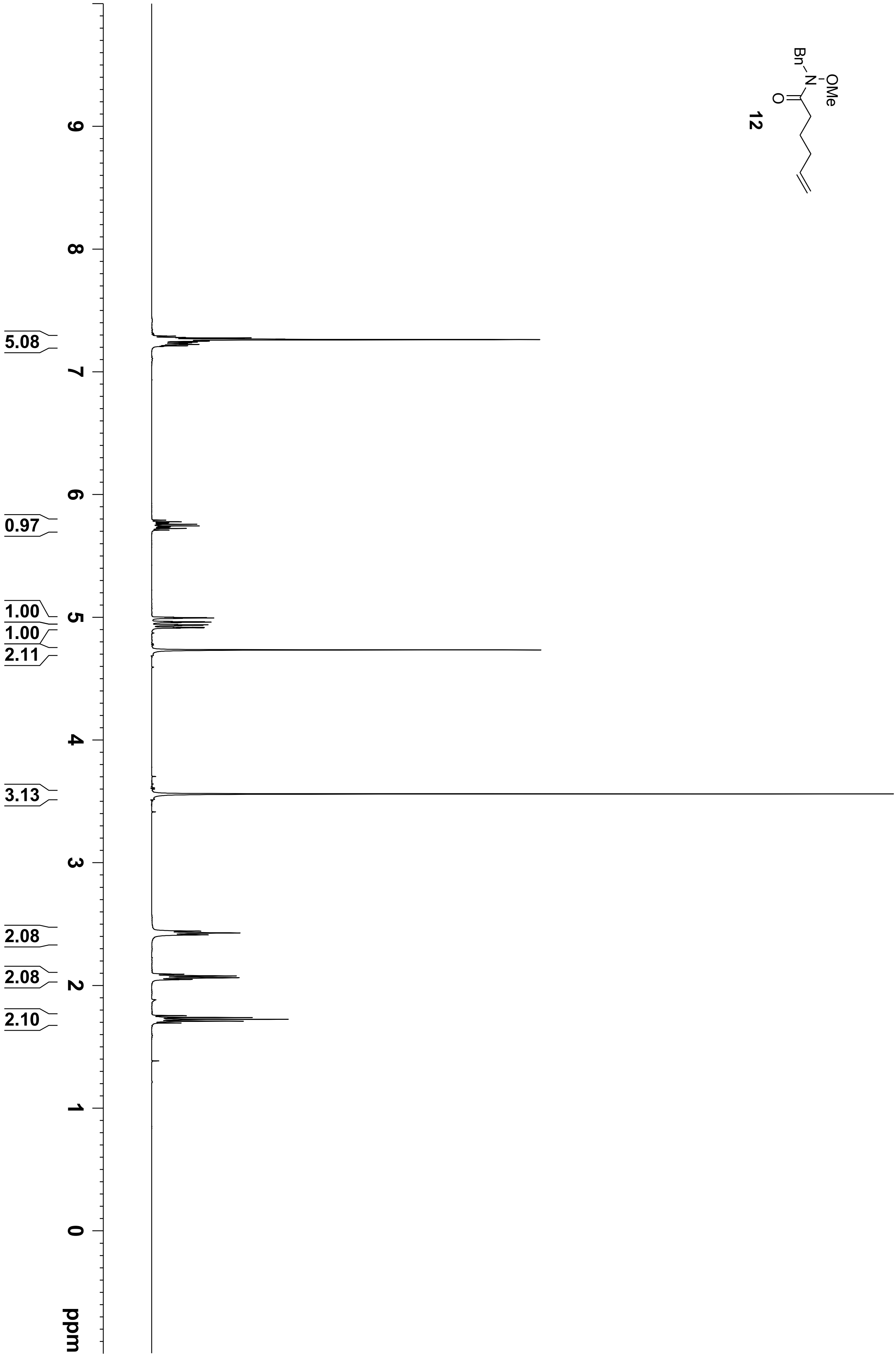

Bn
N
OMe
O
12
5.08
0.97
1.00
1.00
2.11
3.13
2.08
2.08
2.10
9
8
7
6
5
4
3
2
1
0
ppm

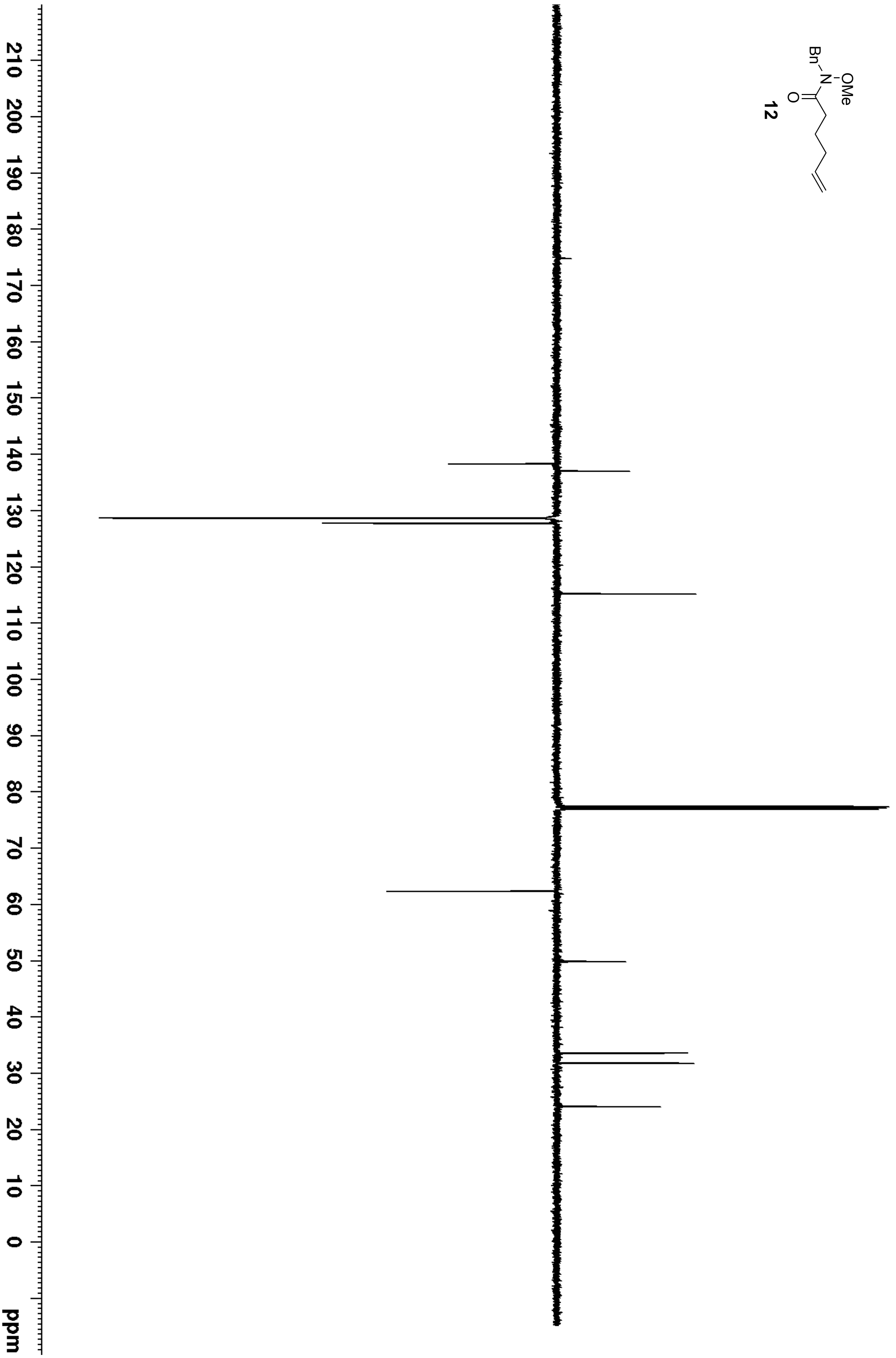
OMe
Bn
N
O
12
210
200
190
180
170
160
150
140
130
120
110
100
90
80
70
60
50
40
30
20
10
0
ppm

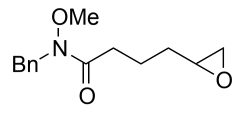
OMe
N
Bn
O
O

rac-6

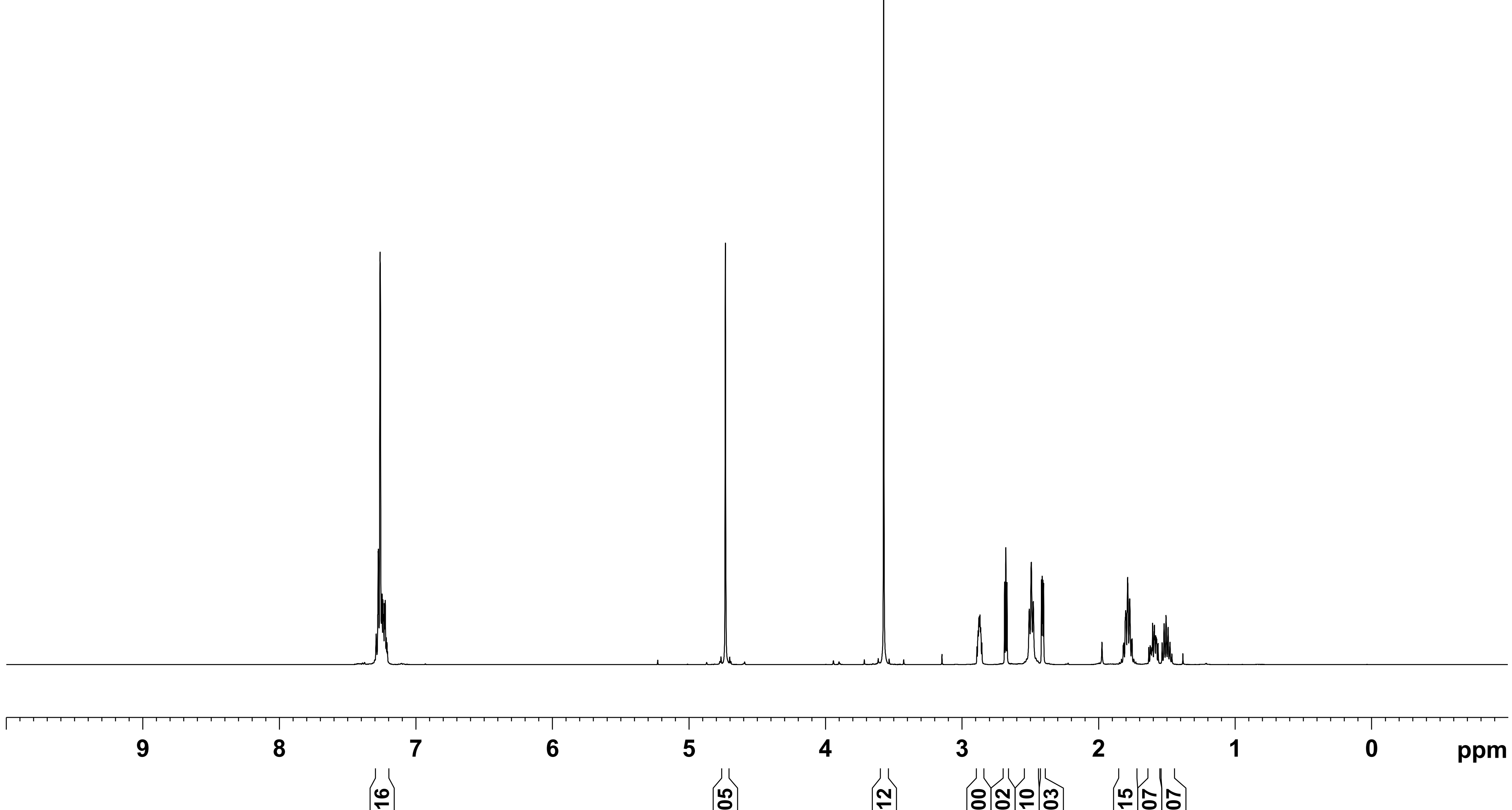
9
8
7
6
5
4
3
2
1
0
ppm
5.16
2.05
3.12
1.00
1.02
2.10
1.03
2.15
1.07
1.07

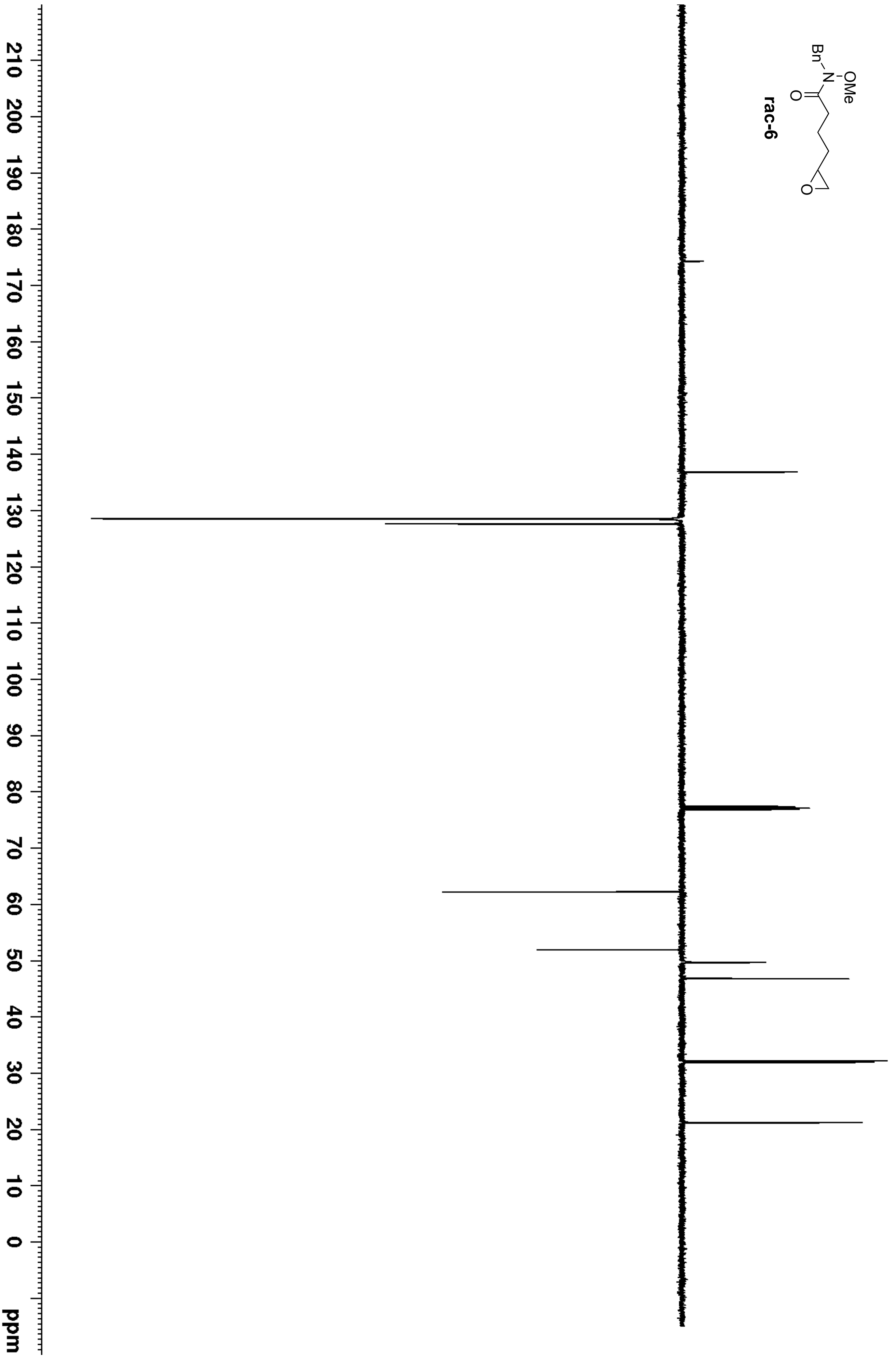

OMe
Bn
N
O
rac-6
210
200
190
180
170
160
150
140
130
120
110
100
90
80
70
60
50
40
30
20
10
0
ppm

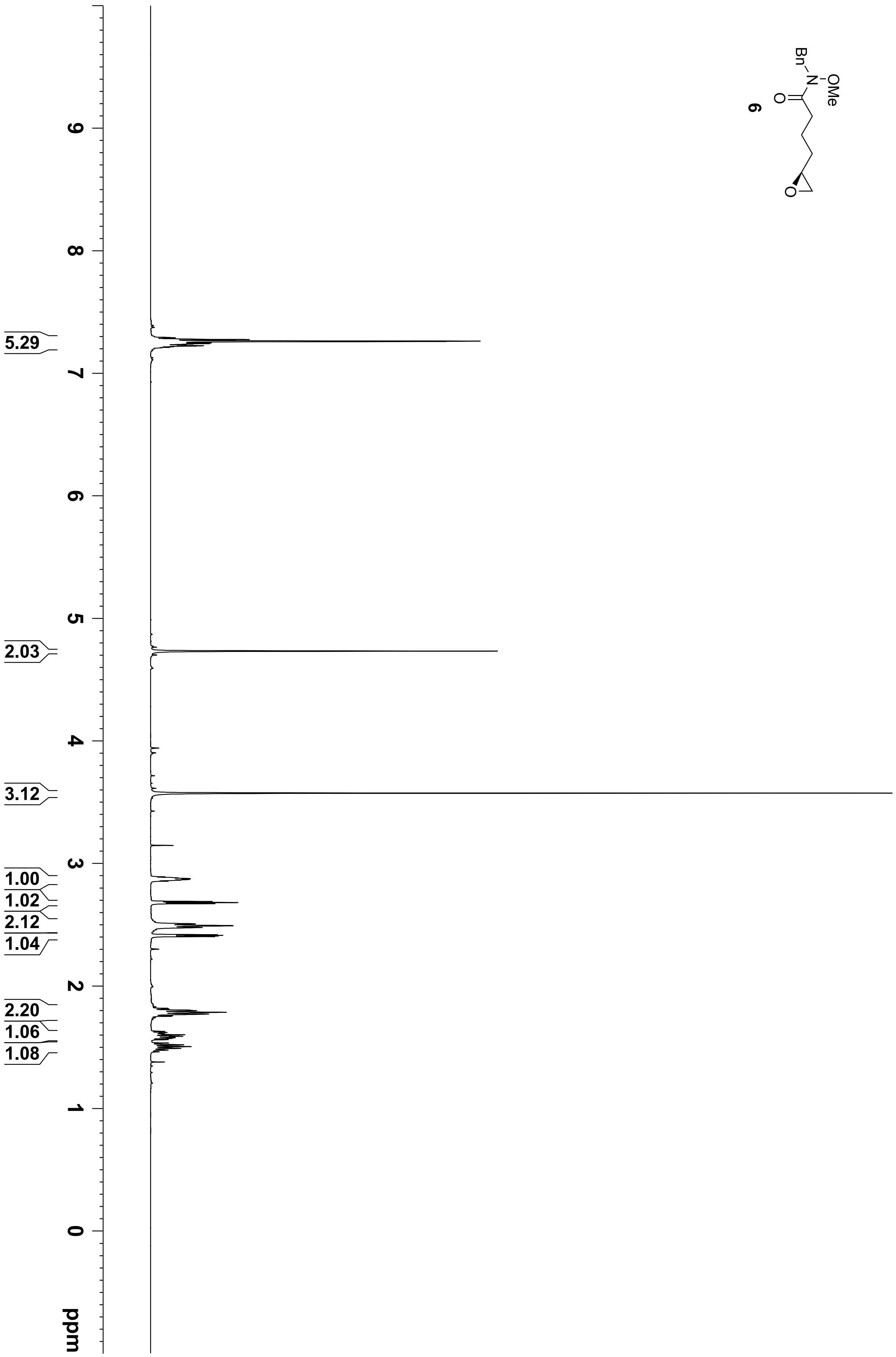

OMe
Bn
N
O
6
5.29
2.03
3.12
1.00
1.02
2.12
1.04
2.20
1.06
1.08
ppm

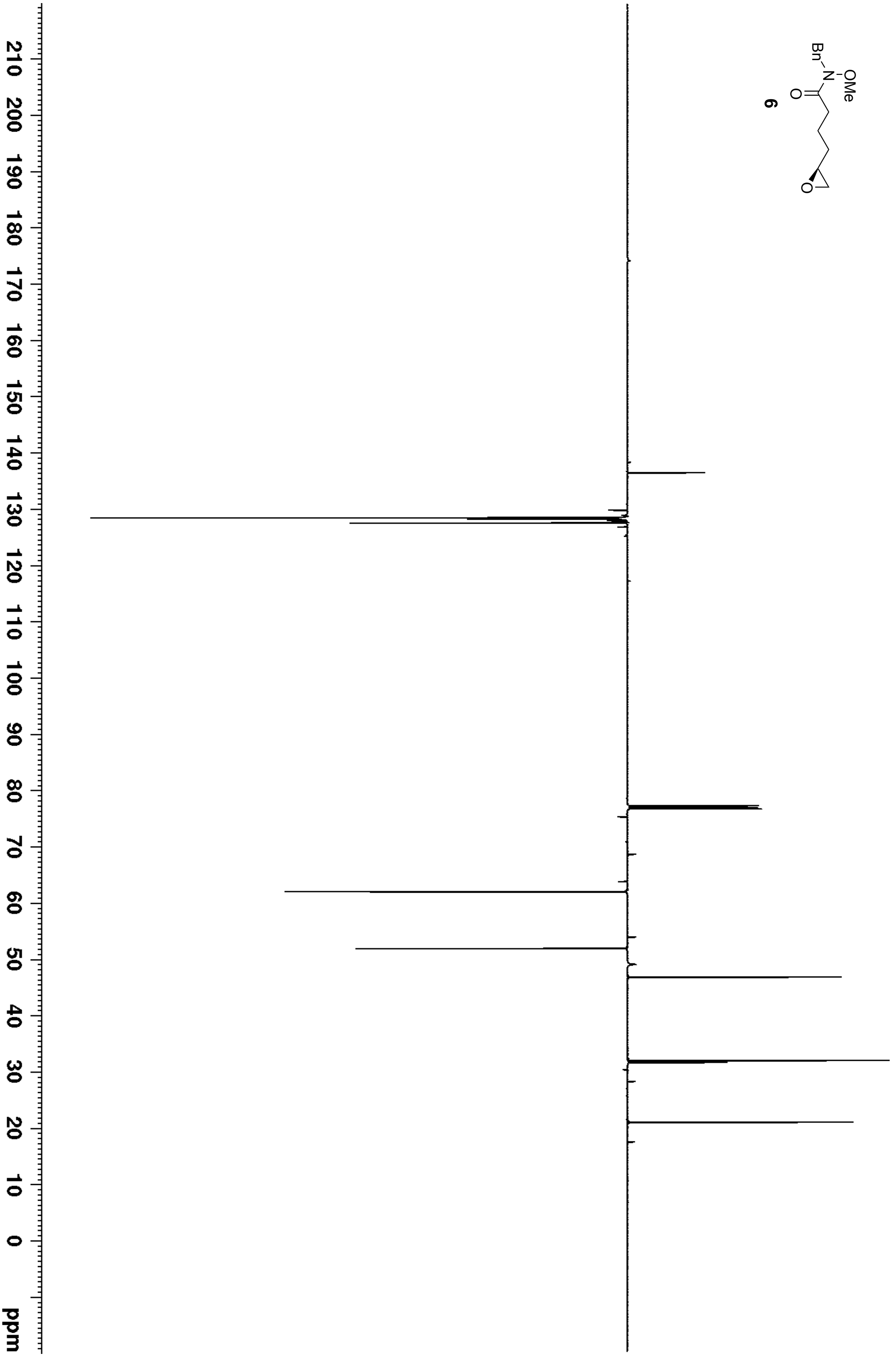
OMe
Bn
N
O
O
6
210 200 190 180 170 160 150 140 130 120 110 100 90 80 70 60 50 40 30 20 10 0 ppm

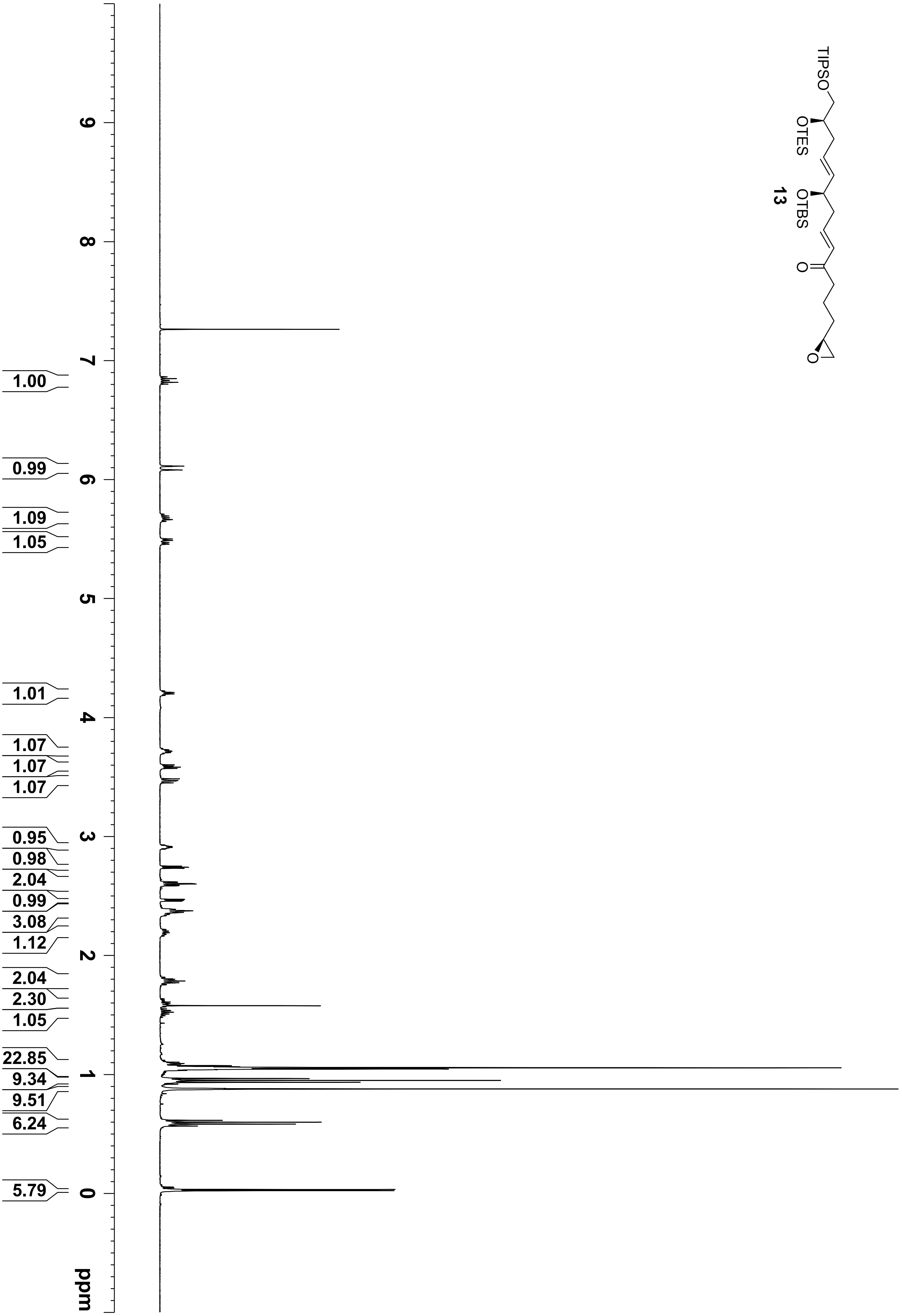
TIPSO
OTES
OTBS
O
13
1.00
0.99
1.09
1.05
1.01
1.07
1.07
1.07
0.95
0.98
2.04
0.99
3.08
1.12
2.04
2.30
1.05
22.85
9.34
9.51
6.24
5.79
9
8
7
6
5
4
3
2
1
0
ppm

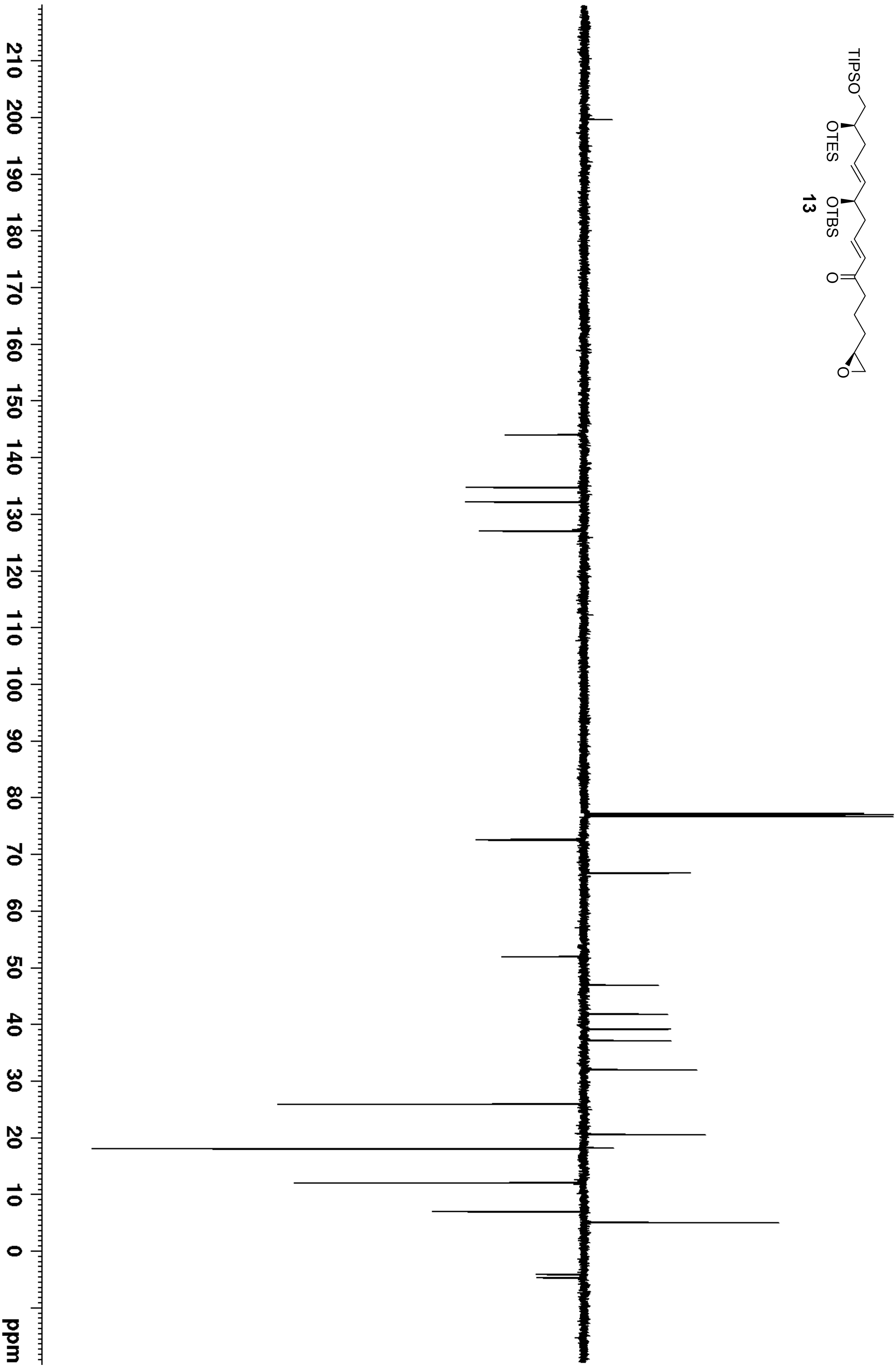
TIPSO
OTES
OTBS
O
O
13
210 200 190 180 170 160 150 140 130 120 110 100 90 80 70 60 50 40 30 20 10 0 ppm

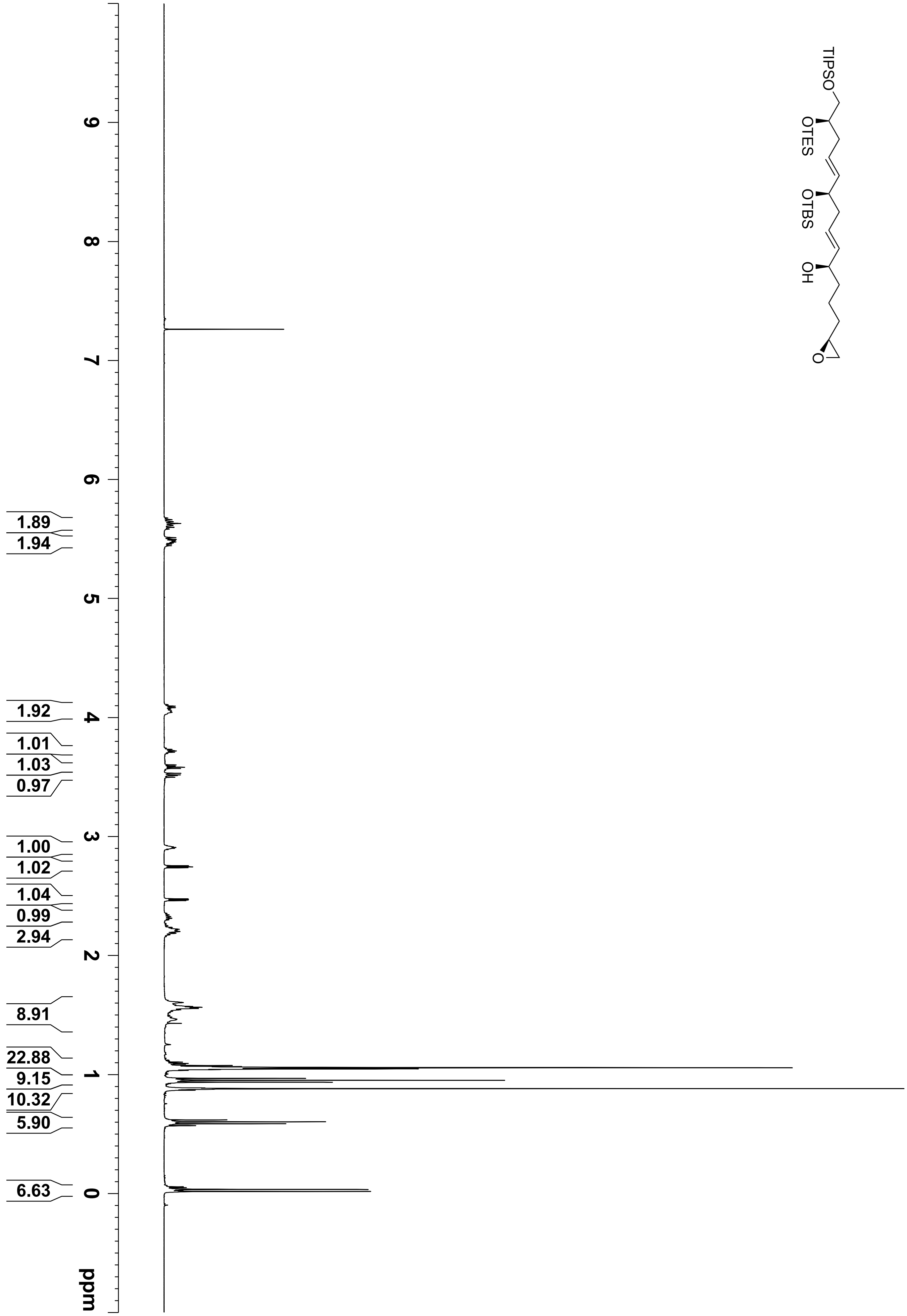
TIPSO
OTES
OTBS
OH
O
9
8
7
6
5
4
3
2
1
0
ppm
1.89
1.94
1.92
1.01
1.03
0.97
1.00
1.02
1.04
0.99
2.94
8.91
22.88
9.15
10.32
5.90
6.63

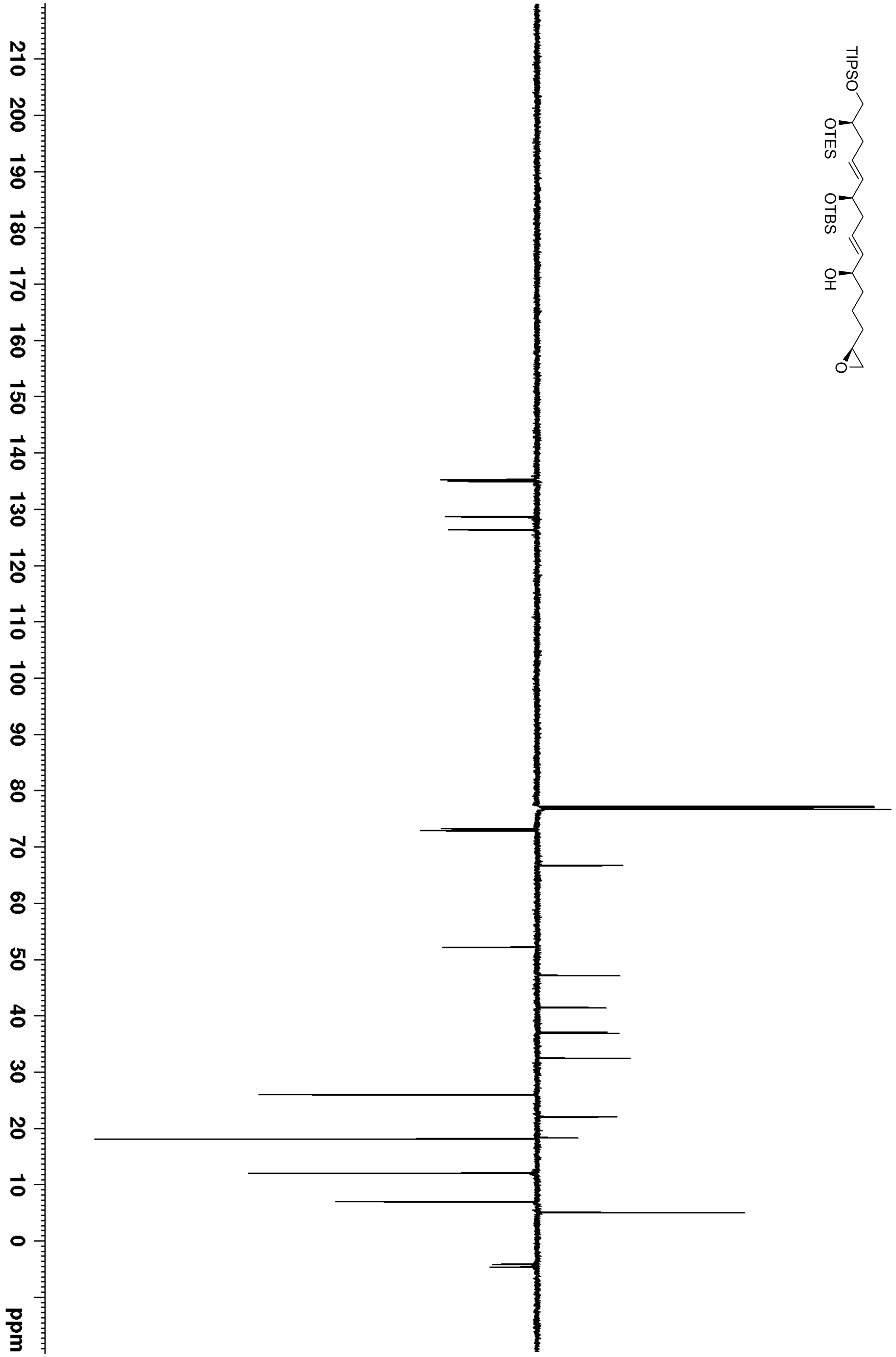

TIPSO
OTES
OTBS
OH
O
ppm

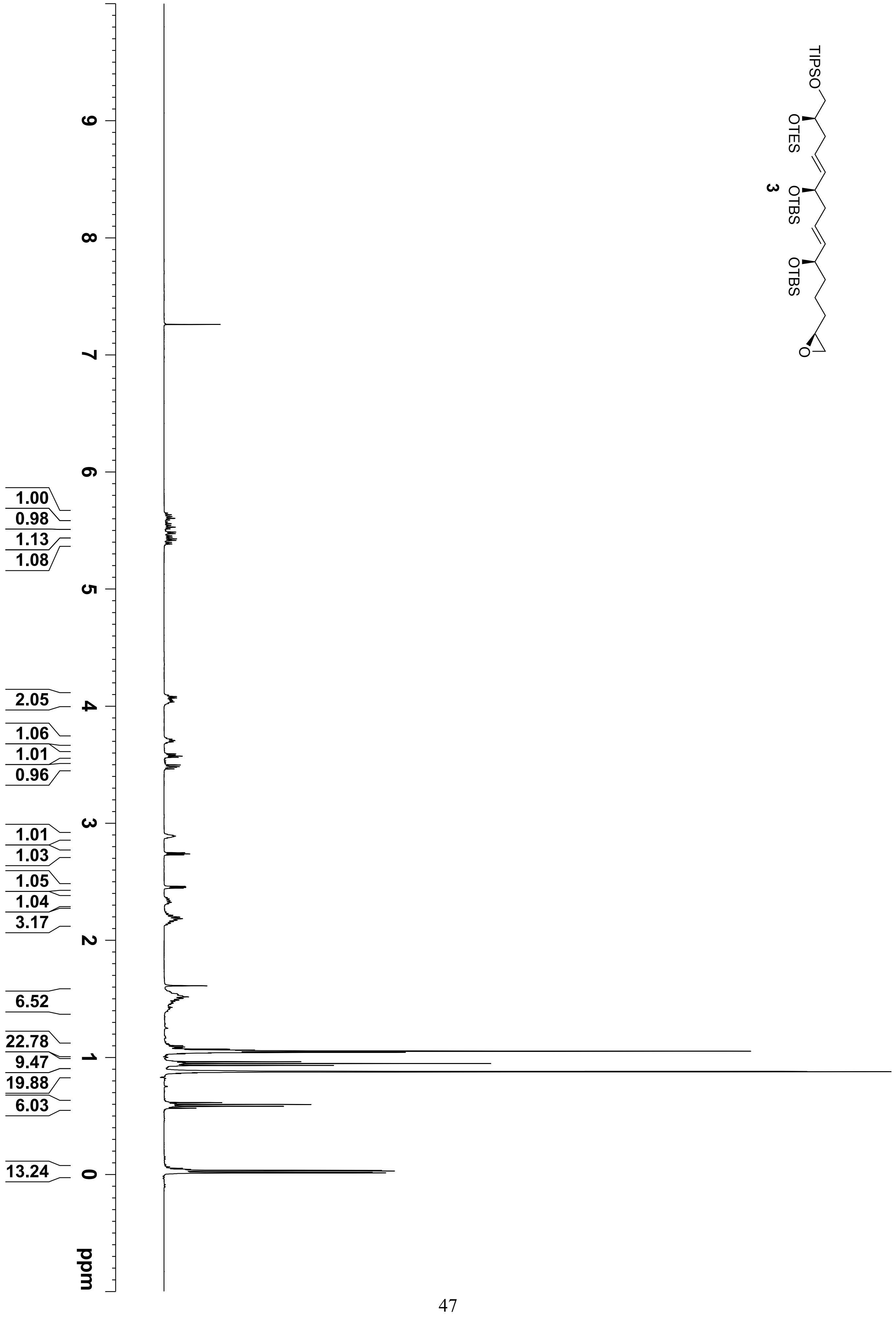

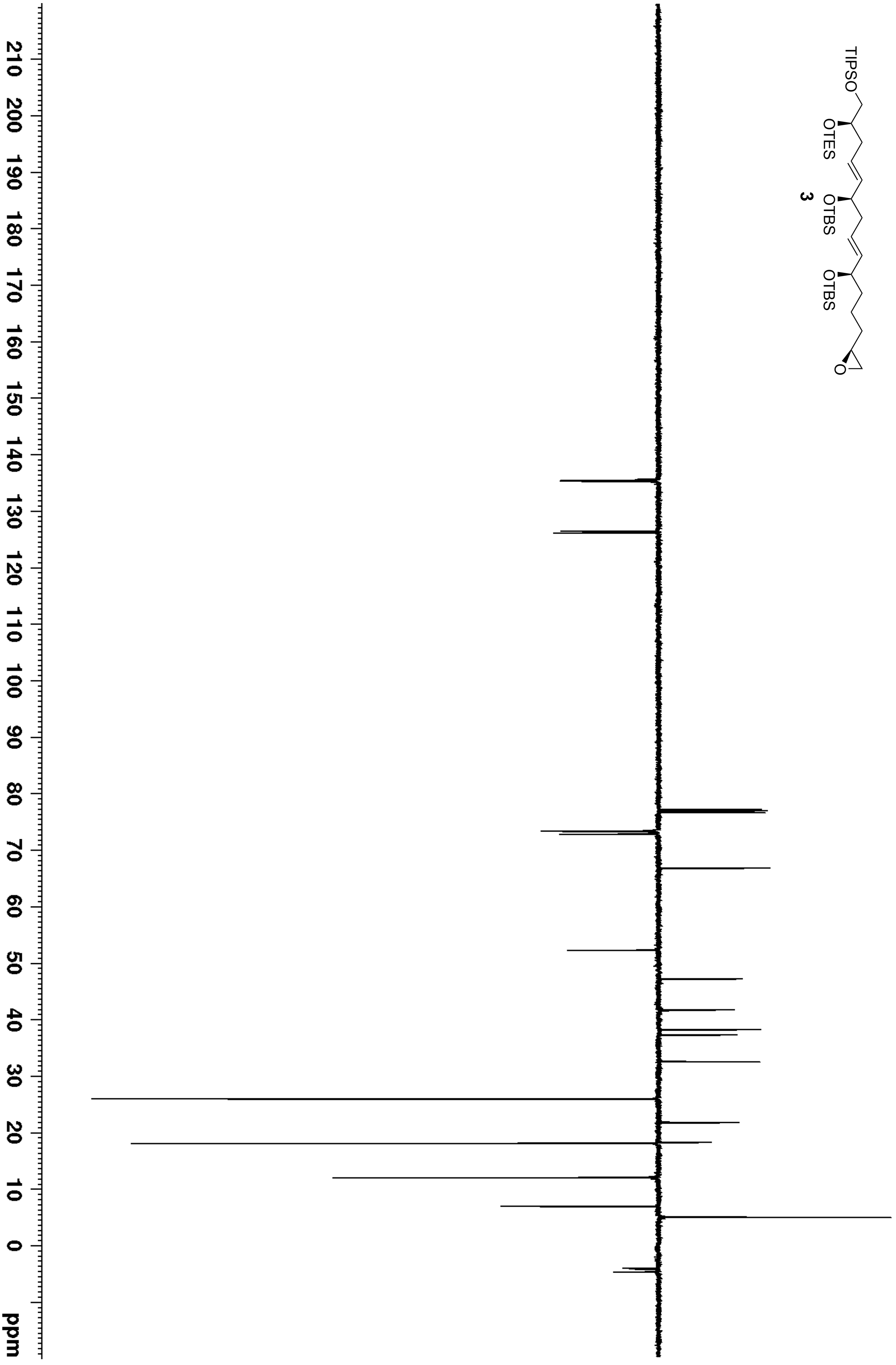
TIPSO
OTES
OTBS
OTBS
O
3
210 200 190 180 170 160 150 140 130 120 110 100 90 80 70 60 50 40 30 20 10 0
ppm

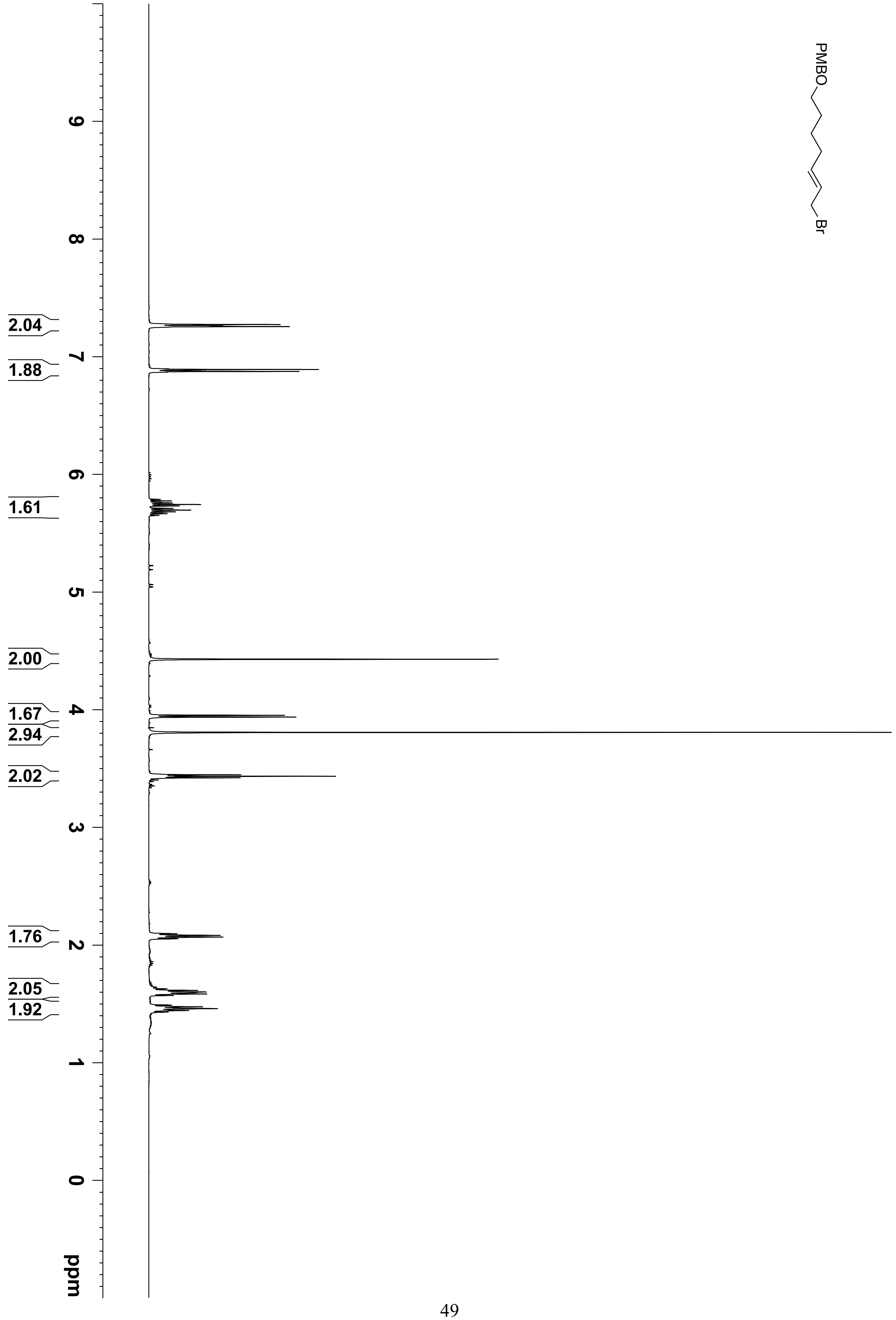
PMBO
Br
2.04
1.88
1.61
2.00
1.67
2.94
2.02
1.76
2.05
1.92
9
8
7
6
5
4
3
2
1
0
ppm

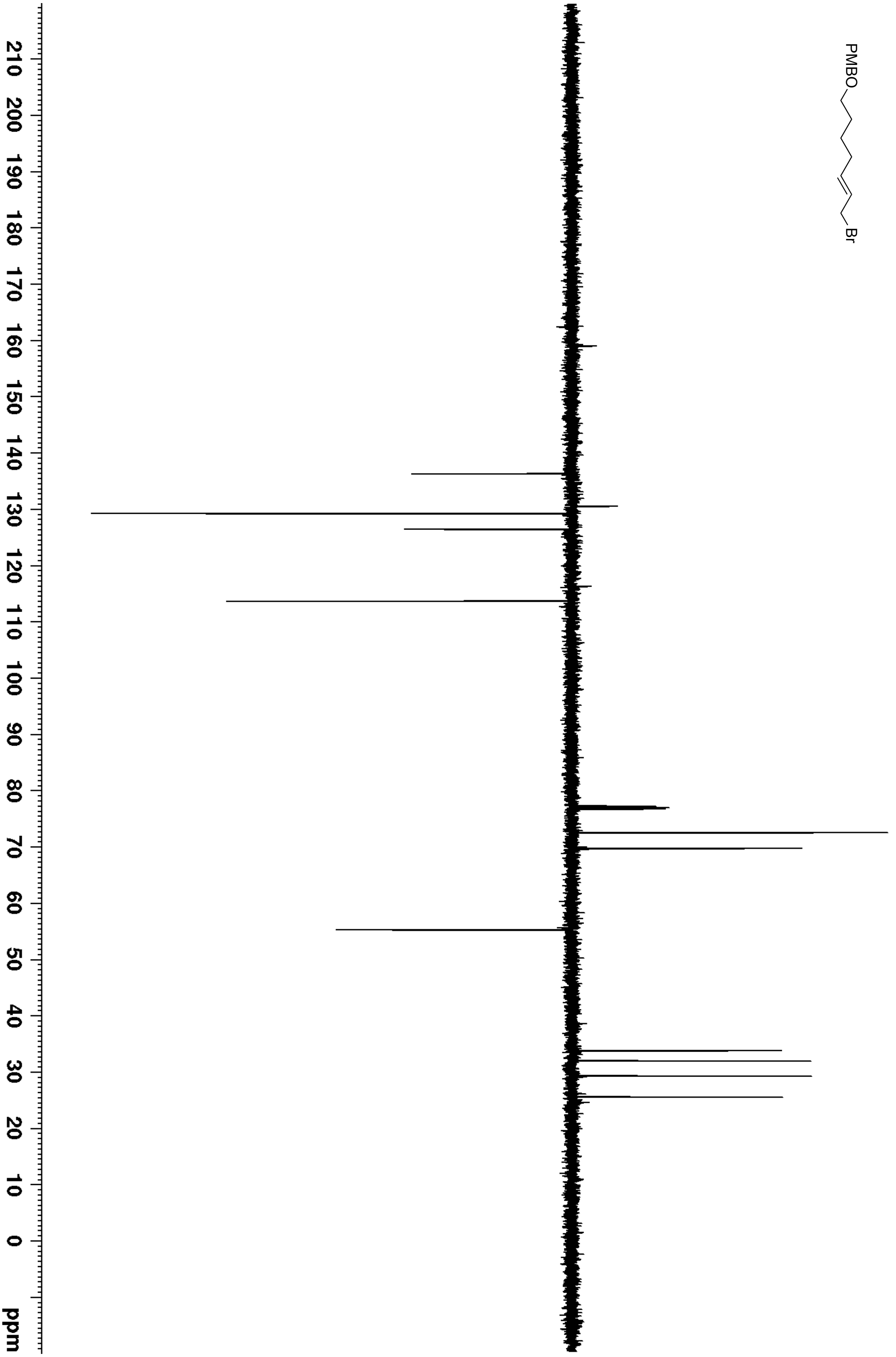
PMBO
Br
210
200
190
180
170
160
150
140
130
120
110
100
90
80
70
60
50
40
30
20
10
0
ppm

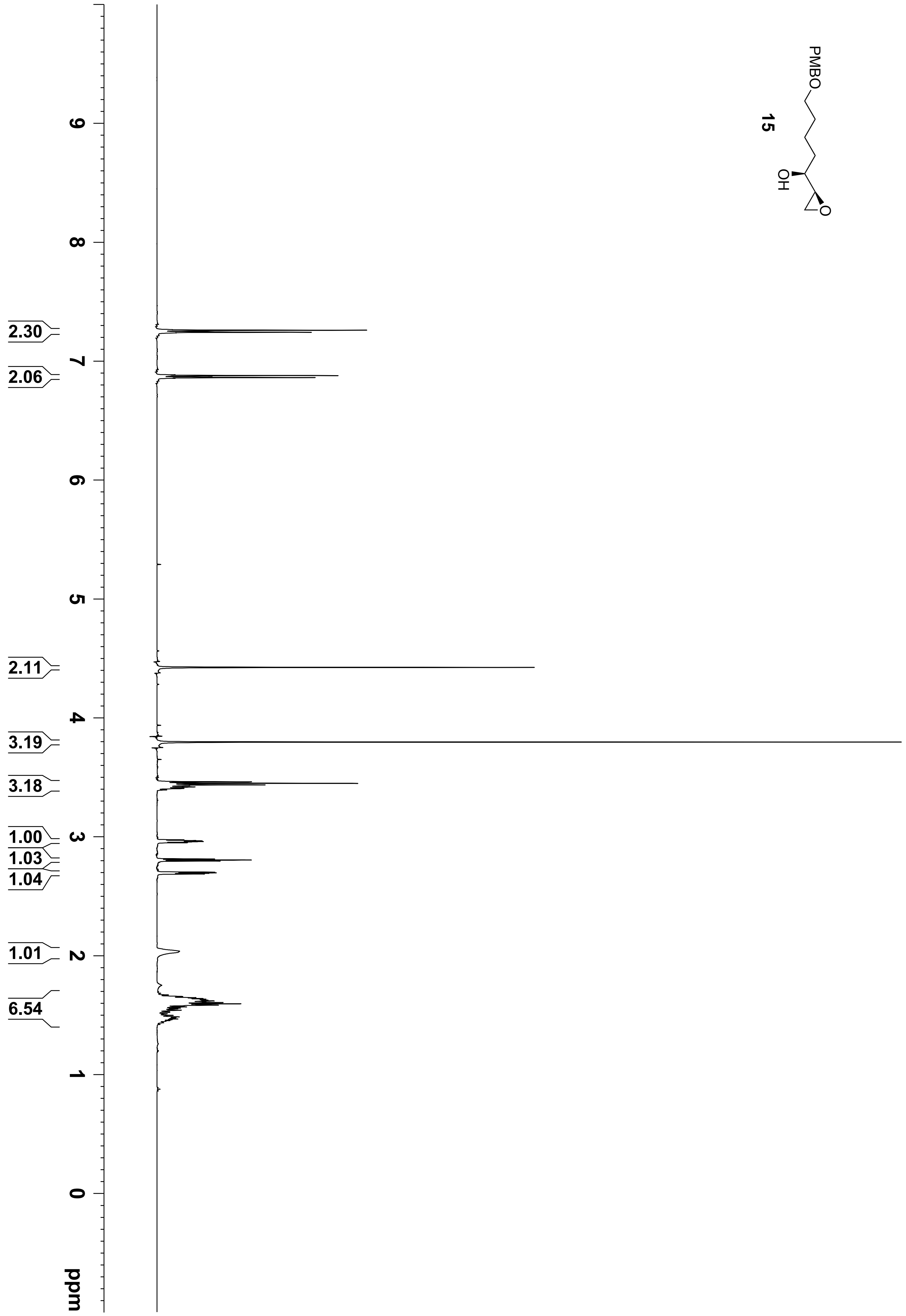
PMBO
OH
O
15
2.30
2.06
2.11
3.19
3.18
1.00
1.03
1.04
1.01
6.54
9
8
7
6
5
4
3
2
1
0
ppm

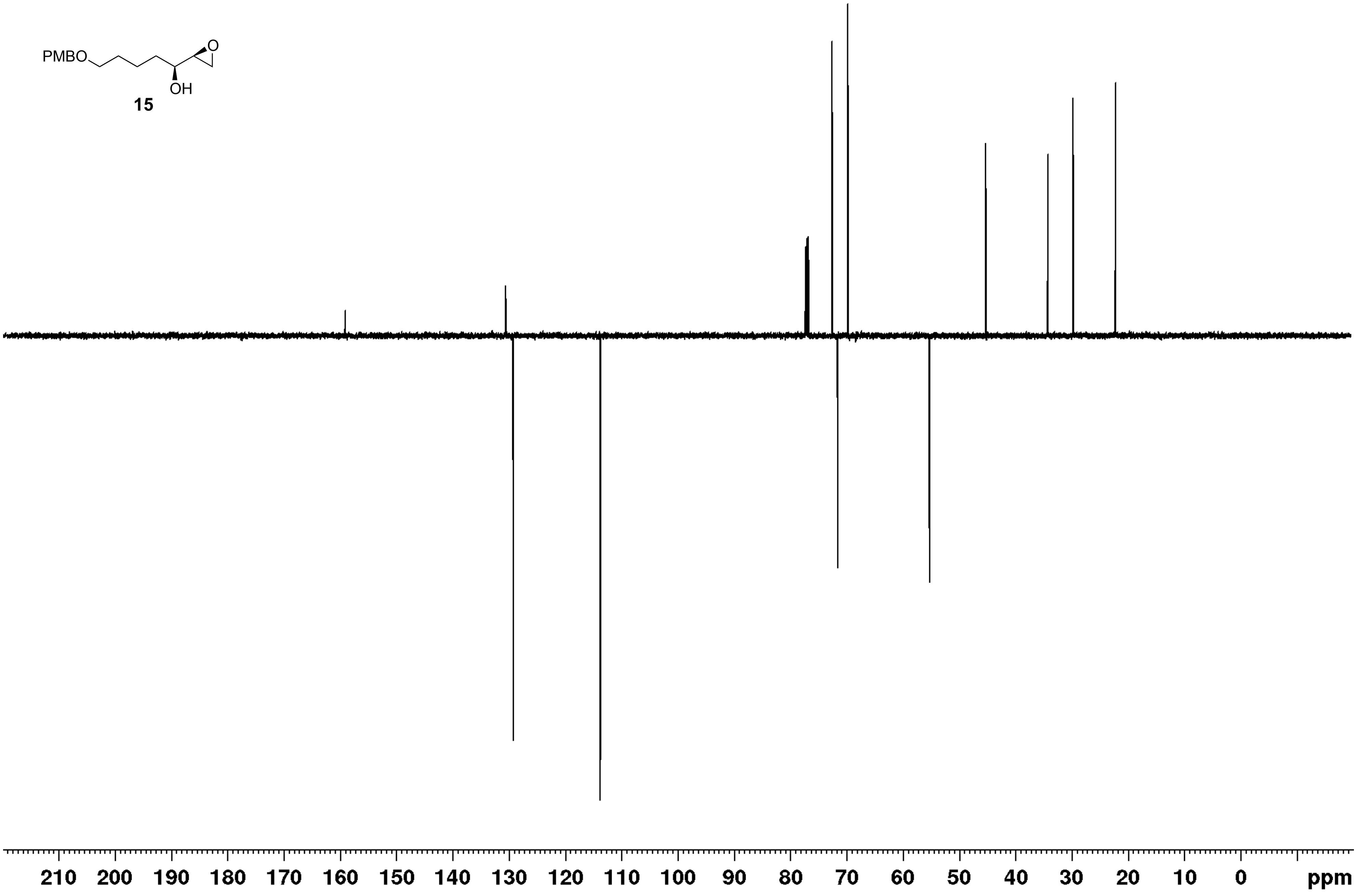
PMBO
OH
O
15
210 200 190 180 170 160 150 140 130 120 110 100 90 80 70 60 50 40 30 20 10 0 ppm

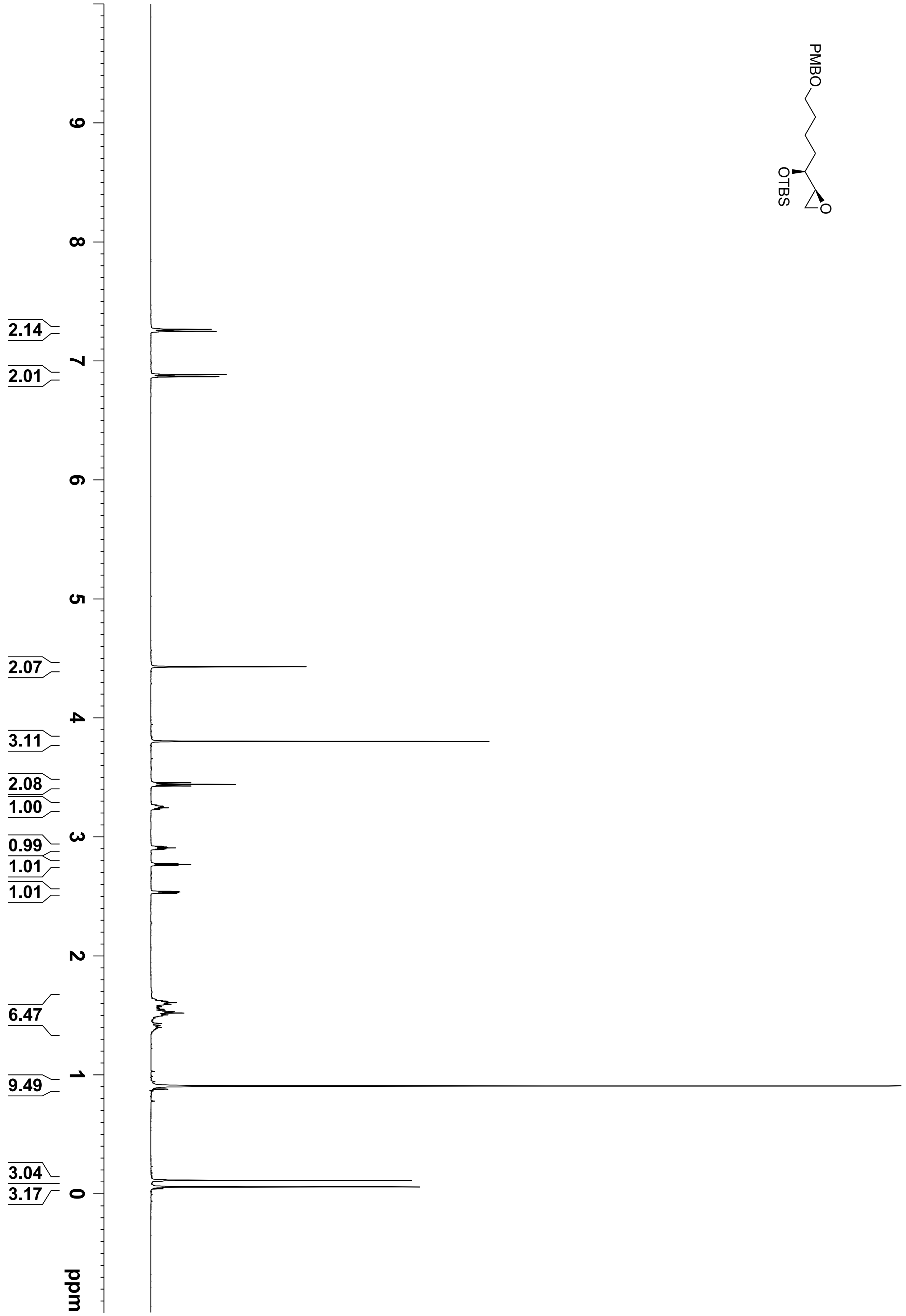
PMBO
OTBS
O
2.14
2.01
2.07
3.11
2.08
1.00
0.99
1.01
1.01
6.47
9.49
3.04
3.17
9
8
7
6
5
4
3
2
1
0
ppm

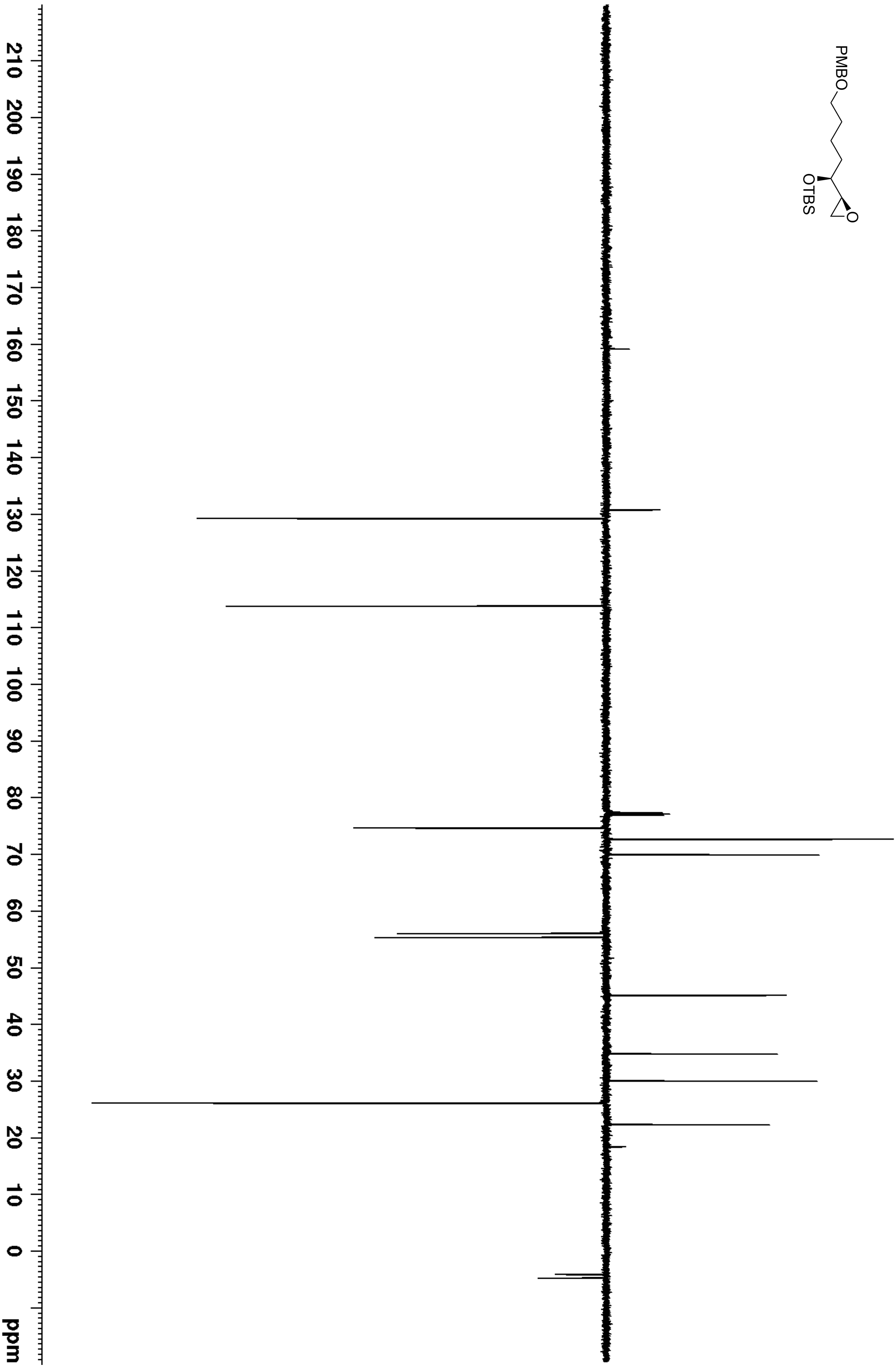
PMBO
OTBS
O
210
200
190
180
170
160
150
140
130
120
110
100
90
80
70
60
50
40
30
20
10
0
ppm

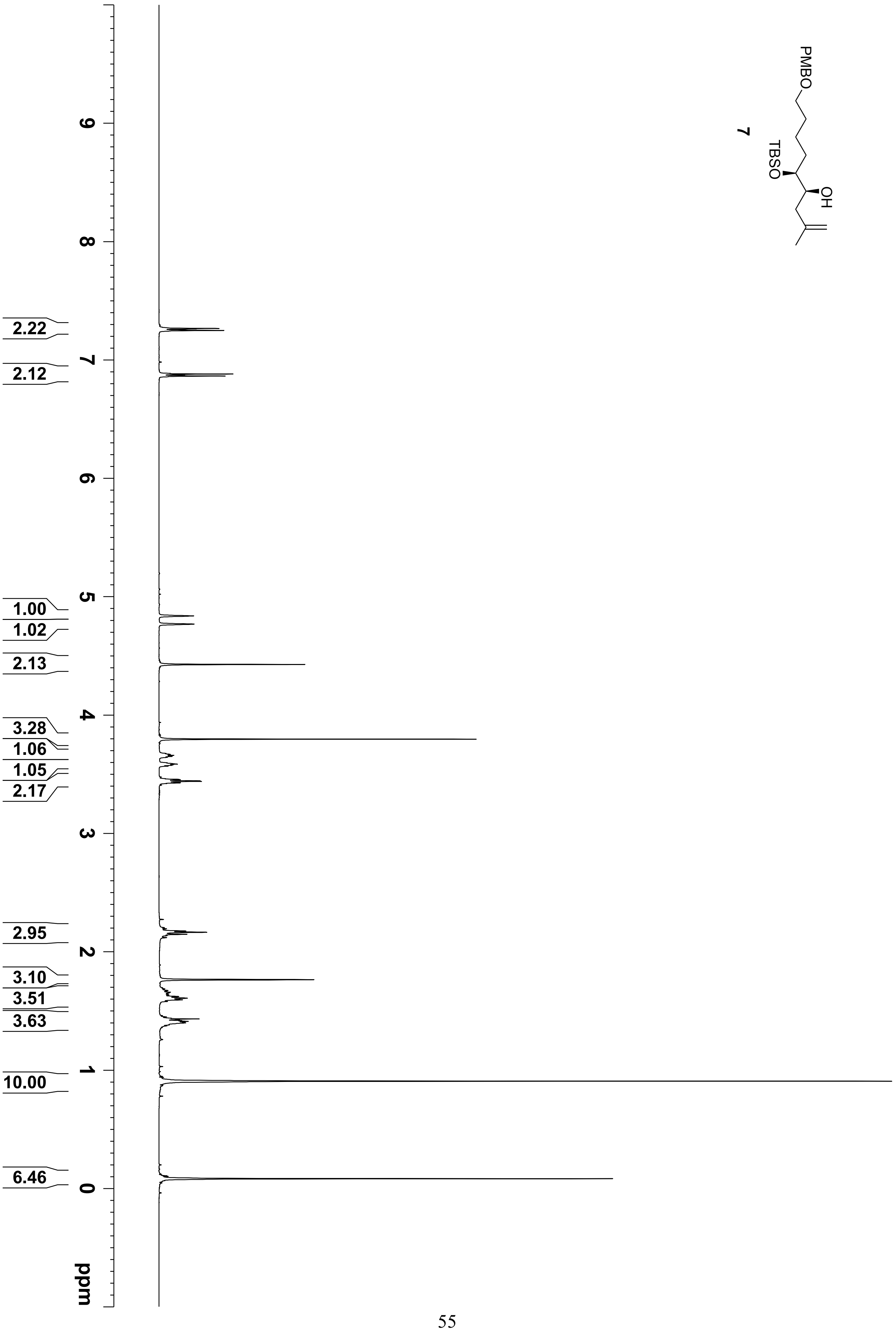
PMBO
TBSO
OH
7
2.22
2.12
1.00
1.02
2.13
3.28
1.06
1.05
2.17
2.95
3.10
3.51
3.63
10.00
6.46
9
8
7
6
5
4
3
2
1
0
ppm

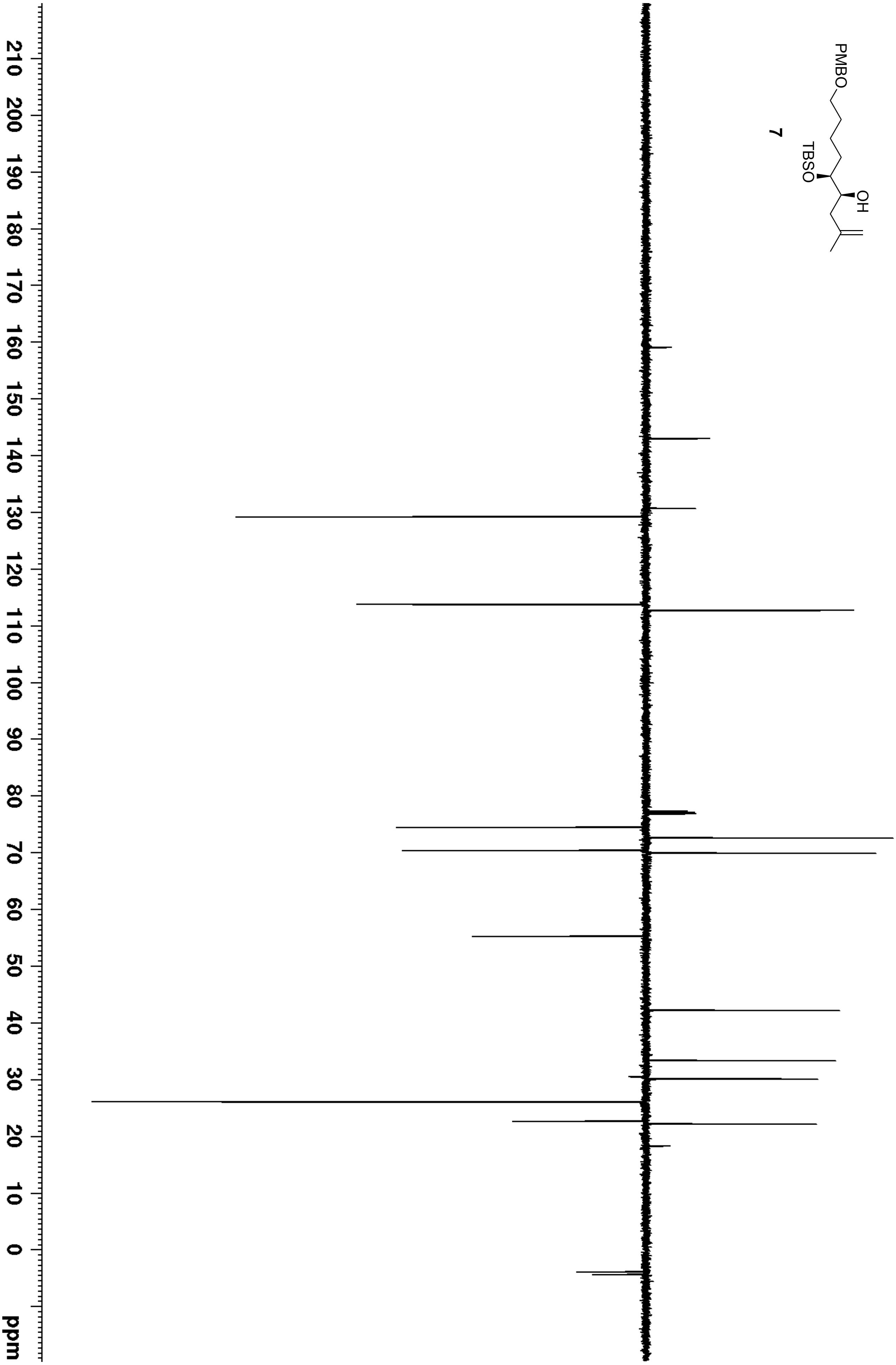
PMBO
TBSO
OH
7
210 200 190 180 170 160 150 140 130 120 110 100 90 80 70 60 50 40 30 20 10 0 ppm

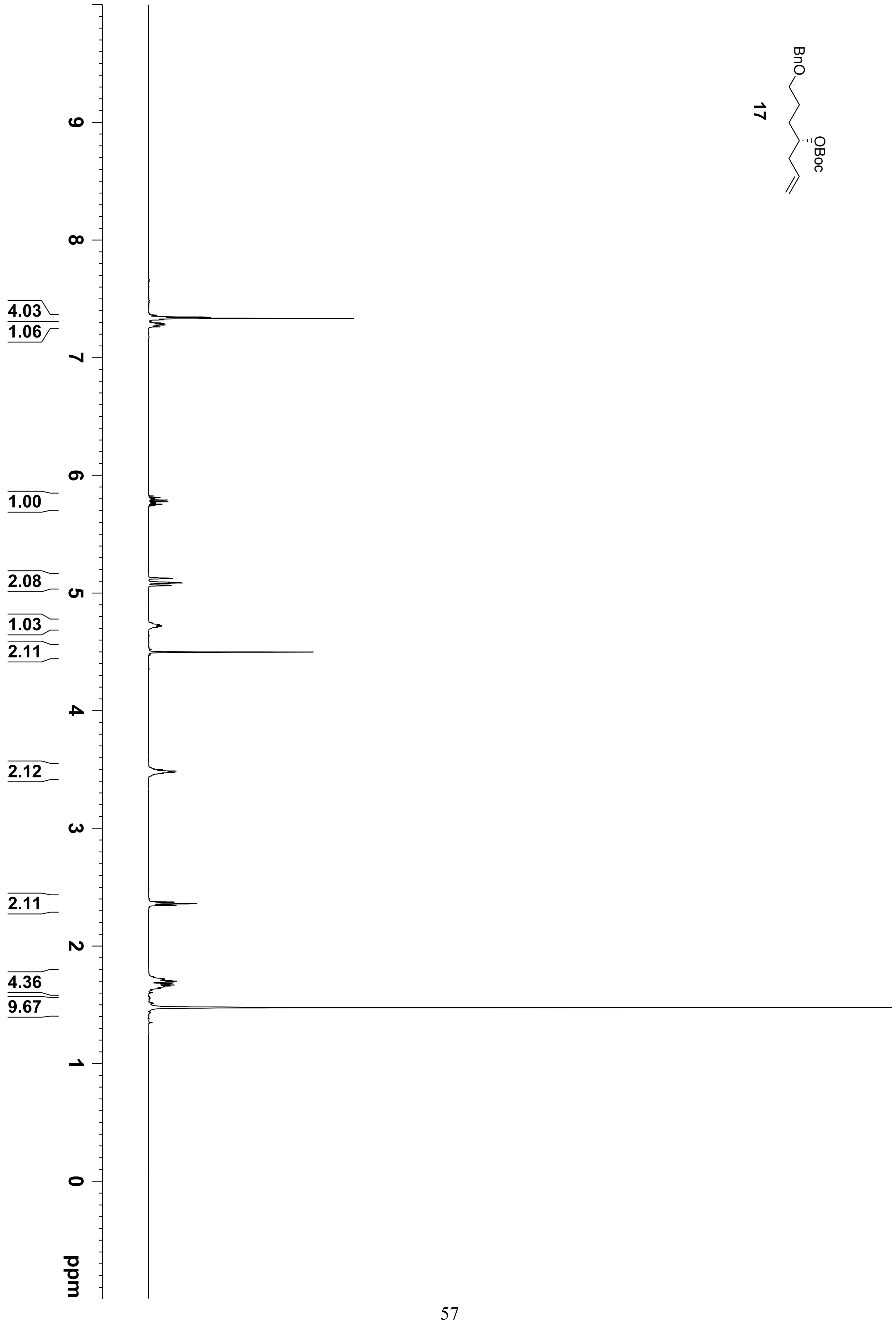
BnO
OBoc
17
4.03
1.06
1.00
2.08
1.03
2.11
2.12
2.11
4.36
9.67
9
8
7
6
5
4
3
2
1
0
ppm

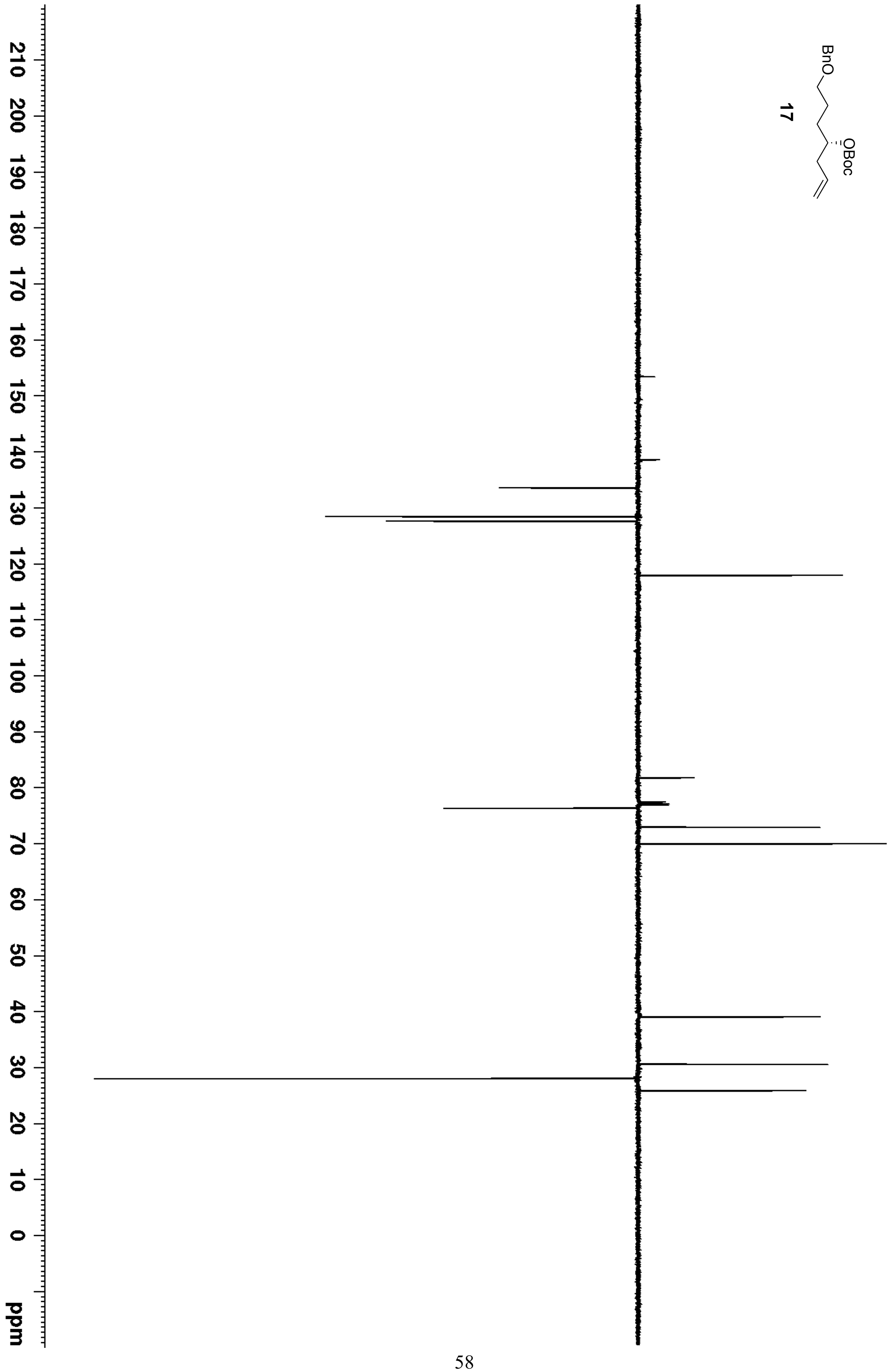
BnO
OBoc
17
210 200 190 180 170 160 150 140 130 120 110 100 90 80 70 60 50 40 30 20 10 0 ppm

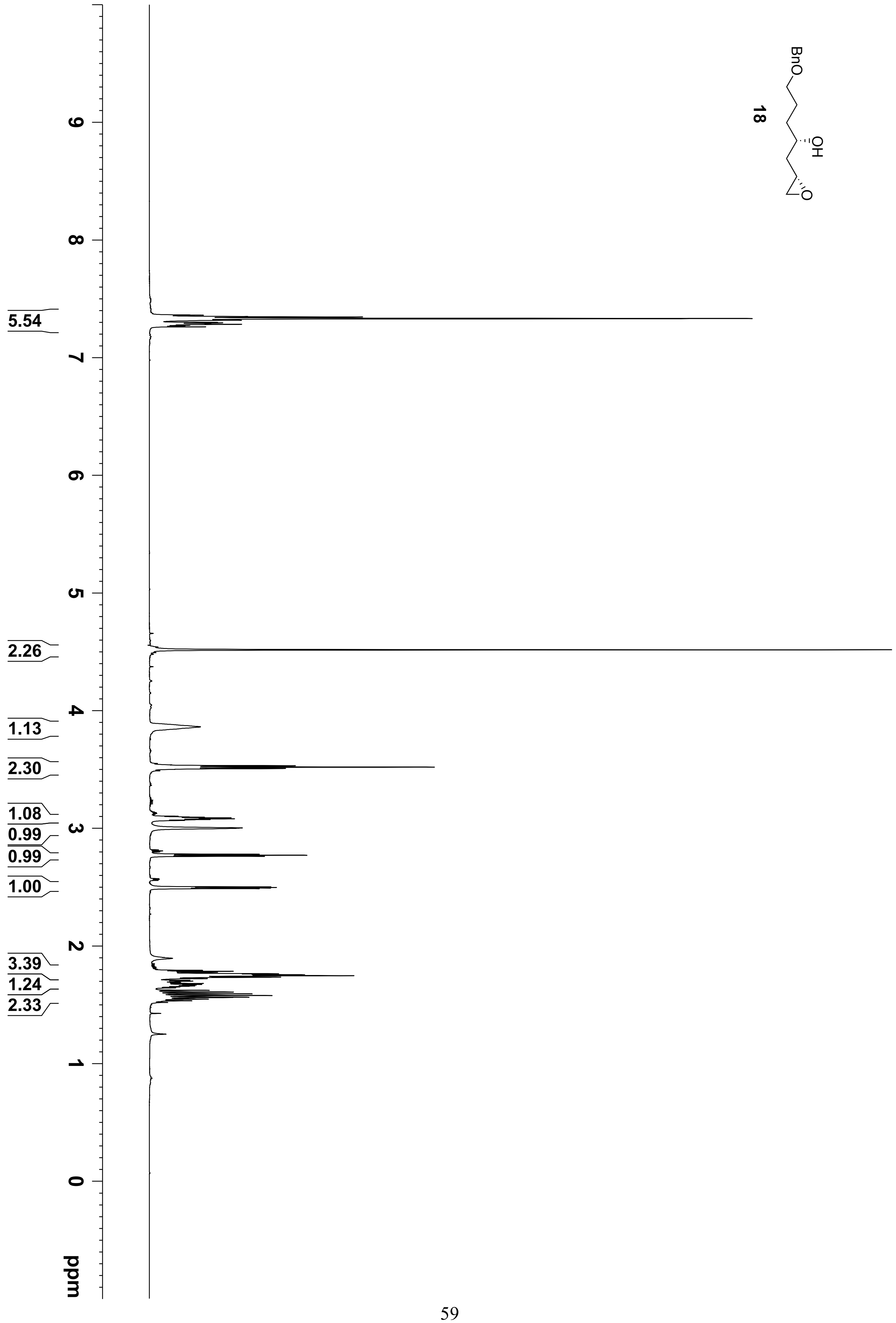
BnO
OH
O
18
5.54
2.26
1.13
2.30
1.08
0.99
0.99
1.00
3.39
1.24
2.33
9
8
7
6
5
4
3
2
1
0
ppm

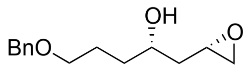
BnO
OH
O

18

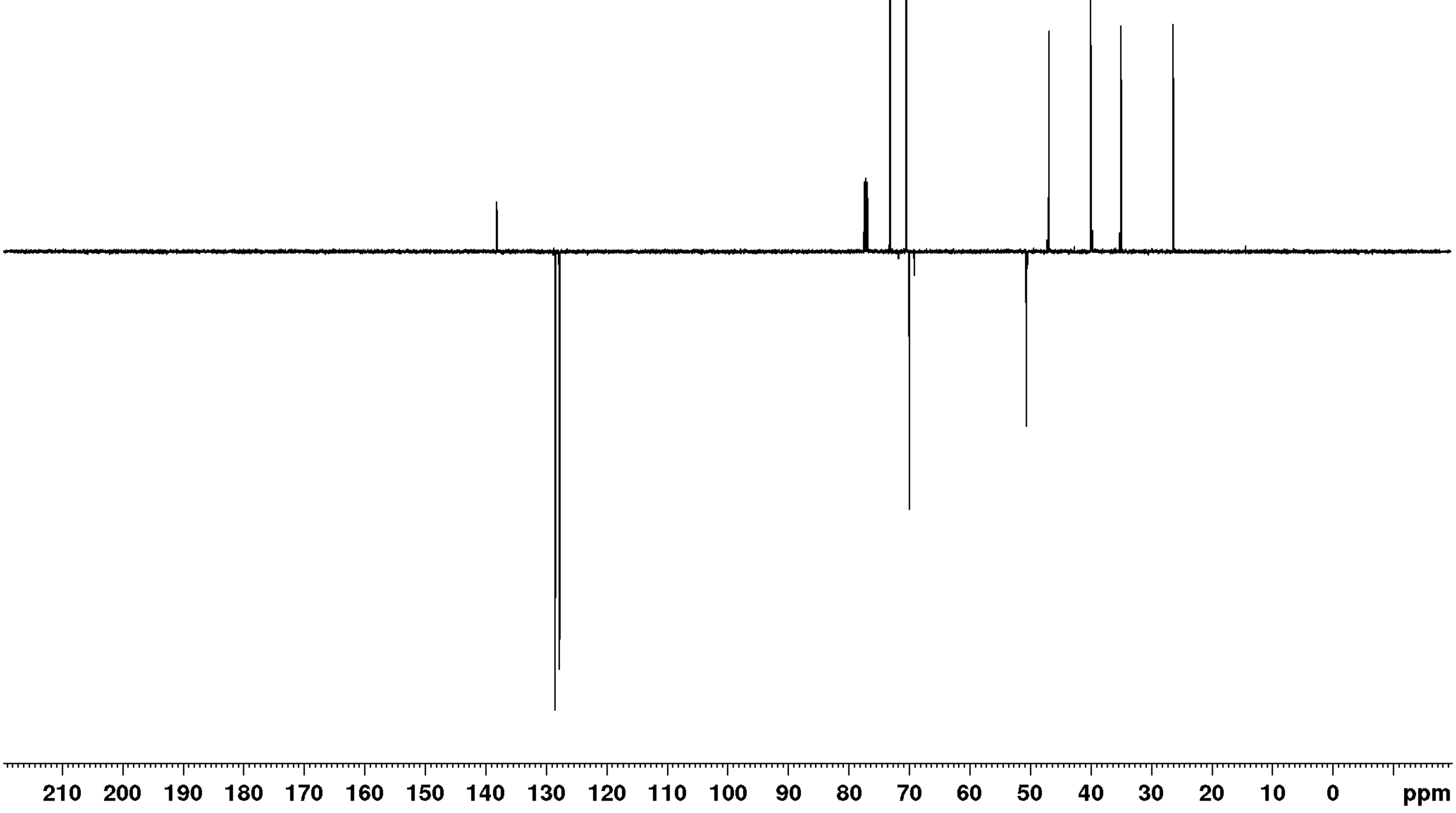
210 200 190 180 170 160 150 140 130 120 110 100 90 80 70 60 50 40 30 20 10 0 ppm

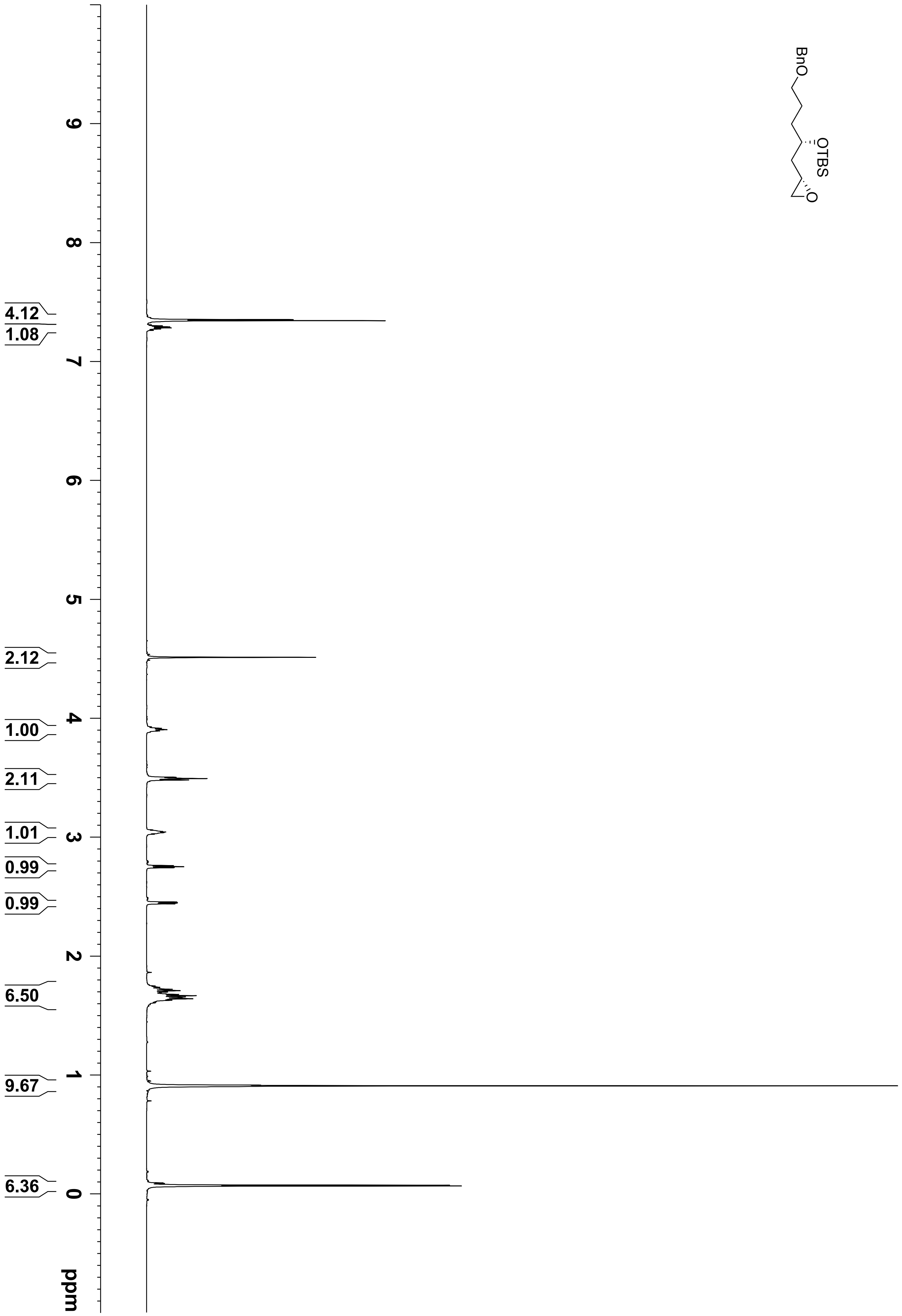
BnO
OTBS
O
4.12
1.08
2.12
1.00
2.11
1.01
0.99
0.99
6.50
9.67
6.36
ppm

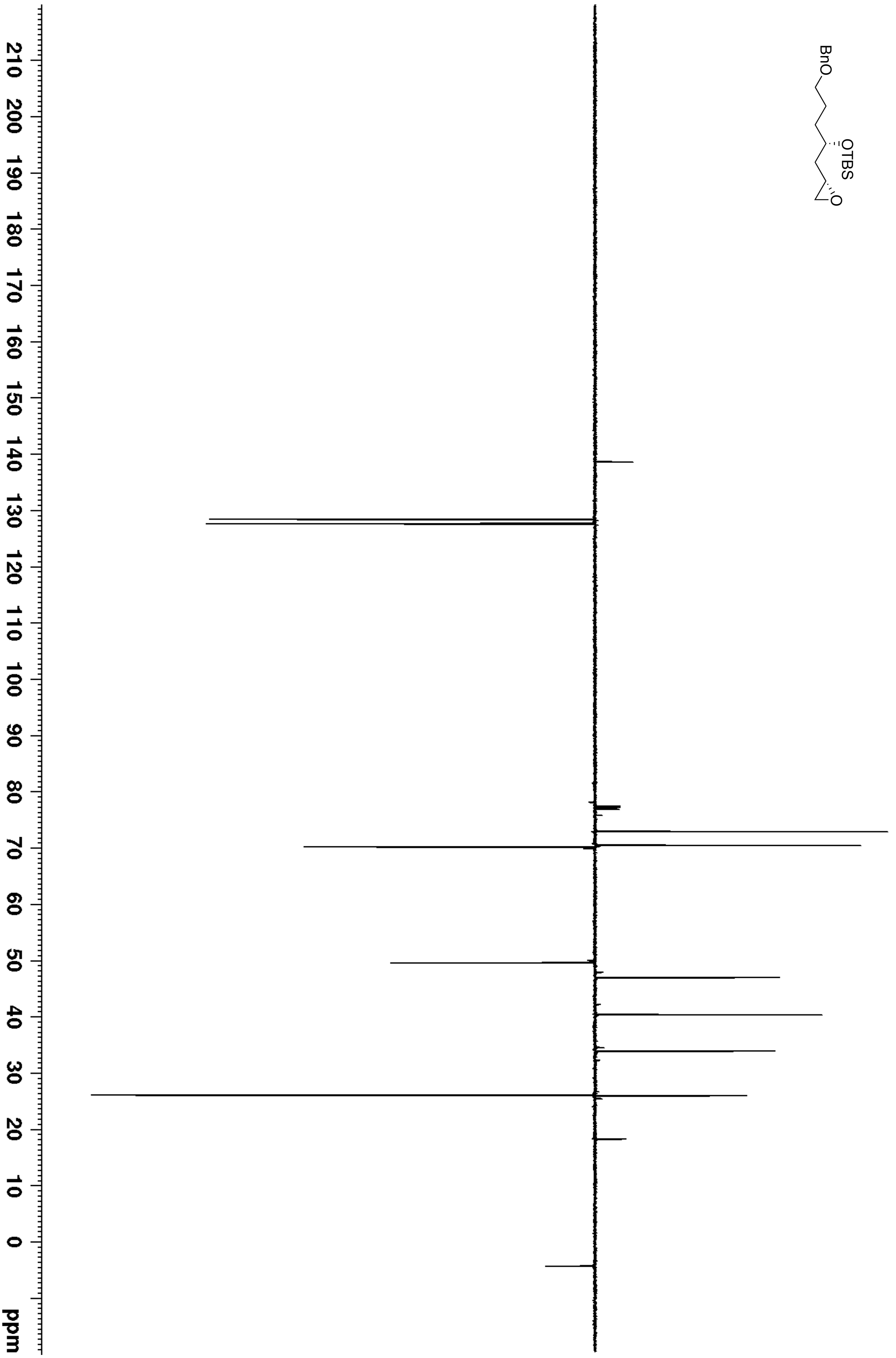
BnO
OTBS
O
210 200 190 180 170 160 150 140 130 120 110 100 90 80 70 60 50 40 30 20 10 0
ppm

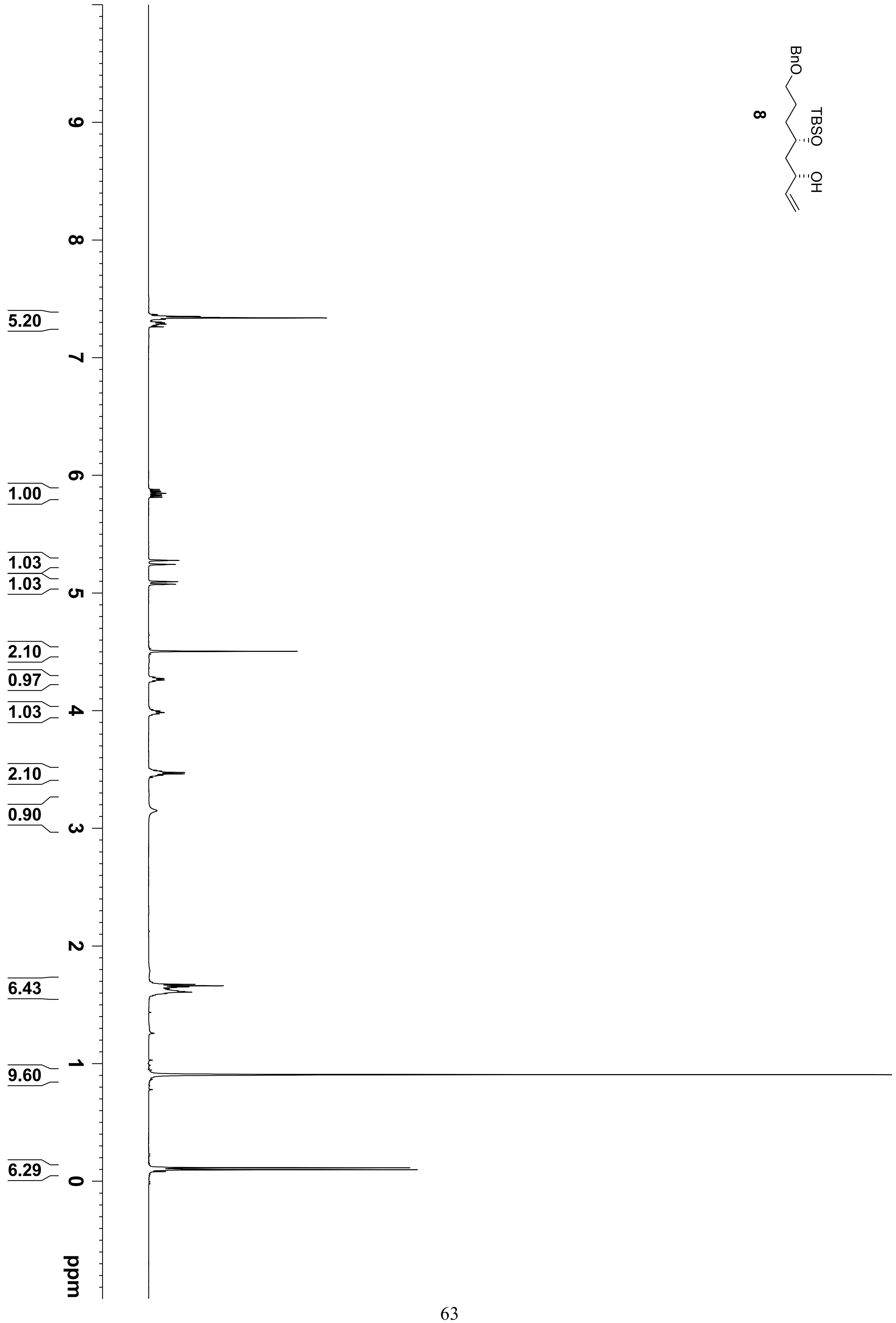
BnO
TBSO
OH
8
5.20
1.00
1.03
1.03
2.10
0.97
1.03
2.10
0.90
6.43
9.60
6.29
9
8
7
6
5
4
3
2
1
0
ppm

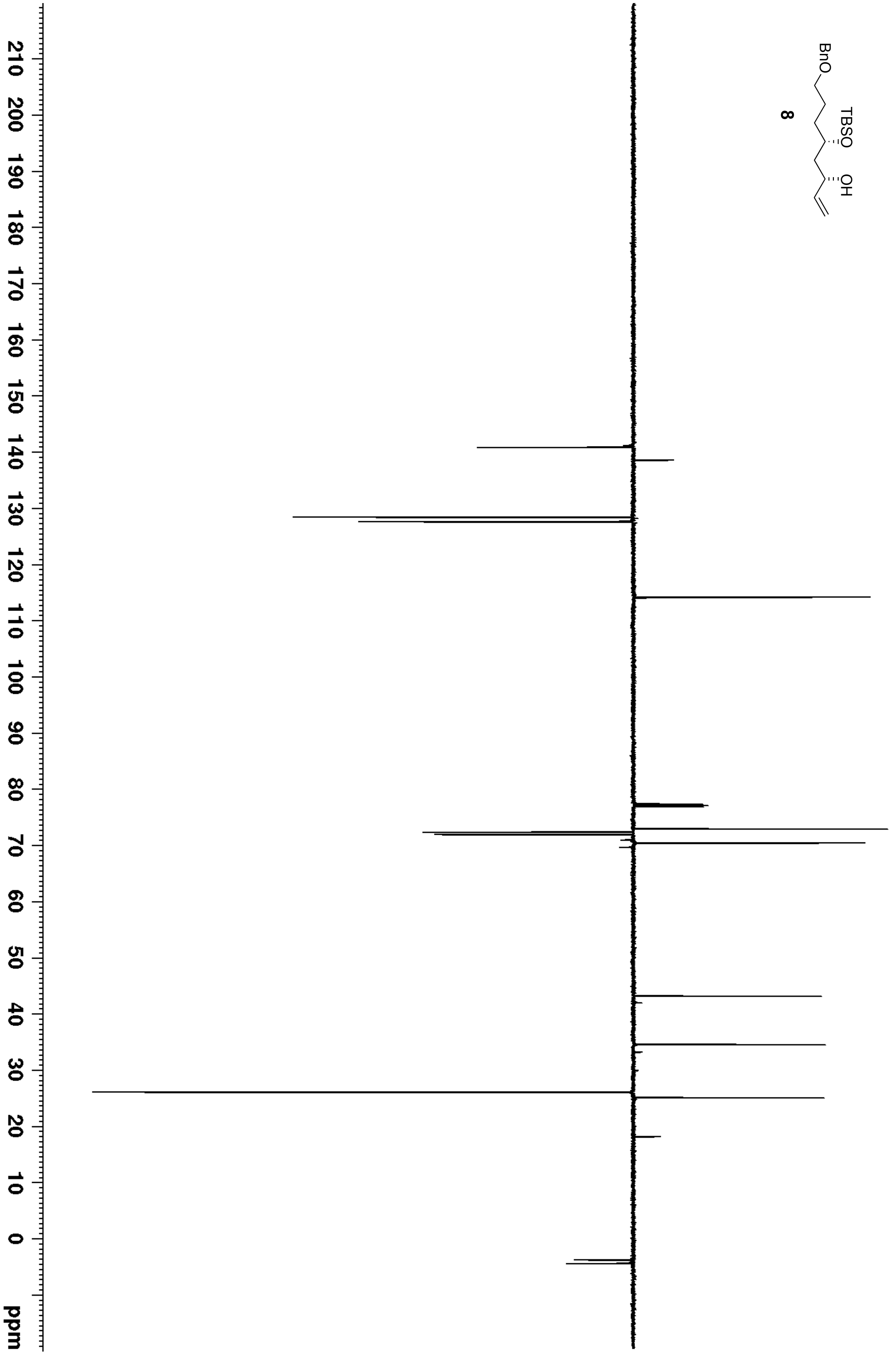
BnO
TBSO
OH
8
210
200
190
180
170
160
150
140
130
120
110
100
90
80
70
60
50
40
30
20
10
0
ppm

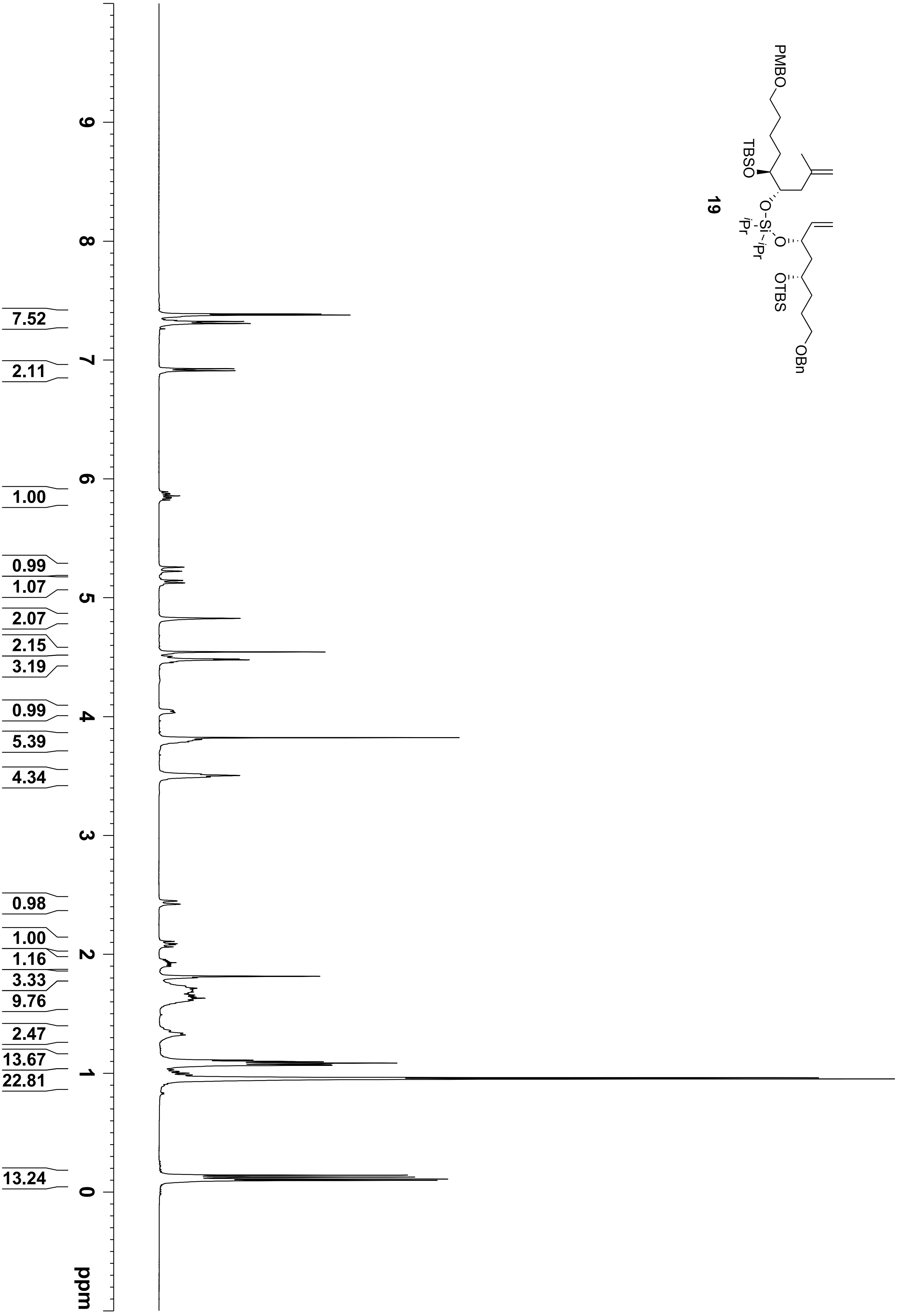
PMBO
TBSO
O-Si-iPr
iPr
O
OTBS
OBn
19
ppm
7.52
2.11
1.00
0.99
1.07
2.07
2.15
3.19
0.99
5.39
4.34
0.98
1.00
1.16
3.33
9.76
2.47
13.67
22.81
13.24

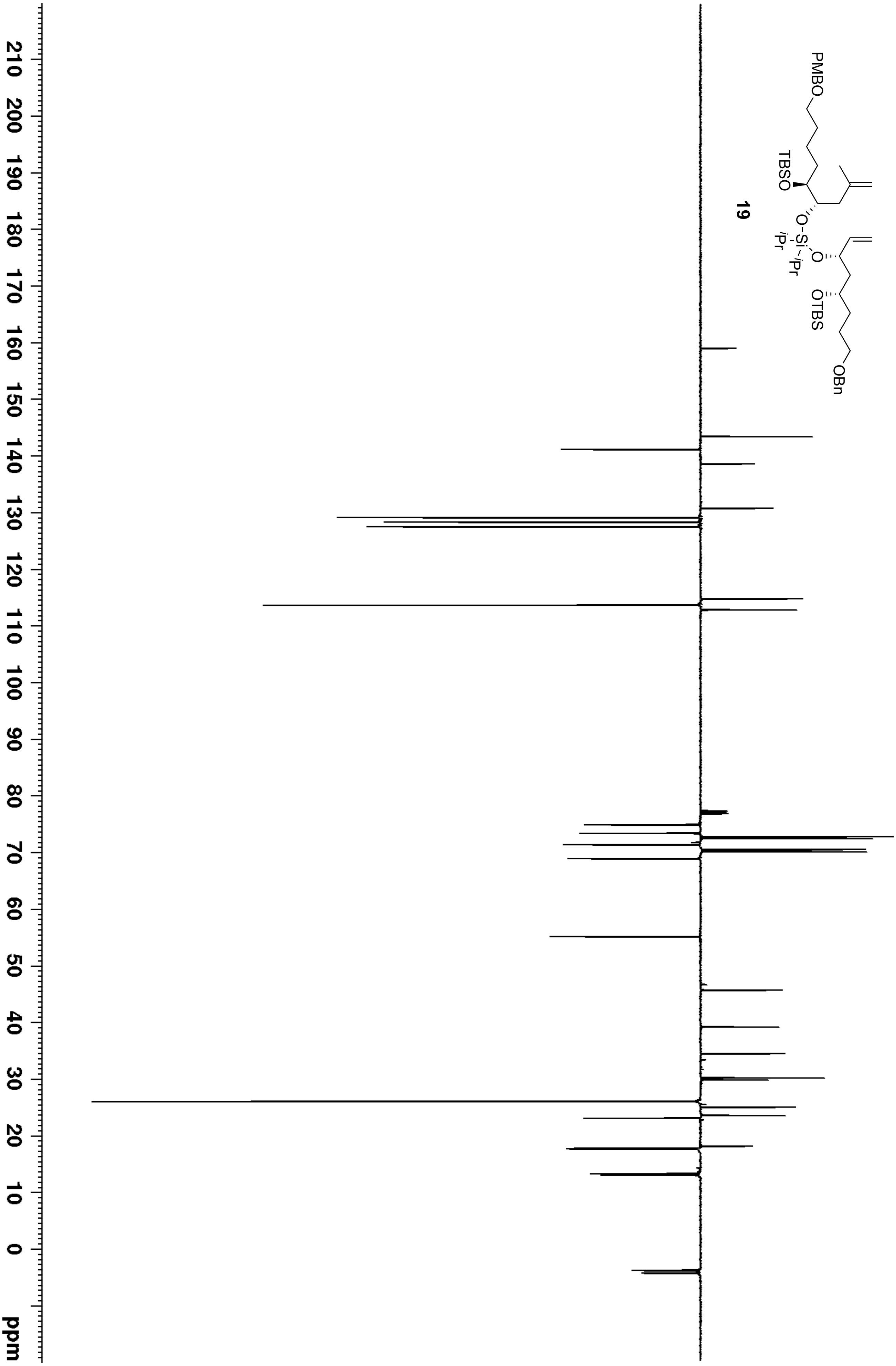
PMBO
TBSO
O-Si-iPr
iPr
O
OTBS
OBn
19
210 200 190 180 170 160 150 140 130 120 110 100 90 80 70 60 50 40 30 20 10 0 ppm

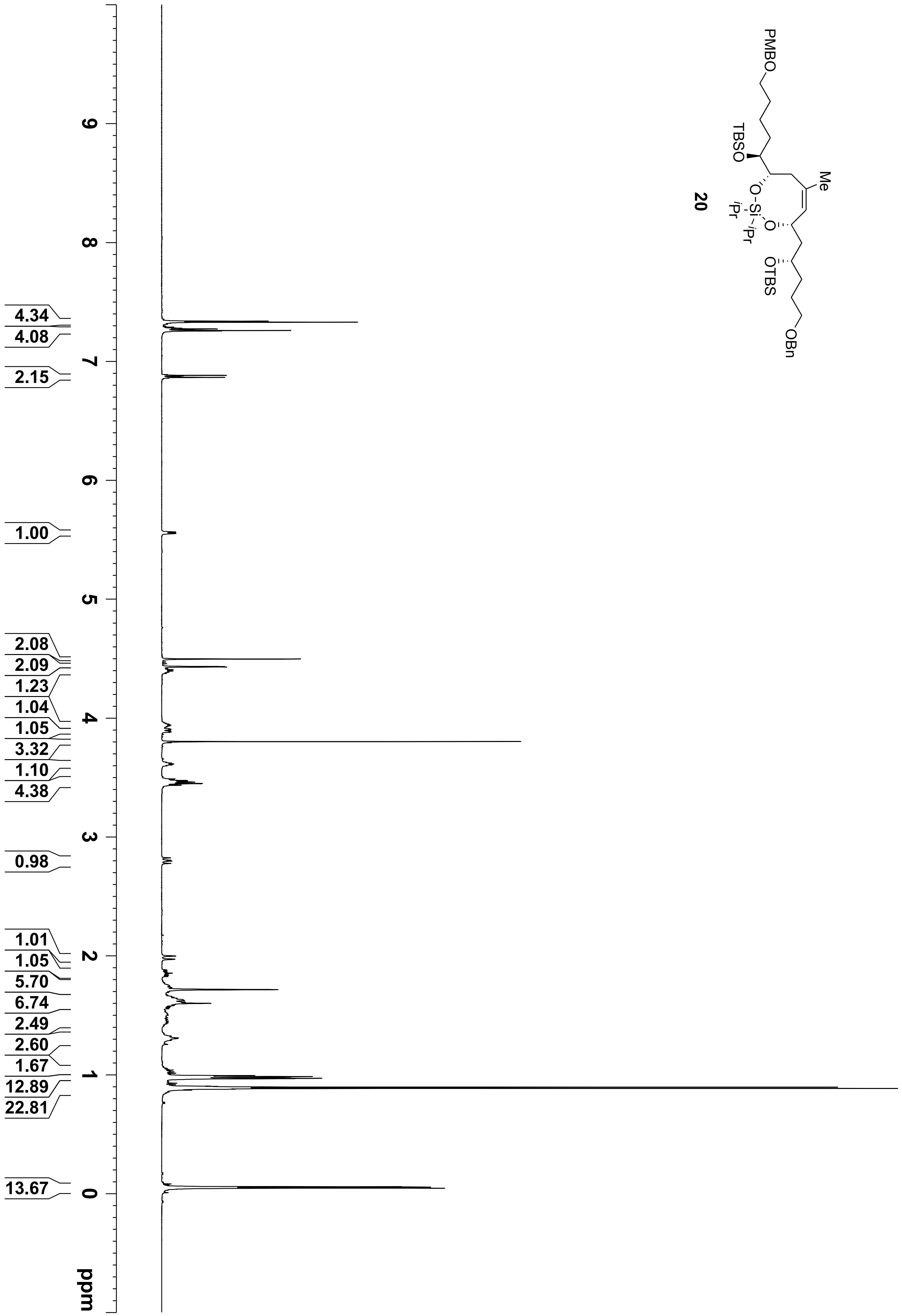
PMBO
TBSO
Me
O-Si-O
iPr
iPr
OTBS
OBn
20
4.34
4.08
2.15
1.00
2.08
2.09
1.23
1.04
1.05
3.32
1.10
4.38
0.98
1.01
1.05
5.70
6.74
2.49
2.60
1.67
12.89
22.81
13.67
9
8
7
6
5
4
3
2
1
0
ppm

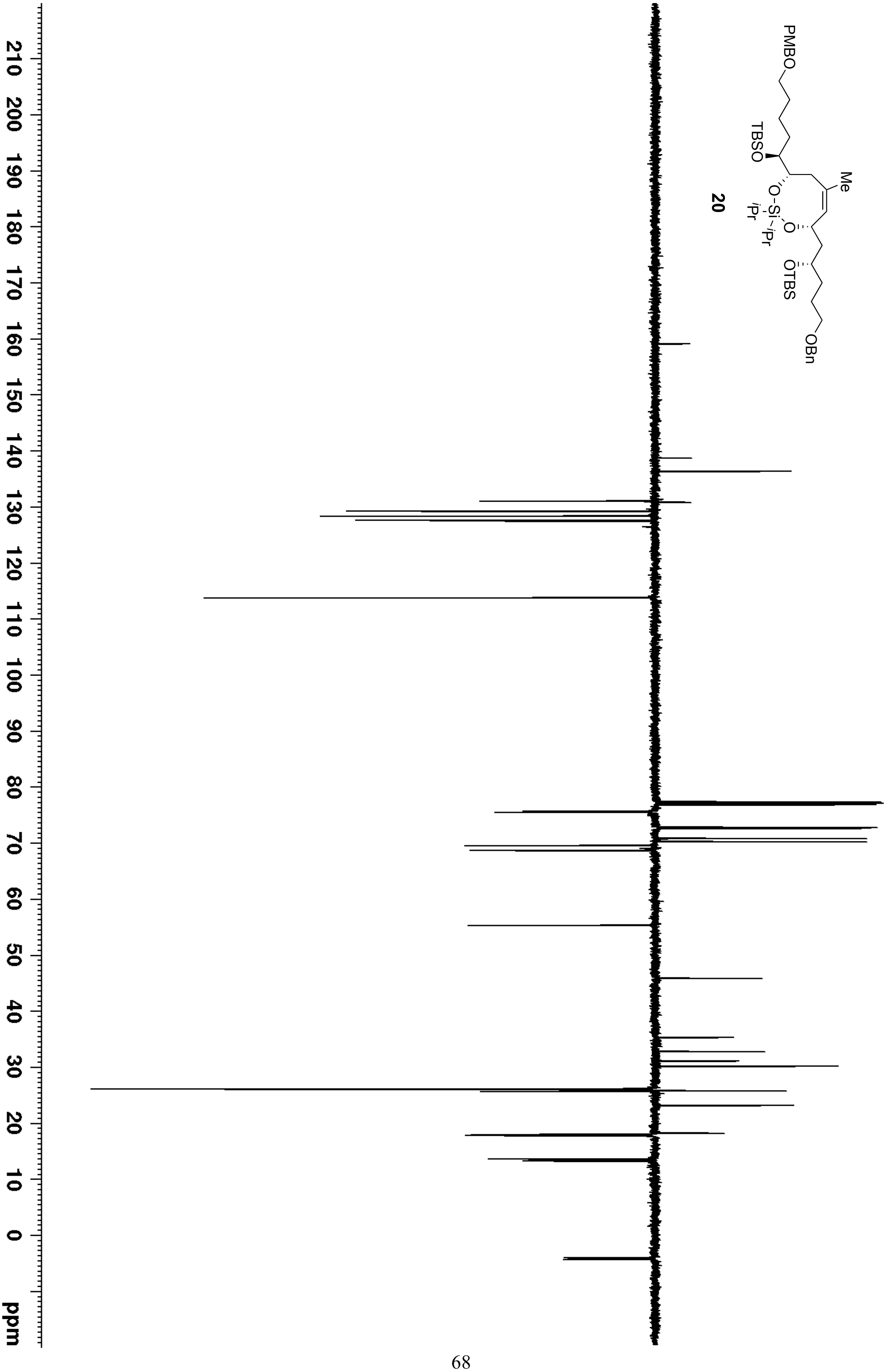
PMBO
TBSO
Me
O–Si–iPr
iPr
O
OTBS
OBn
20
210 200 190 180 170 160 150 140 130 120 110 100 90 80 70 60 50 40 30 20 10 0
ppm

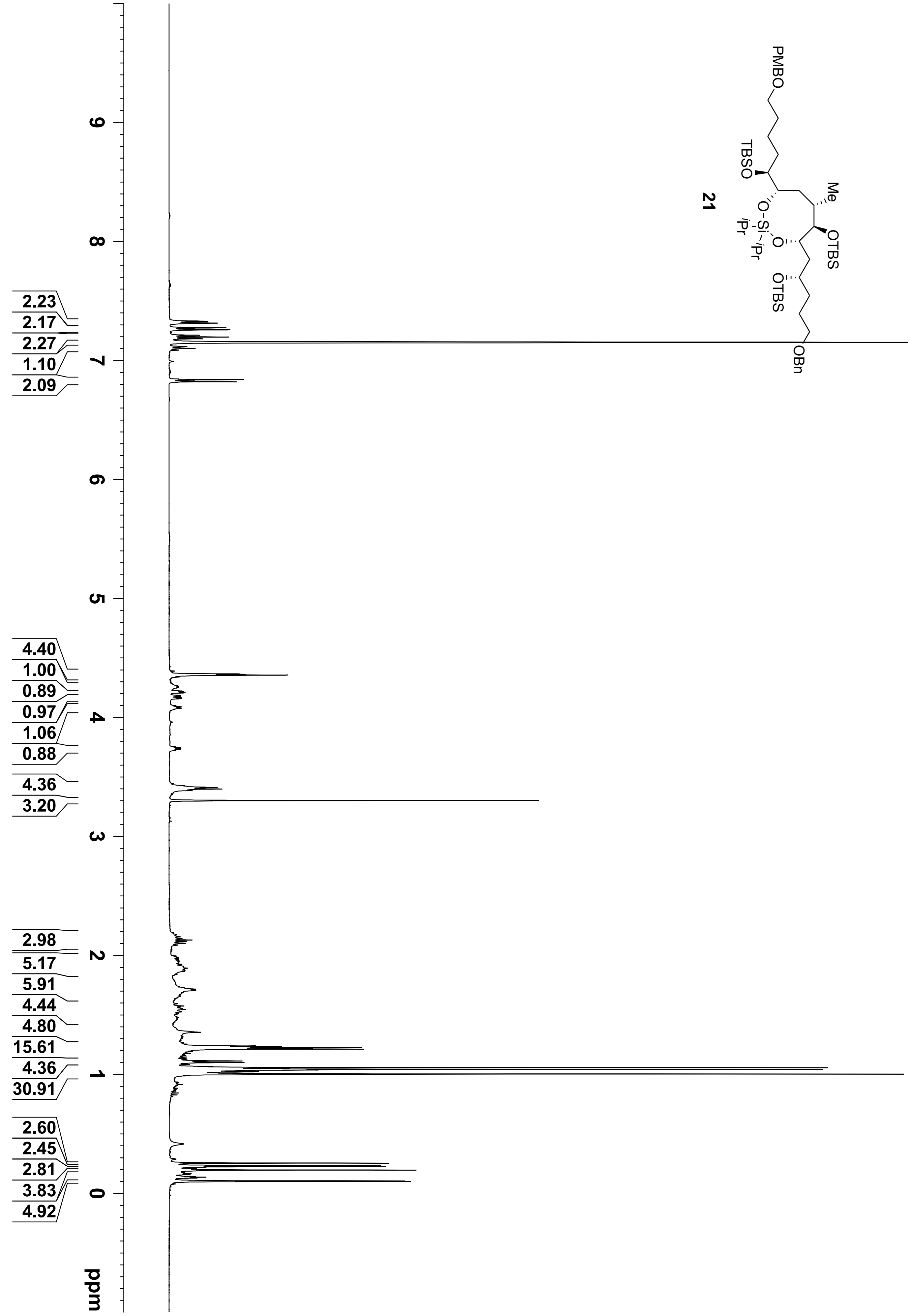

PMBO
TBSO
Me
OTBS
OTBS
OBn
21
ppm

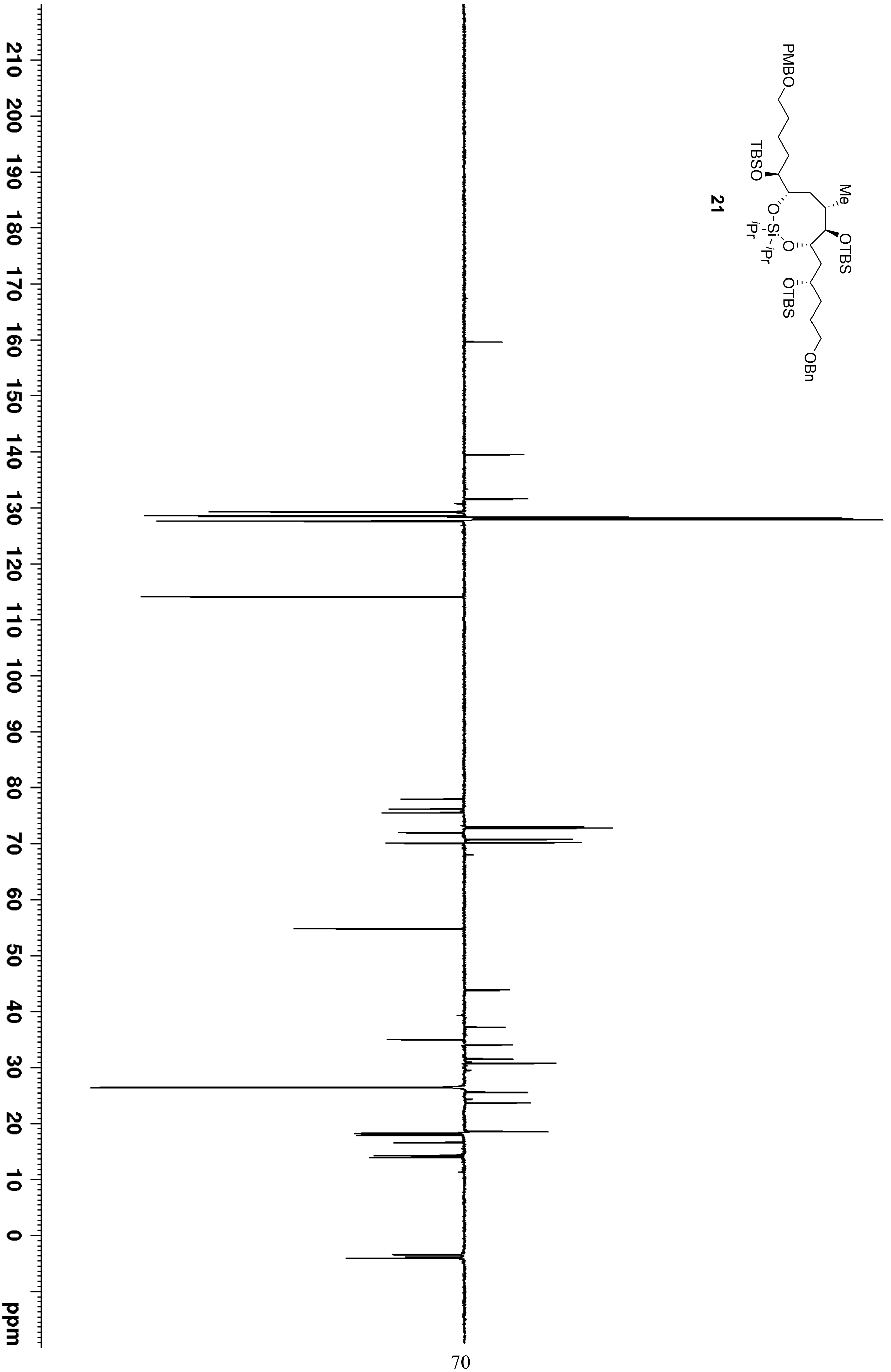
PMBO
TBSO
Me
OTBS
OTBS
OBn
O-Si-iPr
iPr
O
21
210 200 190 180 170 160 150 140 130 120 110 100 90 80 70 60 50 40 30 20 10 0 ppm

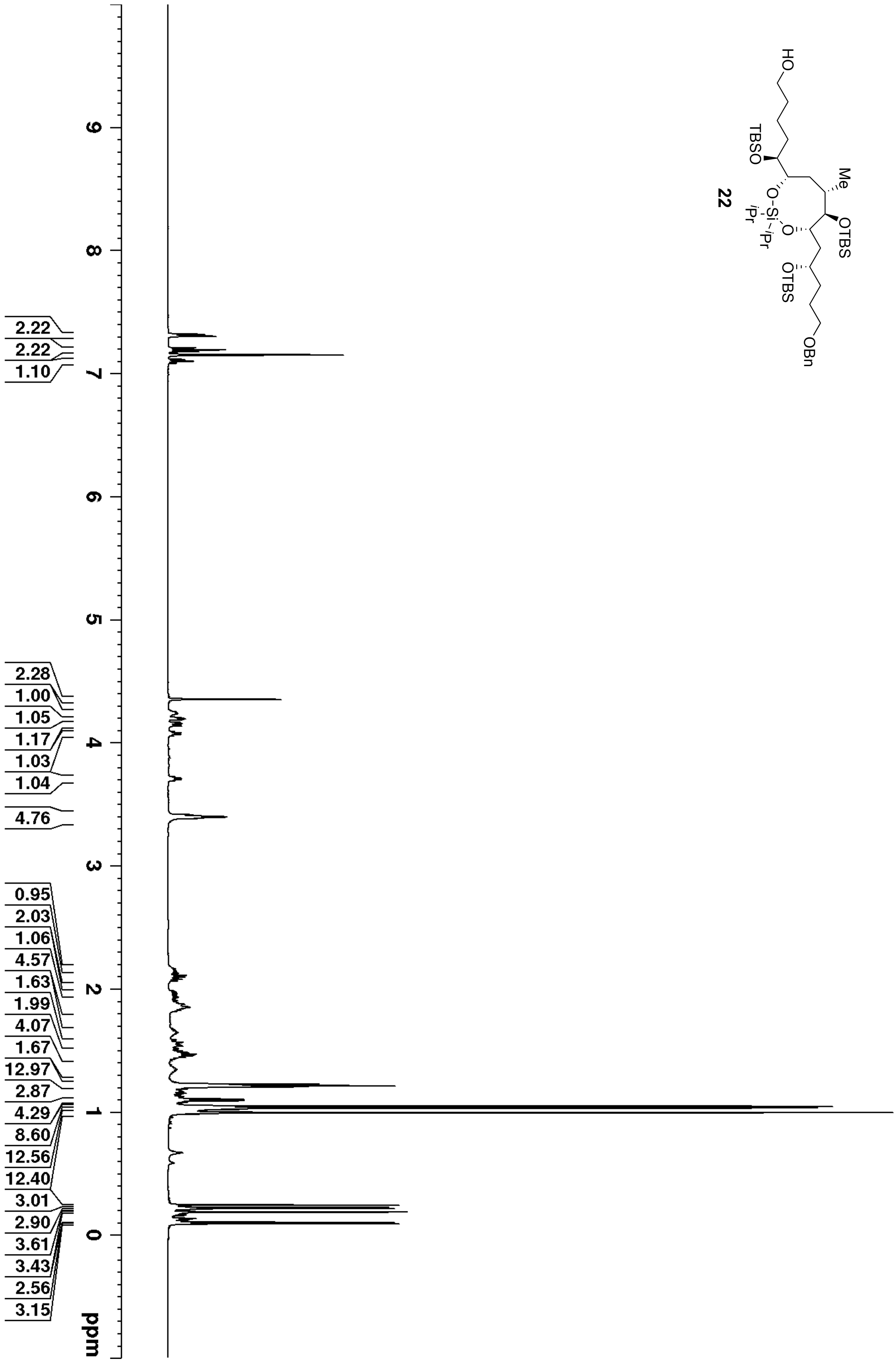
HO
TBSO
Me
OTBS
OTBS
OBn
O-Si-O
iPr
iPr
22
ppm

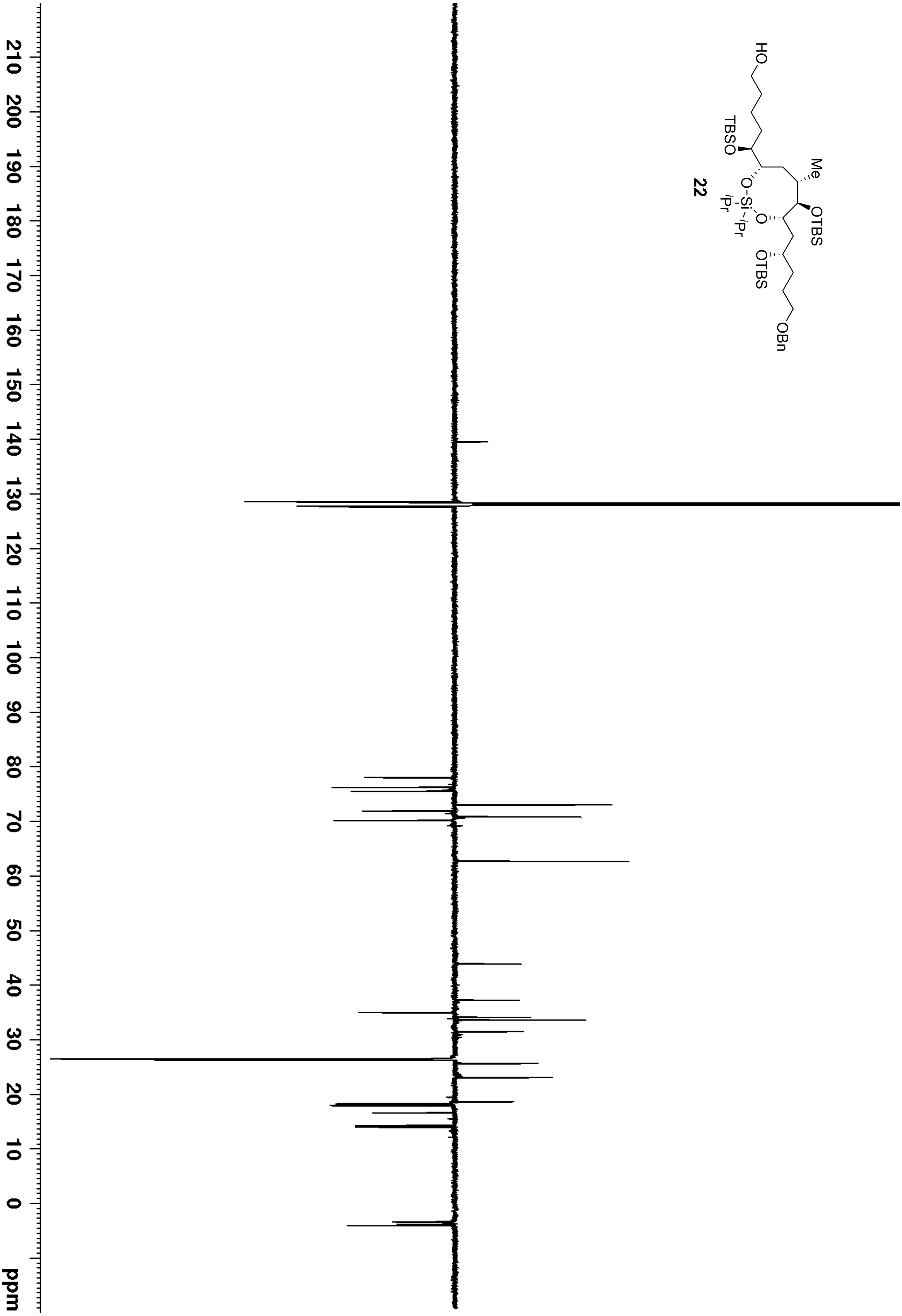
22
HO
TBSO
Me
OTBS
OTBS
OBn
ppm

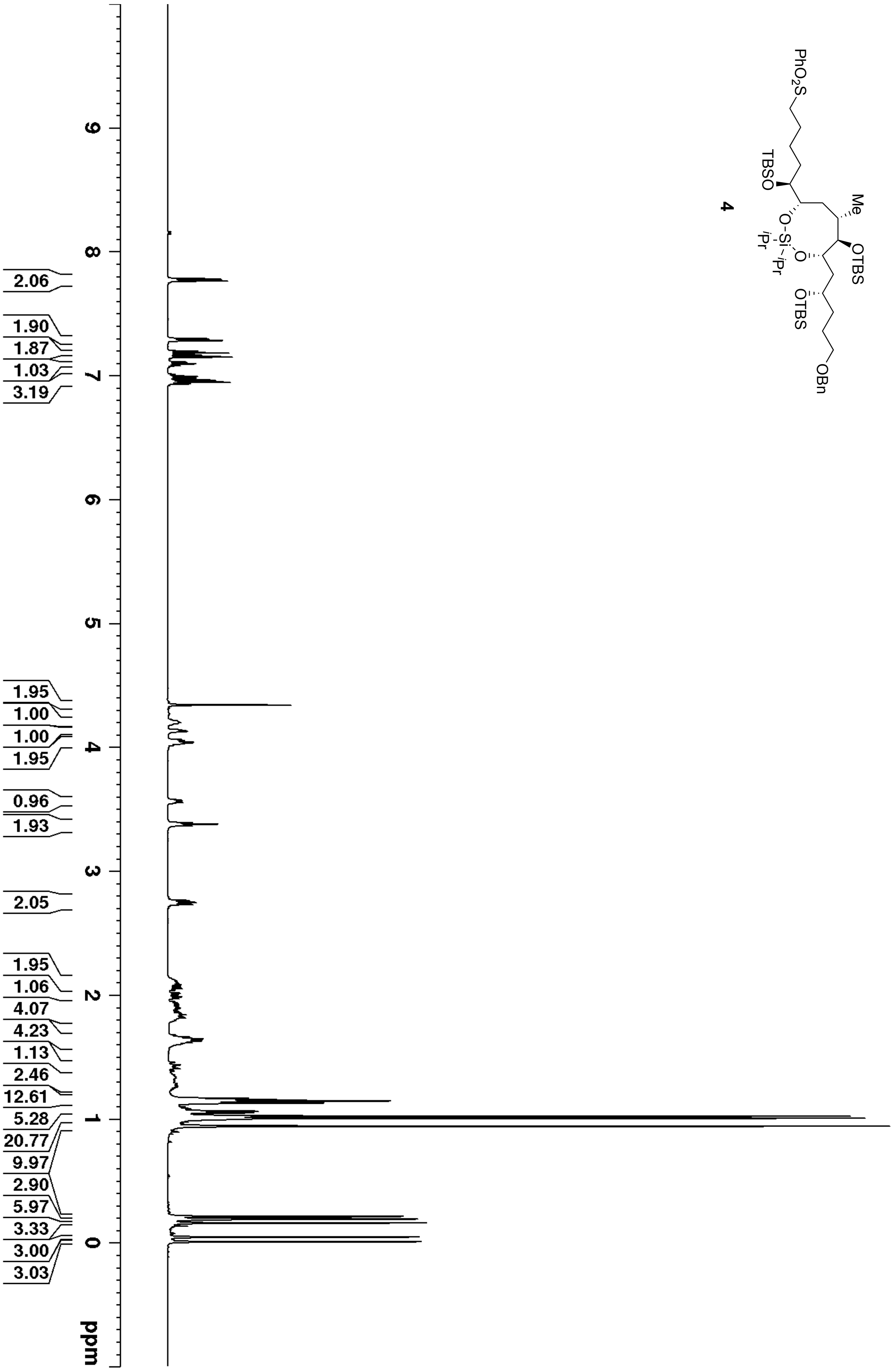

4
PhO2S
TBSO
Me
OTBS
OTBS
OBn
ppm

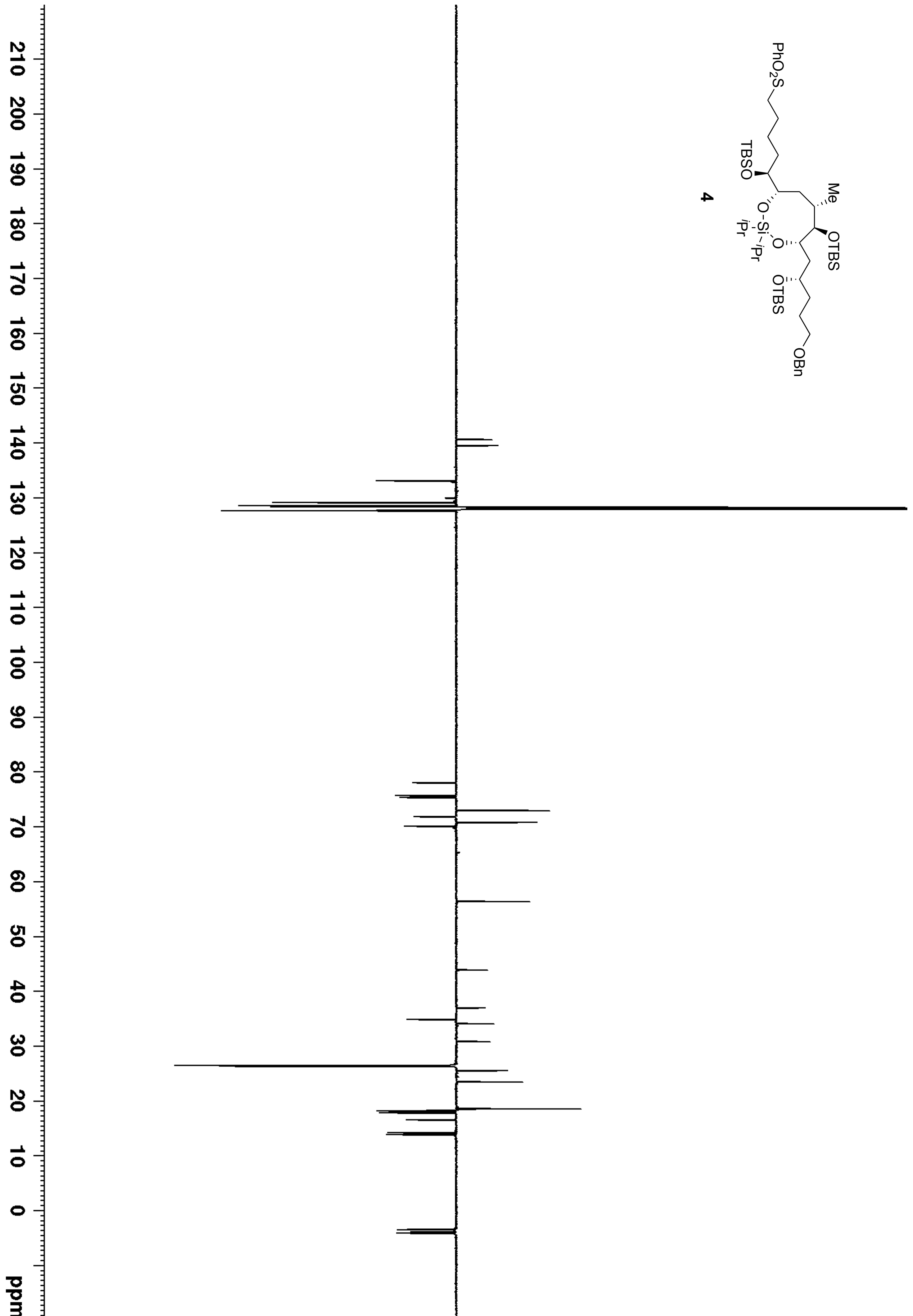
PhO$_2$S
TBSO
Me
OTBS
OTBS
OBn
O-Si-O
iPr
iPr
4
210 200 190 180 170 160 150 140 130 120 110 100 90 80 70 60 50 40 30 20 10 0 ppm

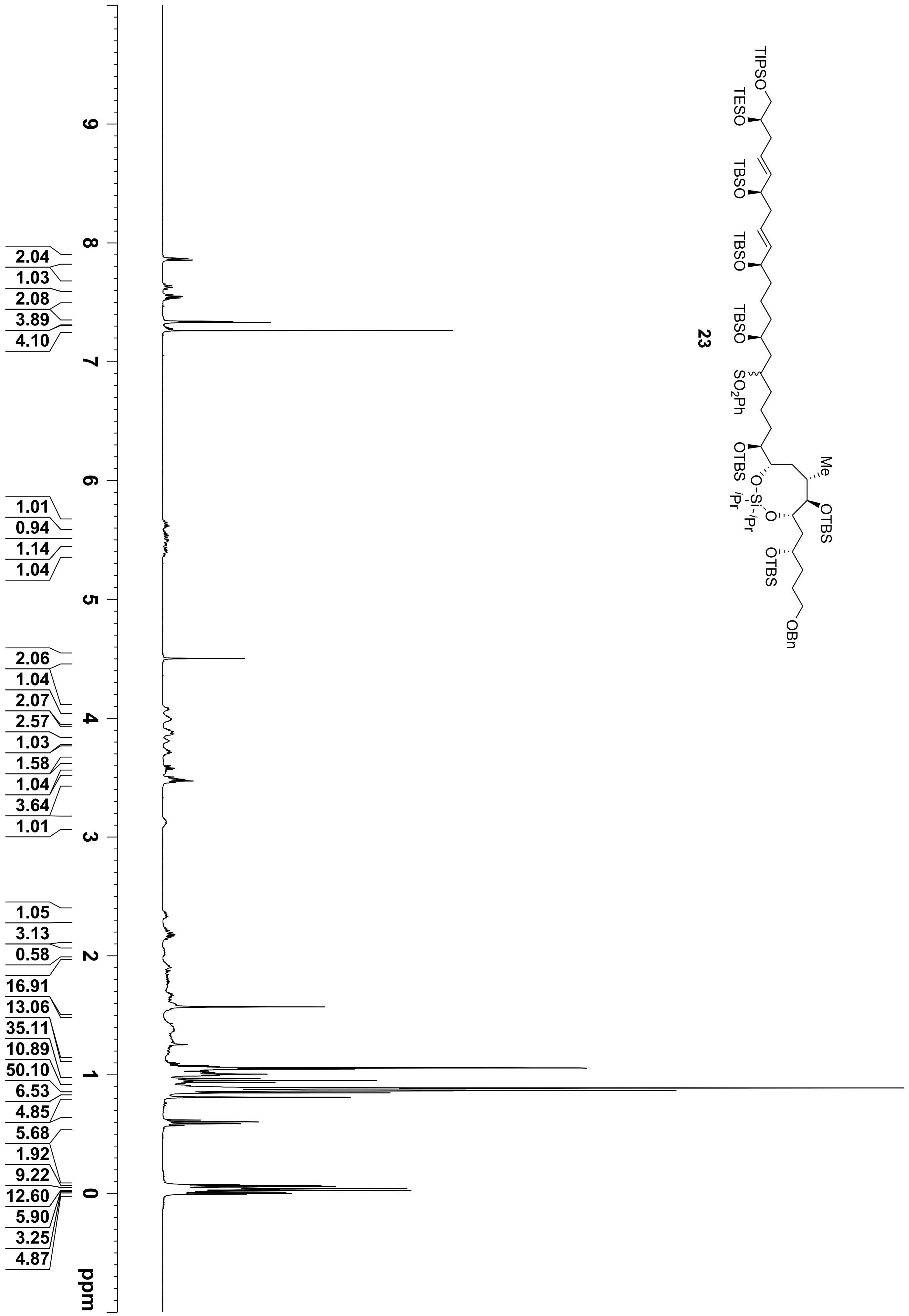
TIPSO
TESO
TBSO
TBSO
TBSO
SO_2Ph
OTBS
Me
OTBS
OTBS
OBn
23
ppm

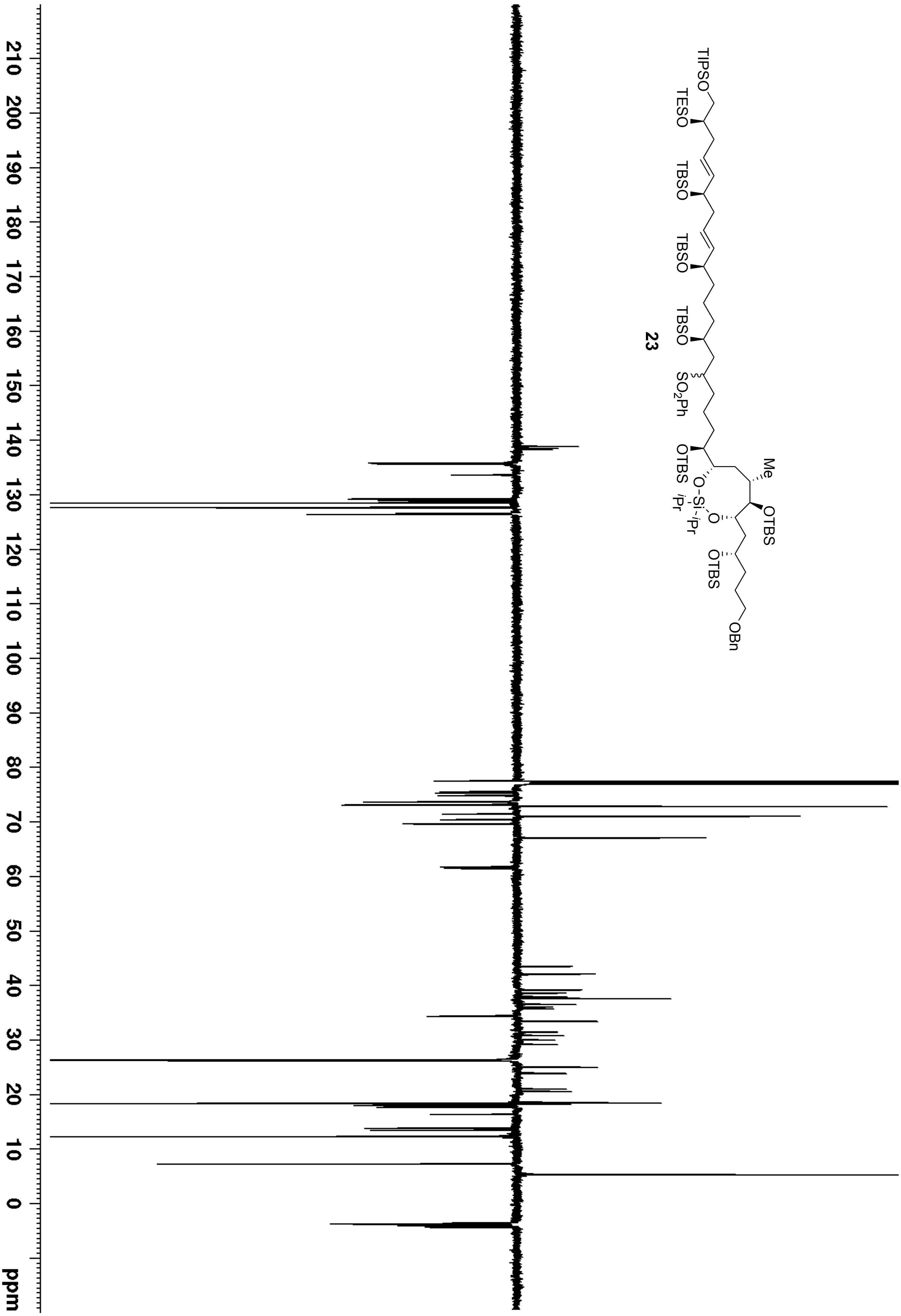

TIPSO
TESO
TBSO
TBSO
TBSO
SO2Ph
OTBS
Me
OTBS
OTBS
OBn
23
ppm

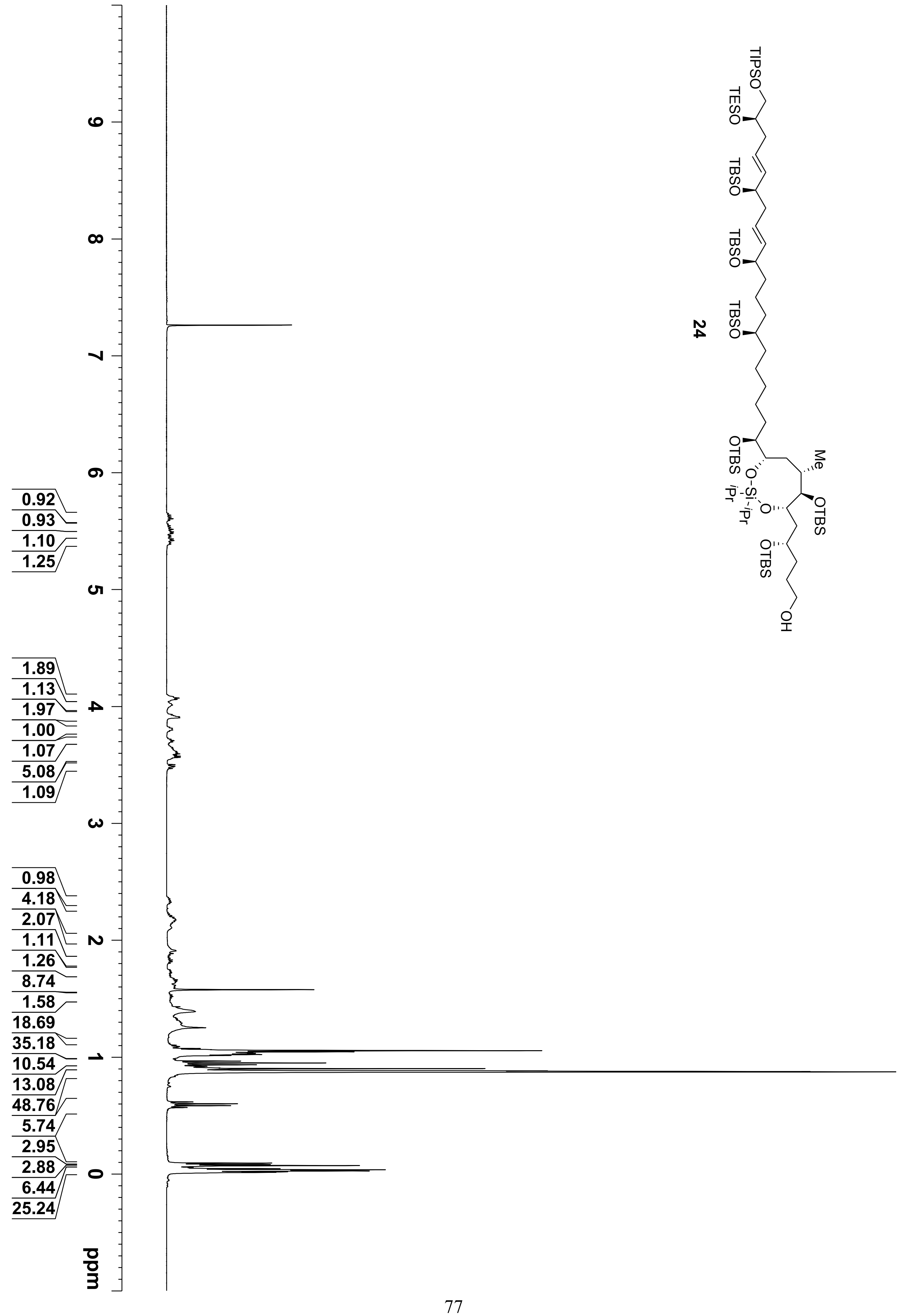
TIPSO
TESO
TBSO
TBSO
TBSO
24
OTBS
Me
OTBS
O-Si-iPr
iPr
OTBS
OH
0.92
0.93
1.10
1.25
1.89
1.13
1.97
1.00
1.07
5.08
1.09
0.98
4.18
2.07
1.11
1.26
8.74
1.58
18.69
35.18
10.54
13.08
48.76
5.74
2.95
2.88
6.44
25.24
9
8
7
6
5
4
3
2
1
0
ppm

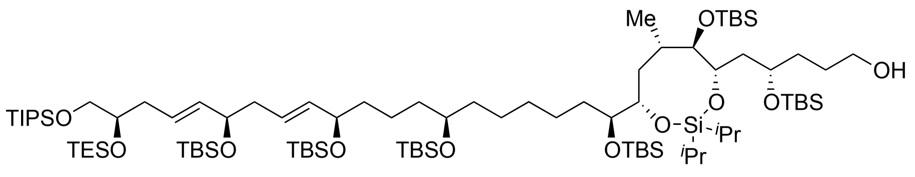

TIPSO
TESO
TBSO
TBSO
TBSO
OTBS
Me
OTBS
O
O-Si
iPr
iPr
OTBS
OH

24

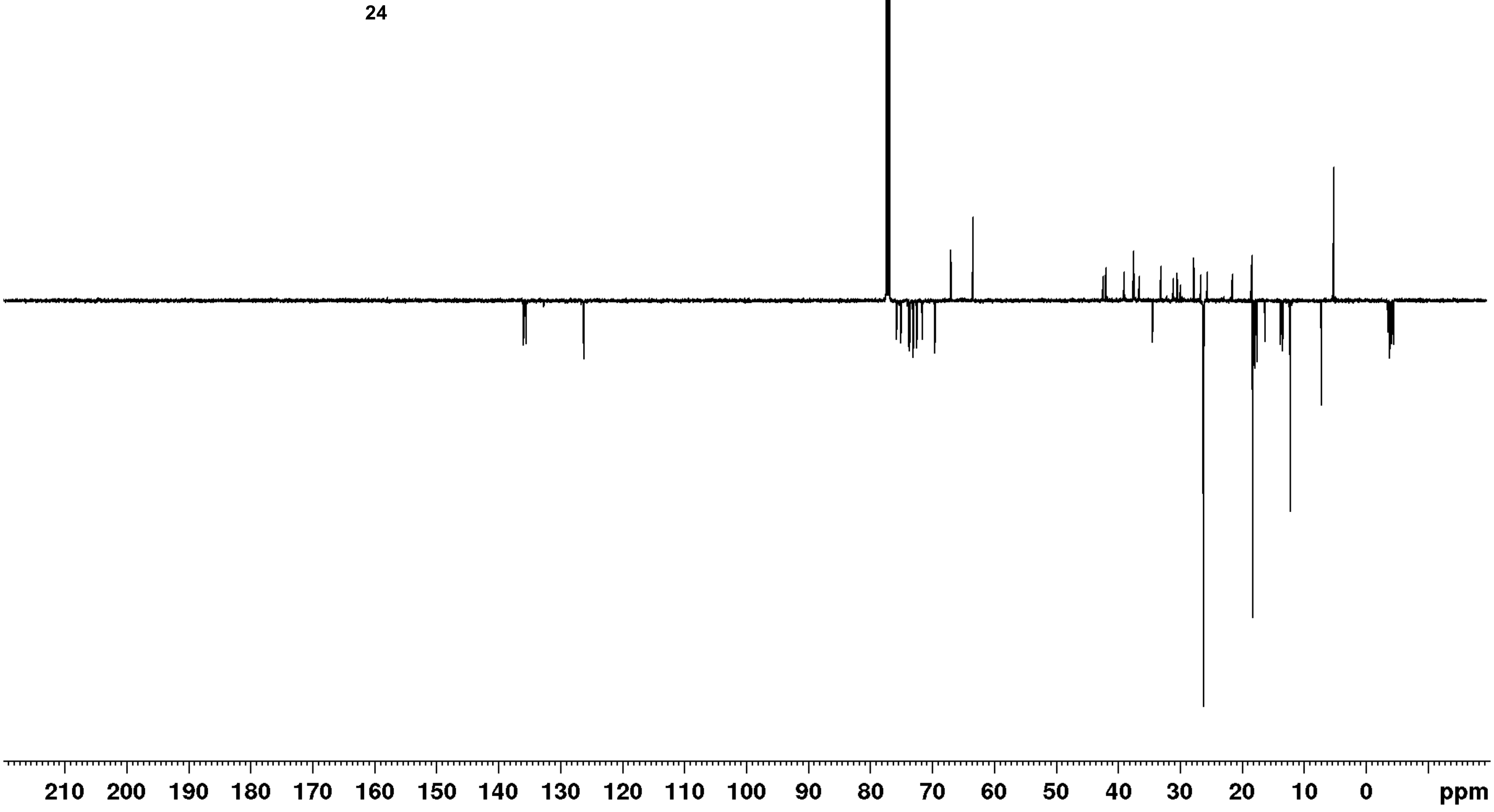

210 200 190 180 170 160 150 140 130 120 110 100 90 80 70 60 50 40 30 20 10 0 ppm

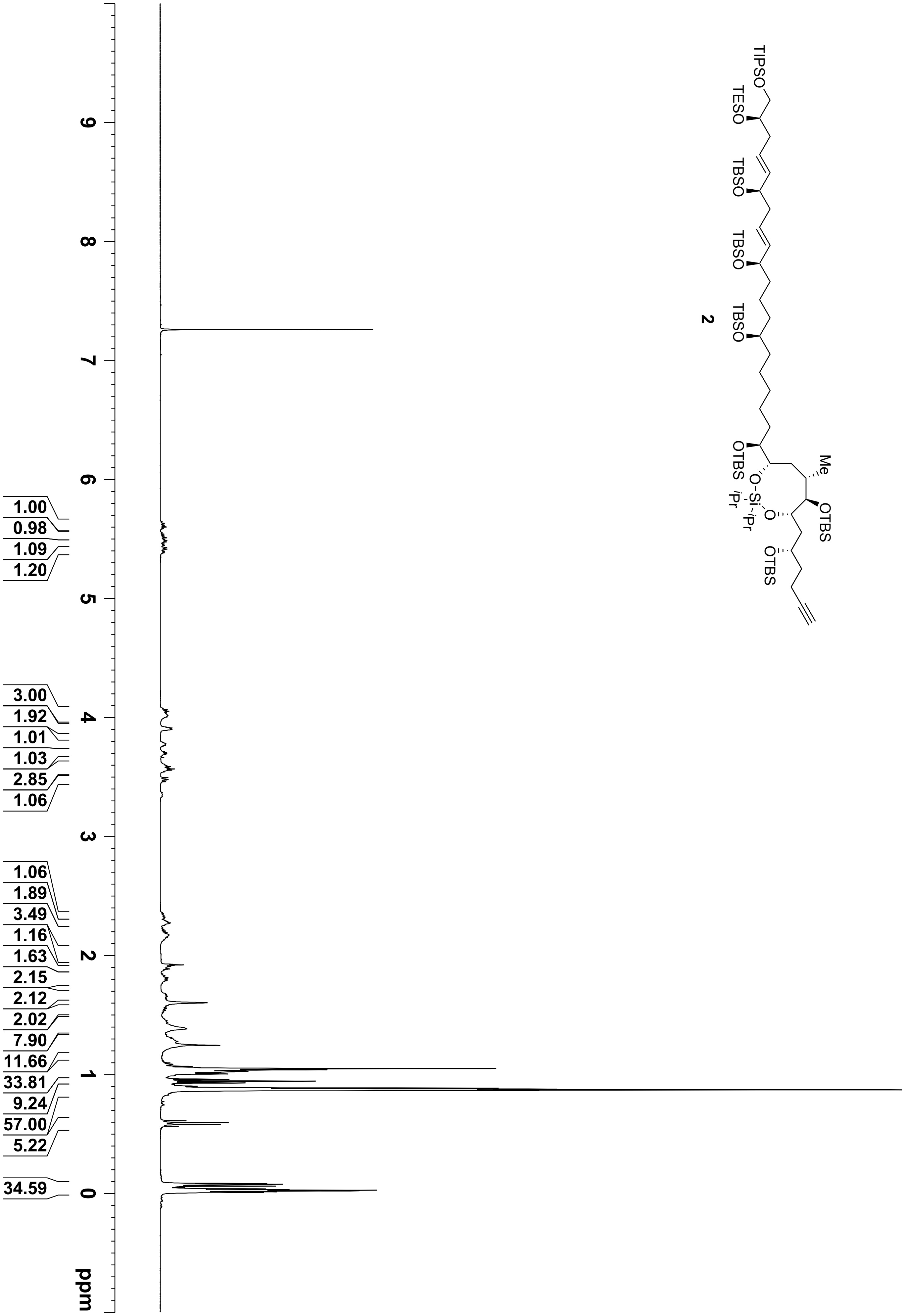
TIPSO
TESO
TBSO
TBSO
TBSO
OTBS
Me
OTBS
OTBS
2
ppm

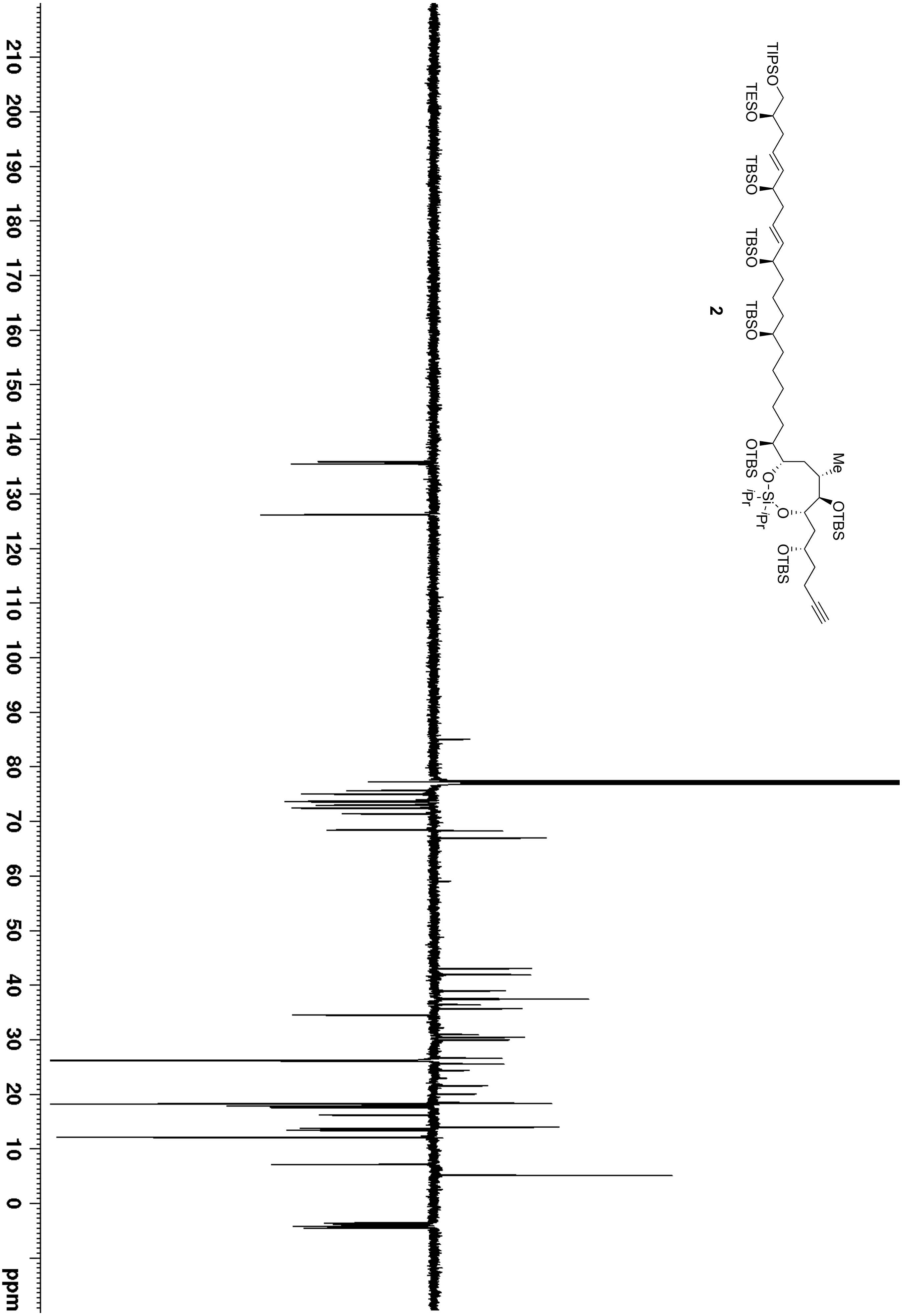
TIPSO
TESO
TBSO
TBSO
TBSO
OTBS
Me
OTBS
OTBS
O-Si-iPr
iPr
2
210 200 190 180 170 160 150 140 130 120 110 100 90 80 70 60 50 40 30 20 10 0 ppm

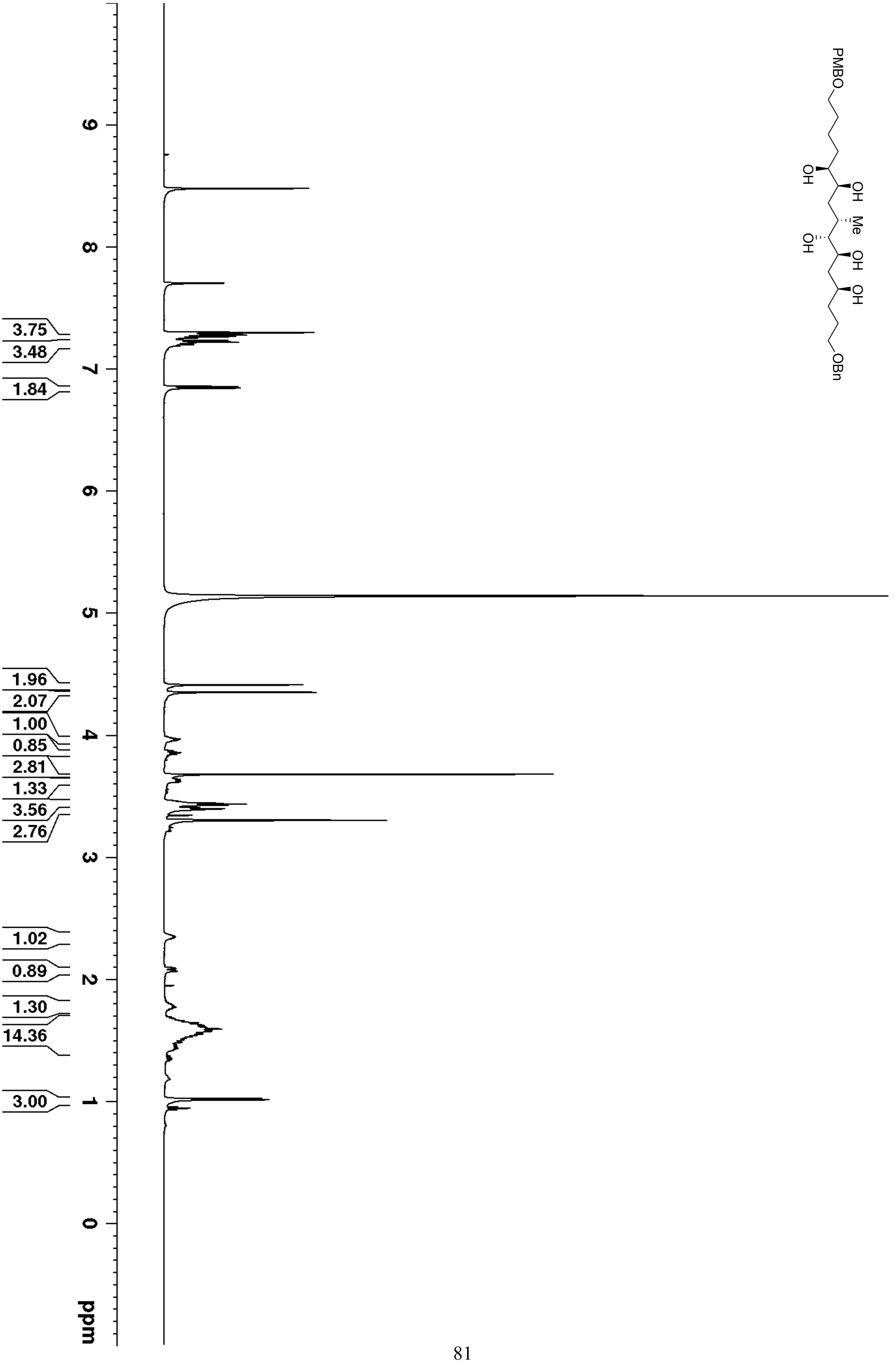
PMBO
OH
OH
Me
OH
OH
OH
OBn
3.75
3.48
1.84
1.96
2.07
1.00
0.85
2.81
1.33
3.56
2.76
1.02
0.89
1.30
14.36
3.00
9
8
7
6
5
4
3
2
1
0
ppm

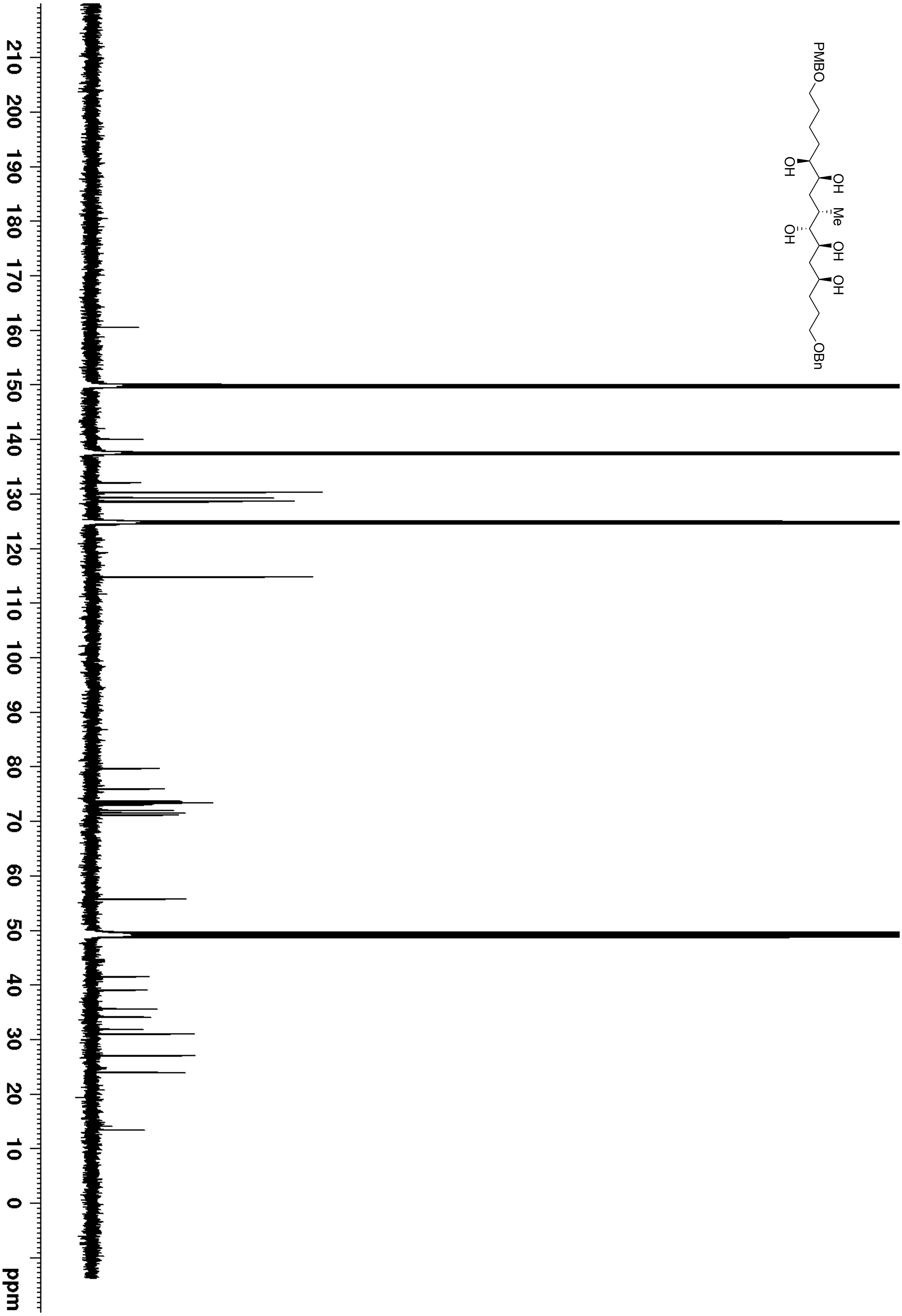
PMBO
OBn
OH
Me
ppm

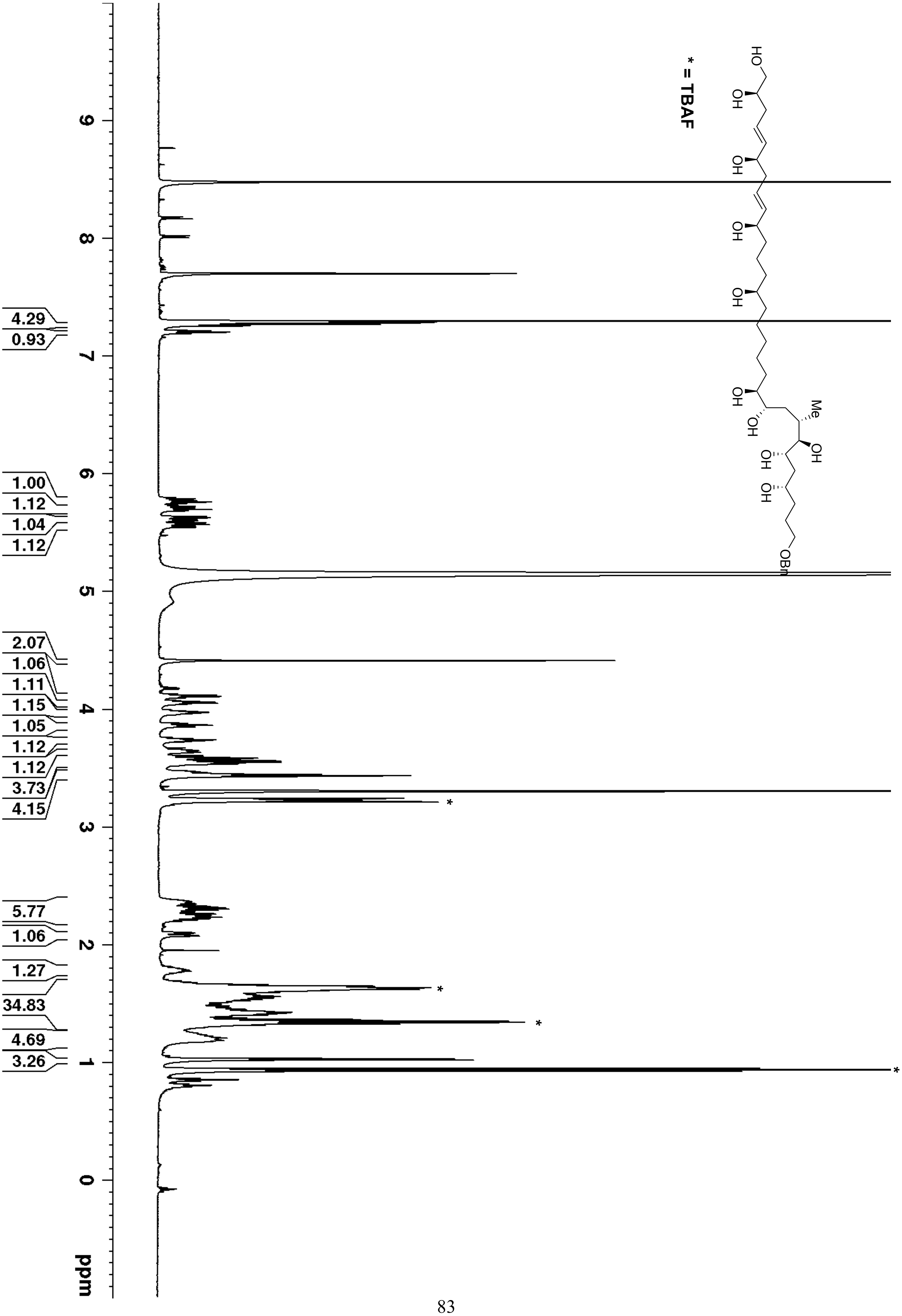
* = TBAF
ppm
9
8
7
6
5
4
3
2
1
0
4.29
0.93
1.00
1.12
1.04
1.12
2.07
1.06
1.11
1.15
1.05
1.12
1.12
3.73
4.15
5.77
1.06
1.27
34.83
4.69
3.26
OBn
Me

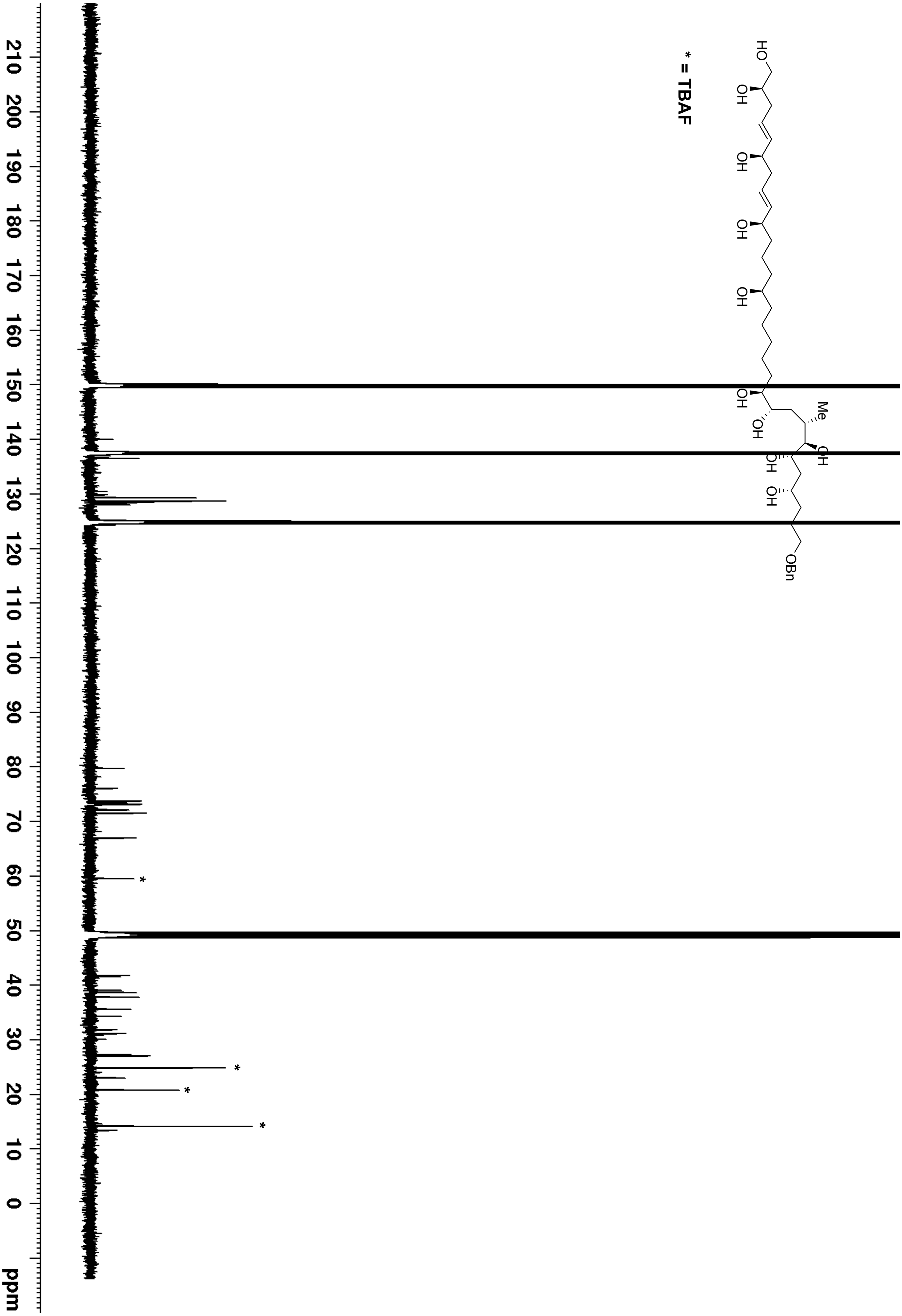
* = TBAF
ppm
OBn
Me
OH
HO
